# Synthesis, Spectroscopic Characterization, Molecular Docking Studies Toward the GABA_A_ Receptor, ADMET Prediction, GABA Aminotransferase Inhibition, and Anxiolytic Evaluation of Novel Thiazine Derivatives

**DOI:** 10.3390/ph19091364

**Published:** 2026-08-28

**Authors:** Sonu, Kamal Y. Thajudeen, Saad Ali Alshehri, Mohammed Muqtader Ahmed, Mayur Porwal, Sagar Verma

**Affiliations:** 1 Drug Design Laboratory, School of Pharmaceutical Sciences, IFTM University, Moradabad 244102, India; 2 Department of Pharmaceutical Chemistry, Shri Ram College of Pharmacy, Muzaffarnagar 251001, India; 3 Department of Pharmacognosy, College of Pharmacy, King Khalid University, Abha 62529, Saudi Arabia; 4 Department of Pharmaceutics, College of Pharmacy, Prince Sattam Bin Abdulaziz University, AlKharj 11942, Saudi Arabia; 5 Teerthanker Mahaveer College of Pharmacy, Teerthanker Mahaveer University, Moradabad 244001, India

**Keywords:** chalcone, anxiety, ADMET, docking, anxiolytic activity

## Abstract

**Background:** The current study uses a computational technique to investigate synthesized thiazine compounds that exhibit potential interactions with the GABA_A_ receptor. A series of novel thiazine derivatives was synthesized via the cyclization reaction of chalcone with thiourea. **Methods:** For the synthesized compounds, spectroscopic characterization, namely FT-IR, ^1^H NMR, ^13^C NMR, and mass spectrometry, was carried out. In addition, the AutoDock Vina 4.2 software was applied to carry out an in silico docking study. **Results:** The synthesized compounds C3 and C6 exhibited comparable predicted docking scores (−9.0 and −9.1 kcal/mol, respectively), with favorable predicted interactions at the GABAA receptor binding site. Furthermore, in silico ADMET analysis provided insights into the drug-likeness and pharmacokinetic profiles of the synthesized compounds; however, potential safety liabilities, including Ames positivity, hERG II liability, and hepatotoxicity, were predicted for some derivatives. **Conclusions:** It was determined that the synthesized compounds’ various physiologically significant and physiologically relevant parameters fell within the range of Lipinski’s rule of five. The synthesized compounds (C1–C10) were tested for anxiolytic activity by the elevated plus maze test, and compound C6 was found to be the most potent. Additionally, the in vitro GABA-AT enzyme activity assay showed that compound C6 had the highest inhibitory potential, with an IC50 of 13.29 ± 1.622 µM.

## 1. Introduction

Heterocyclic chemistry is a significant discipline of chemistry that involves the synthesis, properties, and applications of heteromolecules [1]. Heterocyclic compounds are prevalent in nature and are vital for life [2]. Heterocyclic compounds constitute a significant category of natural and synthetic compounds, serving crucial functions across multiple domains, particularly in medical chemistry. These chemicals have garnered considerable attention from researchers, especially in the biomedical domain, owing to their substantial therapeutic potential. In this extensive category of compounds, five- and six-membered ring heterocycles with one or more heteroatoms, including nitrogen, oxygen, or sulfur, are crucial in the metabolic processes of living cells [3]. With nitrogen as a heteroatom, heterocyclic chemistry is regarded as an important and unique class of organic chemistry, with a great deal of study devoted to the creation of new compounds. Data show that about 85% of all chemical substances that are physiologically active contain heterocycles. This highlights how important heterocycles are to contemporary medication design [4]. Chalcone (trans-1,3-diphenyl-2-propen-1-one) is an α,β-unsaturated ketone that has the skeletal structure of so-called chalcones. Chalcones are also known as anthochlor pigments [5]. The Claisen-Schmidt condensation reaction, which includes substituted benzaldehyde and acetophenone followed by a strong base, is typically how chalcones are made [6]. Natural substances called chalcones are present in plants and have drawn a lot of interest because of their wide range of biological functions [7]. They may be able to manage diabetes, treat cancer, and lessen inflammation [8]. By altering different signaling pathways and enzymes, these substances use their medicinal qualities to produce various therapeutic outcomes [9]. Remarkably, chalcones can also be found in several foods, such as onions, apples, and green tea [10]. According to certain research, including chalcones in our diet may have health advantages, such as lowering our risk of cardiovascular illnesses. Overall, chalcones offer promising prospects for developing new medications and improving medical outcomes [11].

Recent reports have demonstrated that chalcone-derived heterocyclic compounds, including thiazine analogues, exhibit significant central nervous system activities such as anticonvulsant and anxiolytic effects [12]. For instance, various thiazine derivatives synthesised via cyclisation of chalcone intermediates have shown promising pharmacological profiles in preliminary biological evaluations [13]. Additionally, several heterocyclic scaffolds targeting the GABAergic system have been reported to modulate anxiety-related pathways effectively [14]. However, despite these advancements, the development of chalcone-derived thiazine compounds specifically targeting the GABA_A_ receptor for anxiolytic activity remains relatively unexplored.

Anxiety disorders represent a major and growing psychiatric concern, with increasing prevalence associated with chronic stress and behavioral changes, including those observed during the SARS-CoV-2 pandemic. Although conventional anxiolytic drugs are widely used, their clinical utility may be limited by adverse effects, variable efficacy, and other treatment-related limitations. Therefore, there is a continuing need to identify effective and better-tolerated therapeutic options [15,16].

γ-aminobutyric acid (GABA) regulates the excitation of numerous central nervous system pathways by acting on the GABA-A chloride ion channel (GABA_A_ receptor). Benzodiazepines, neurosteroids, and barbiturates are among the categories of chemical compounds that interact through this macromolecular ionophore and transform the accomplishment of GABA on the neuronal chloride flux. The intrinsic action of these chemical compounds varies from full agonists such as hypnotics, anxiolytic, and anticonvulsant agents to inverse agonists. This spectrum includes partial agonists, which may lessen familiar benzodiazepine-mediated adverse effects such as muscular relaxation, forgetfulness, physical dependency, and over-sedation. Since it would be beneficial to identify partial agonists for the GABA_A_ receptor’s benzodiazepine binding site for medicinal purposes, different teams of research have prepared and studied such ligands with various chemical structures, including imidazopyridine (e.g., Alpidem), β-carboline (e.g., Abecarnil), quinoxalinone (e.g., Panadiplon), imidazoquinoxaline, and pyridobenzimidazole structures. Additionally, recent molecular biology research has shown that the GABA_A_ receptor is a heterooligomeric ligand-gated chloride channel made up of α-, β-, γ-, and δ-subunits, which has sparked new interest in finding new selective ligands for specific GABA_A_ receptor subtypes [17,18,19]. Based on the reported pharmacological significance of chalcone and thiazine scaffolds, a series of novel thiazine derivatives (C1–C10) bearing different electron-donating and electron-withdrawing substituents were designed and synthesized to investigate the influence of structural modifications on their molecular interactions, GABA-AT inhibitory activity, and anxiolytic-like potential. The selected substituents (NO_2_, Cl, Br, OH, OCH_3_, and N(CH_3_)_2_ were intentionally incorporated to investigate the influence of their electronic and physicochemical properties on the biological activity of the synthesized thiazine derivatives. Electron-withdrawing groups such as NO_2_, Cl, and Br may enhance hydrophobic interactions and influence electron distribution within the scaffold, whereas electron-donating groups such as OH, OCH_3_, and N(CH_3_)_2_ can modify hydrogen-bonding ability, electronic density, and molecular polarity. These structural variations were introduced to facilitate a preliminary structure–activity relationship (SAR) analysis and to evaluate their effects on molecular docking, GABA-AT inhibition, and anxiolytic-like activity. Furthermore, comparison of the biological results among the synthesized analogues enabled a preliminary SAR, suggesting that the nature and position of the substituents influenced both the predicted binding interactions and the observed biological activity.

In the present study, the synthesized compounds were structurally characterized using FT-IR, ^1^H NMR, ^13^C NMR, and Mass spectrometry. Furthermore, molecular docking studies, ADMET prediction, GABA aminotransferase (GABA-AT) inhibition assay, and in vivo anxiolytic-like evaluation using the Elevated Plus Maze model were performed to investigate their pharmacological potential.

## 2. Results and Discussion

### 2.1. Synthesis and Chemistry

**Scheme 1:**
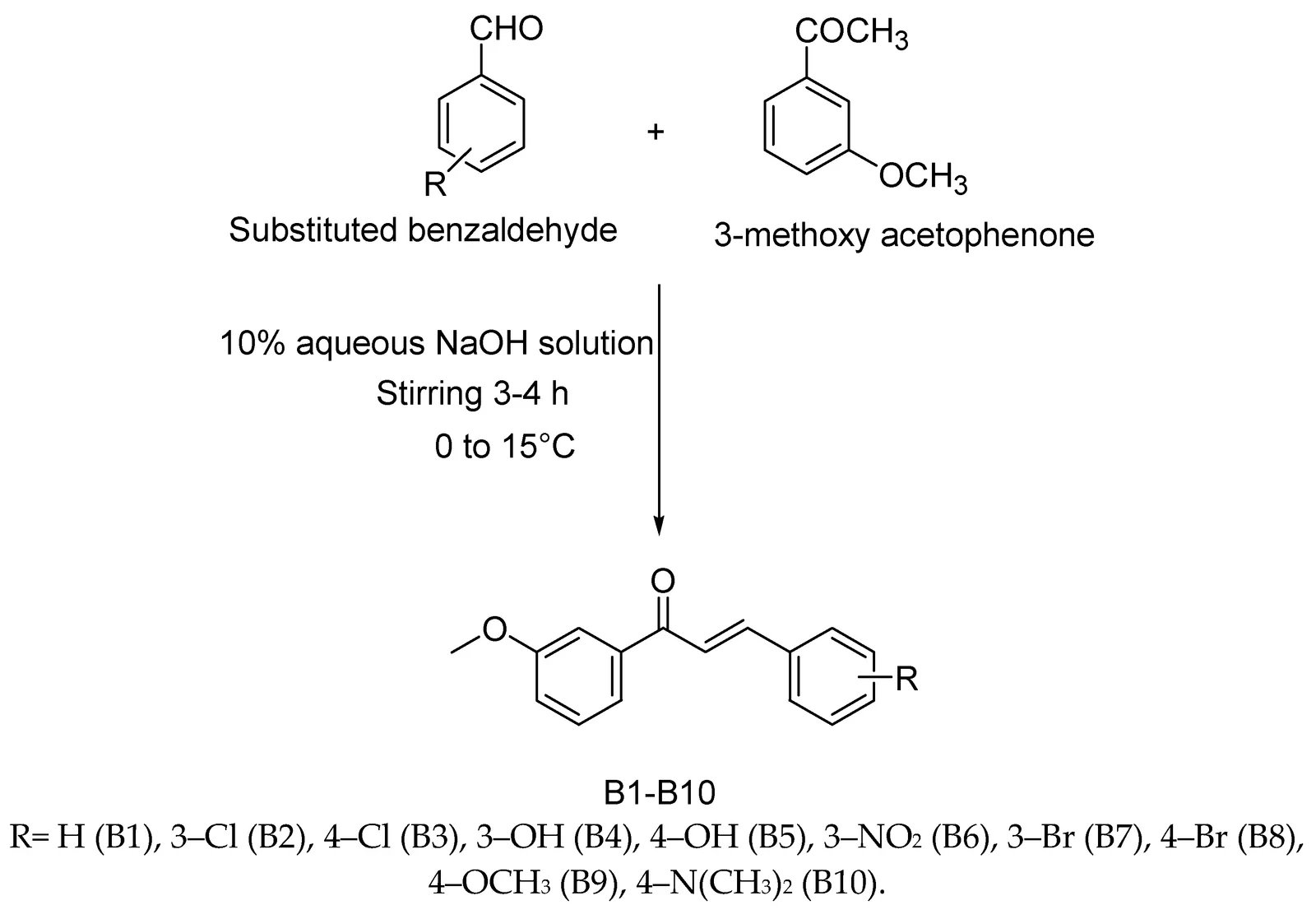
Synthetic route for the preparation of chalcone intermediates (**B1**–**B10**).

**Scheme 2:**
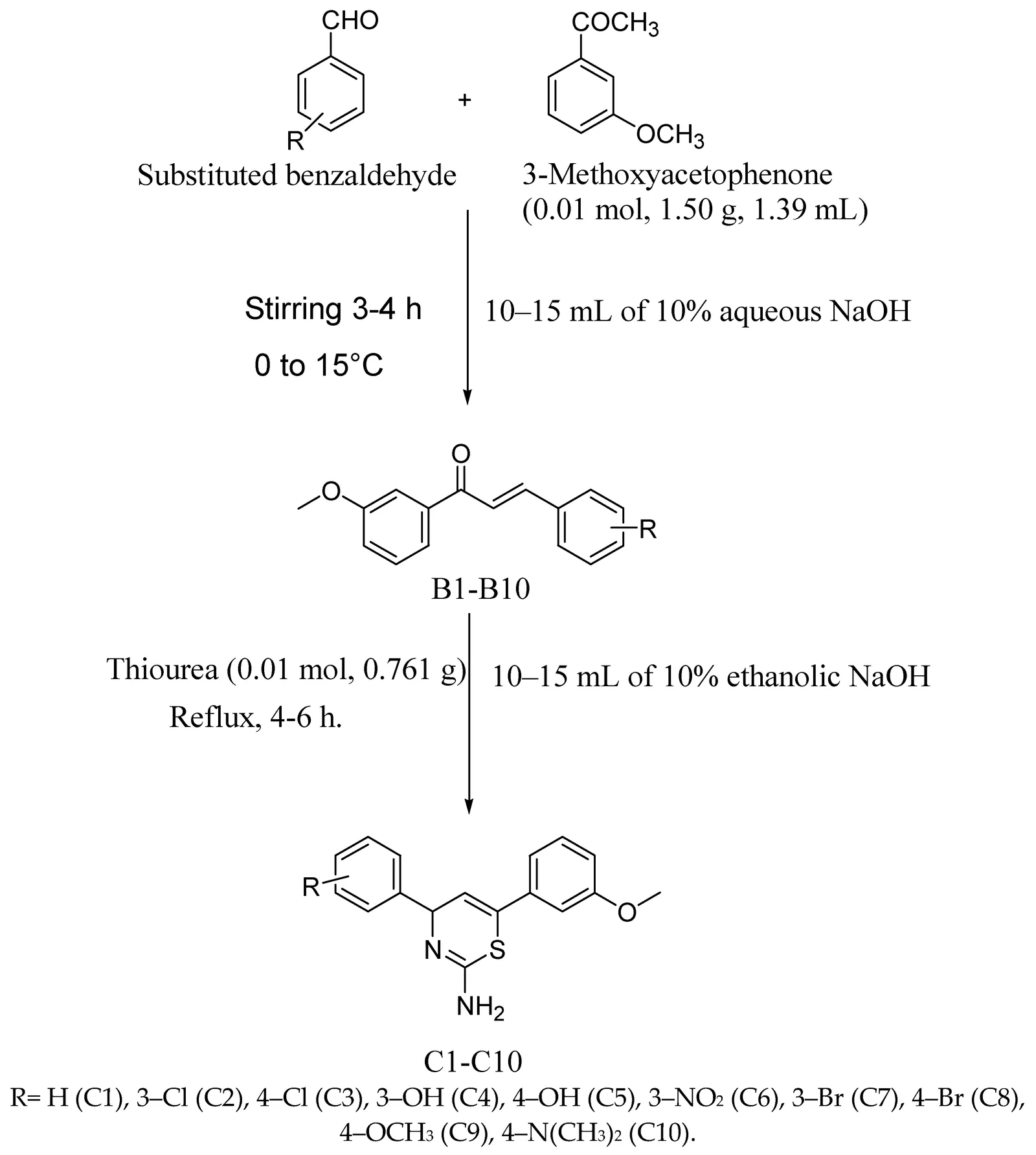
Synthetic route for the preparation of novel thiazine derivatives **(C1–C10)** from chalcone intermediates.

The synthetic route for the preparation of the chalcone intermediates (B1–B10) is depicted in Scheme 1. The key intermediate chalcones were synthesized by stirring substituted benzaldehyde with 3-methoxyacetophenone in absolute alcohol in the presence of 10% aqueous NaOH solution. The resulting chalcone intermediates were subsequently subjected to cyclization with thiourea to afford the corresponding thiazine derivatives (C1–C10), as illustrated in Scheme 2. The structures of the synthesized thiazine derivatives are presented in Table 1.

The purity of all synthesized derivatives and intermediates was assessed using TLC. The M.P. of all thiazine derivatives was checked by open capillary by electric melting point apparatus and found in the range of 109–155 °C (Table 2). Maximum yield was presented by the 4-chloro group-containing compound at 67%. All the thiazine derivatives presented the R_f_ value in the range of 0.45–0.79.

FT-IR, ^1^H-NMR, ^13^C-NMR, and mass spectrometry were applied to confirm the structures of synthesized derivatives. The FT-IR spectra of synthesized derivatives were characterized by the attendance of a strong band at 3237–3417 cm^−1^ for the N-H group, which is calculated as a strong confirmation for the amine group arrangement. The C=N band was present in the range of 1636–1599 cm^−1^. The NH_2_ protons attached to the thiazine ring exhibited a peak at 6.50–6.52 ppm. The structures of all compounds were additionally recognized by mass spectrometry and demonstrated the equivalent M + 1 peaks established to exact molecular formulas.

### 2.2. ADME and Toxicity Studies

Because of their poor pharmacokinetics and toxicity issues, many drugs fail during the drug development process. Early in this process’ pipeline, problems that arise during drug development could be resolved. The initial step in this pipeline procedure is to use in silico ADMET methodologies to analyze this problem of new chemical compounds. By using these techniques, time is saved on a lead chemical that could be harmful or that the body would convert into an inactive version that could not pass through membranes [20]. In the present study, ADME properties were predicted using the SwissADME web-based platform (https://www.swissadme.ch/ (accessed on 24 August 2026)), while toxicity-related parameters were predicted using the ProTox 3.0 web server (https://tox.charite.de/protox3/ (accessed on 24 August 2026)). The corresponding computational platforms, access information, and parameter sources are specified to facilitate reproducibility. The results of the ADME and toxicity evaluation were presented in Table 3.

The synthesized compounds exhibited molecular weights below 500 g/mol, with LogP values ranging from 3.545 to 4.601, and showed favorable predicted human intestinal absorption ranging from 88.678% to 93.494%. Compounds C4, C5, and C6 were predicted to be P-glycoprotein substrates, whereas the remaining compounds were predicted to be non-substrates. For P-glycoprotein I inhibition, C1, C2, C3, C6, C7, C8, C9, and C10 were predicted as inhibitors, whereas C4 and C5 were predicted as non-inhibitors. None of the synthesized compounds were predicted to inhibit P-glycoprotein II.

The predicted BBB permeability values ranged from −0.321 to 0.190, while CNS permeability values ranged from −1.973 to −1.315. Toxicity predictions indicated that compounds C1-C8 and C10 were Ames-positive, whereas C9 was predicted to be Ames-negative. None of the compounds were predicted to inhibit hERG I, while most compounds were predicted to inhibit hERG II; C6 and C9 were predicted as non-inhibitors for this endpoint. Hepatotoxicity was predicted for C1, C2, C3, and C6, whereas C4, C5, C7, C8, C9, and C10 were predicted to be non-hepatotoxic. Only compound C2 showed predicted skin sensitization.

Overall, the synthesized derivatives showed generally favorable predicted absorption and physicochemical characteristics; however, the predicted Ames mutagenicity, hERG II inhibition, and hepatotoxicity observed for some compounds indicate potential safety liabilities. Therefore, these computational predictions require further experimental validation before the therapeutic potential of these compounds can be established. The detailed ADMET parameters are provided in the Supplementary Information.

### 2.3. Molecular Docking Results

**Figure 1:**
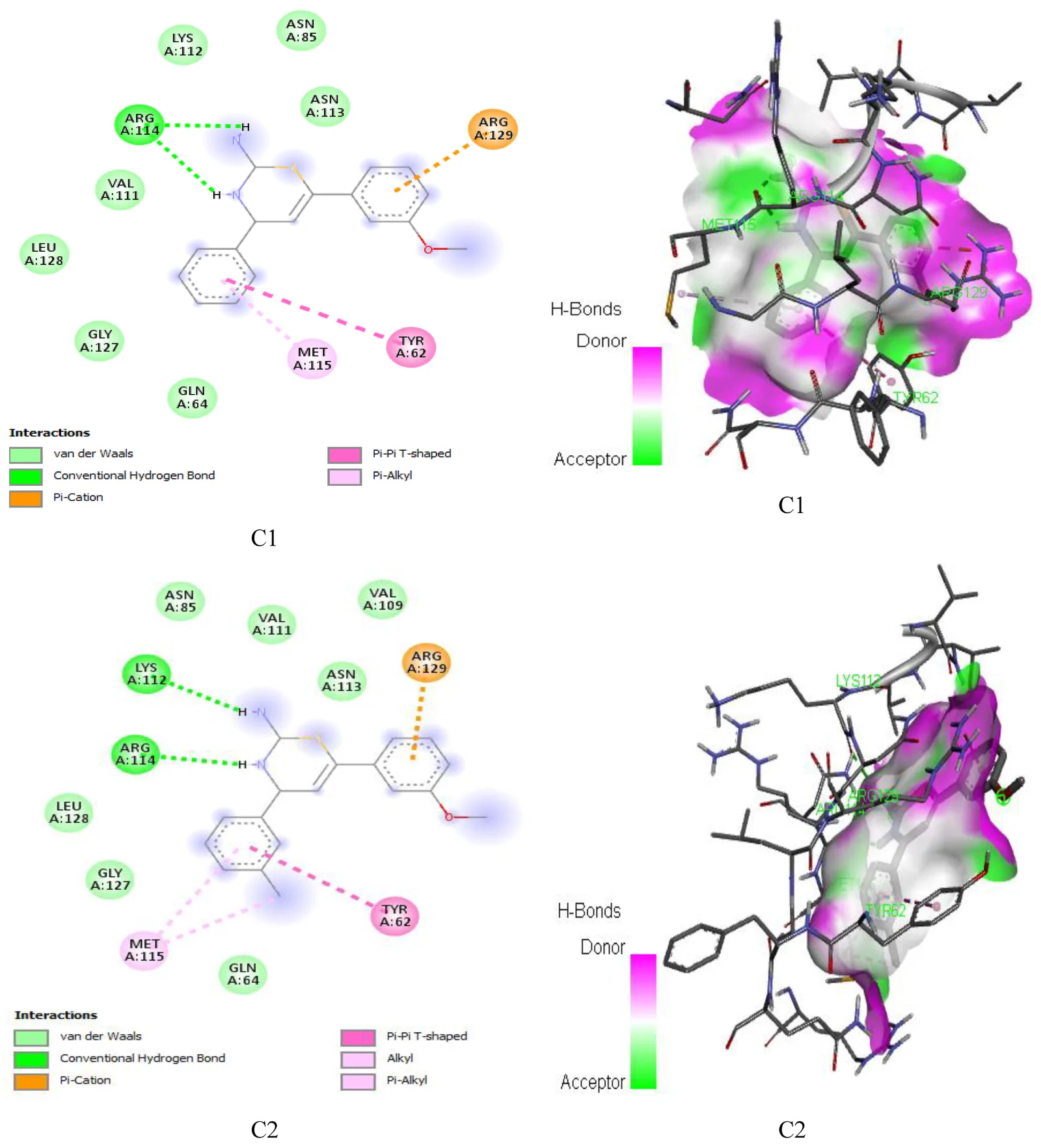

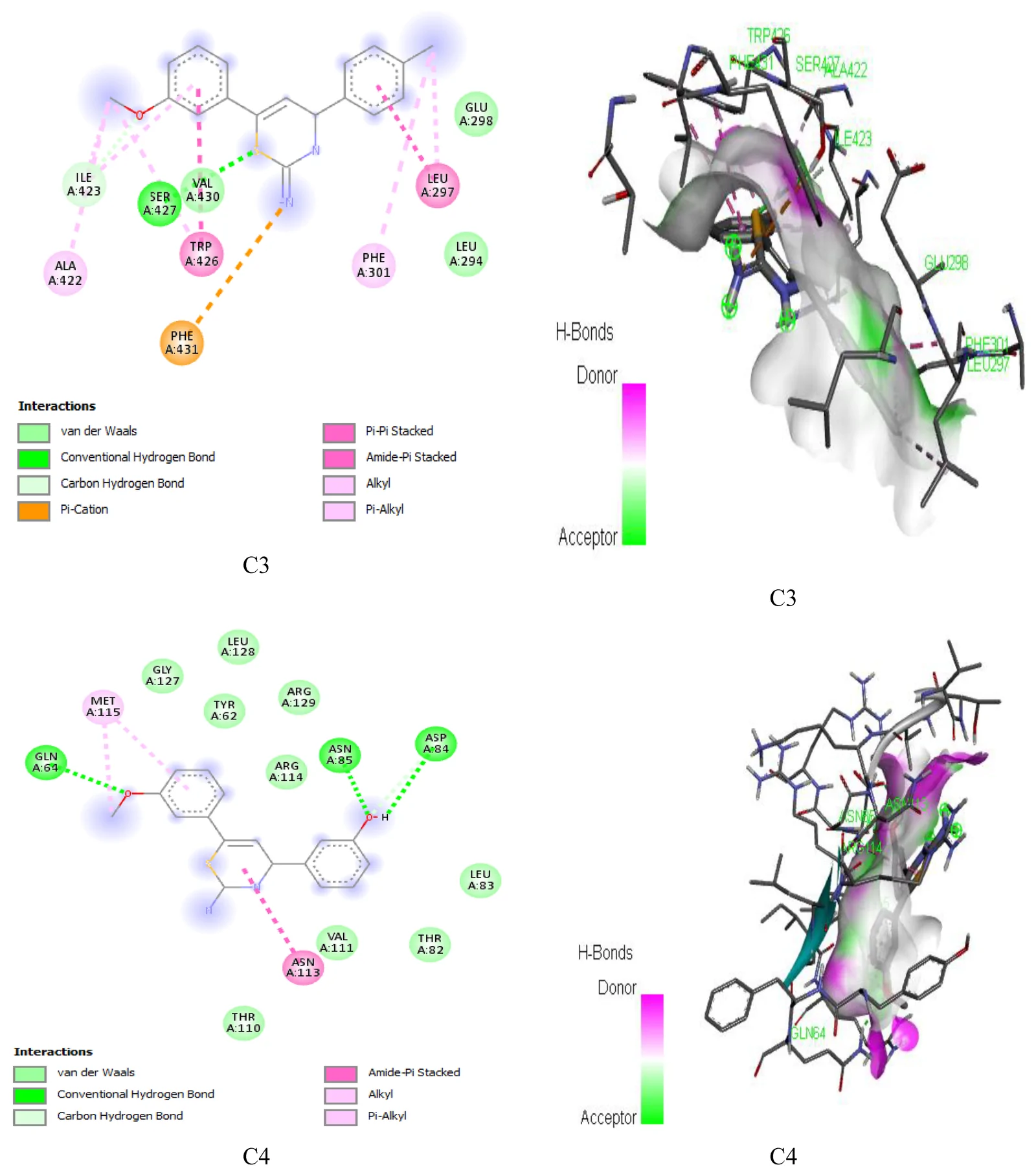

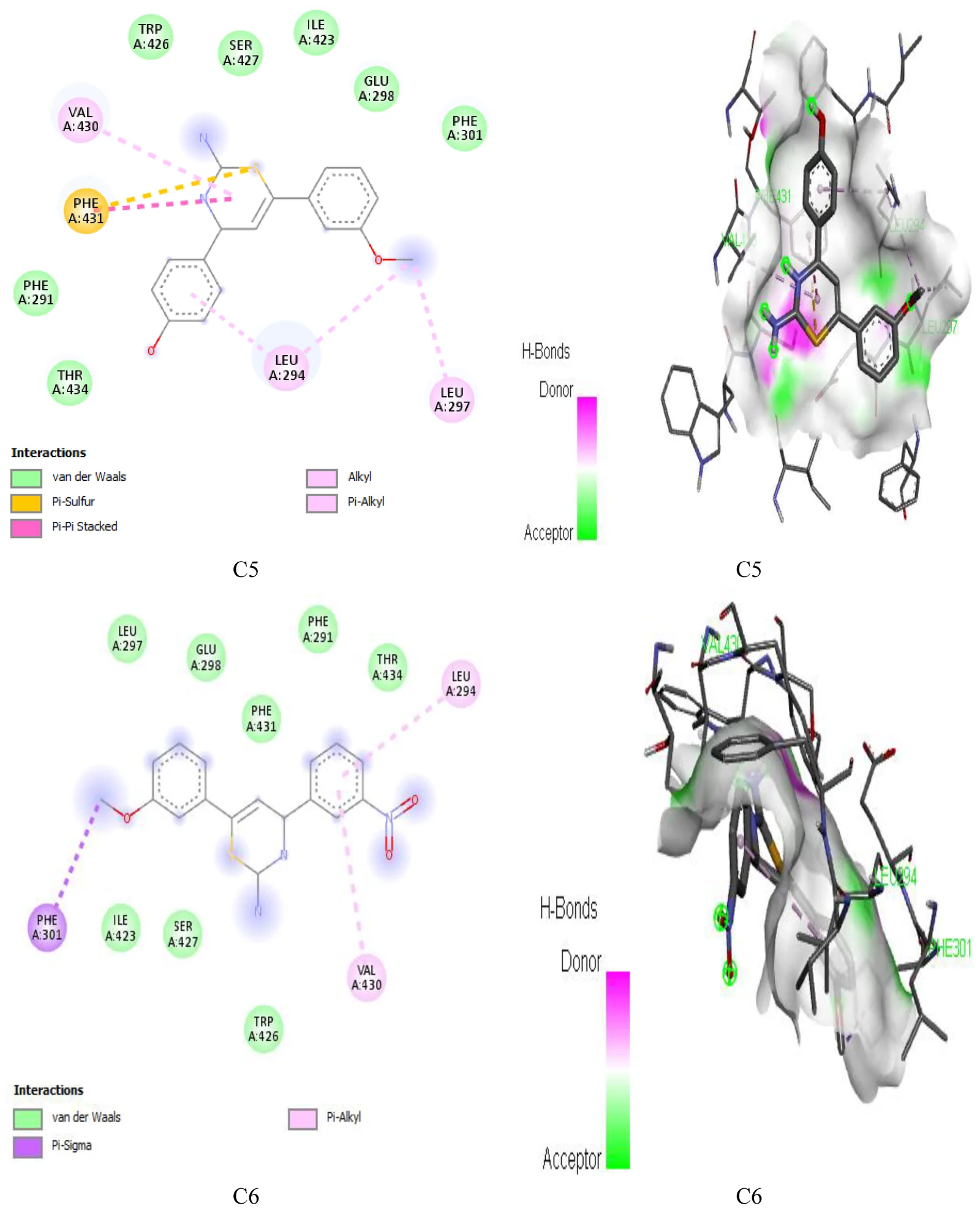

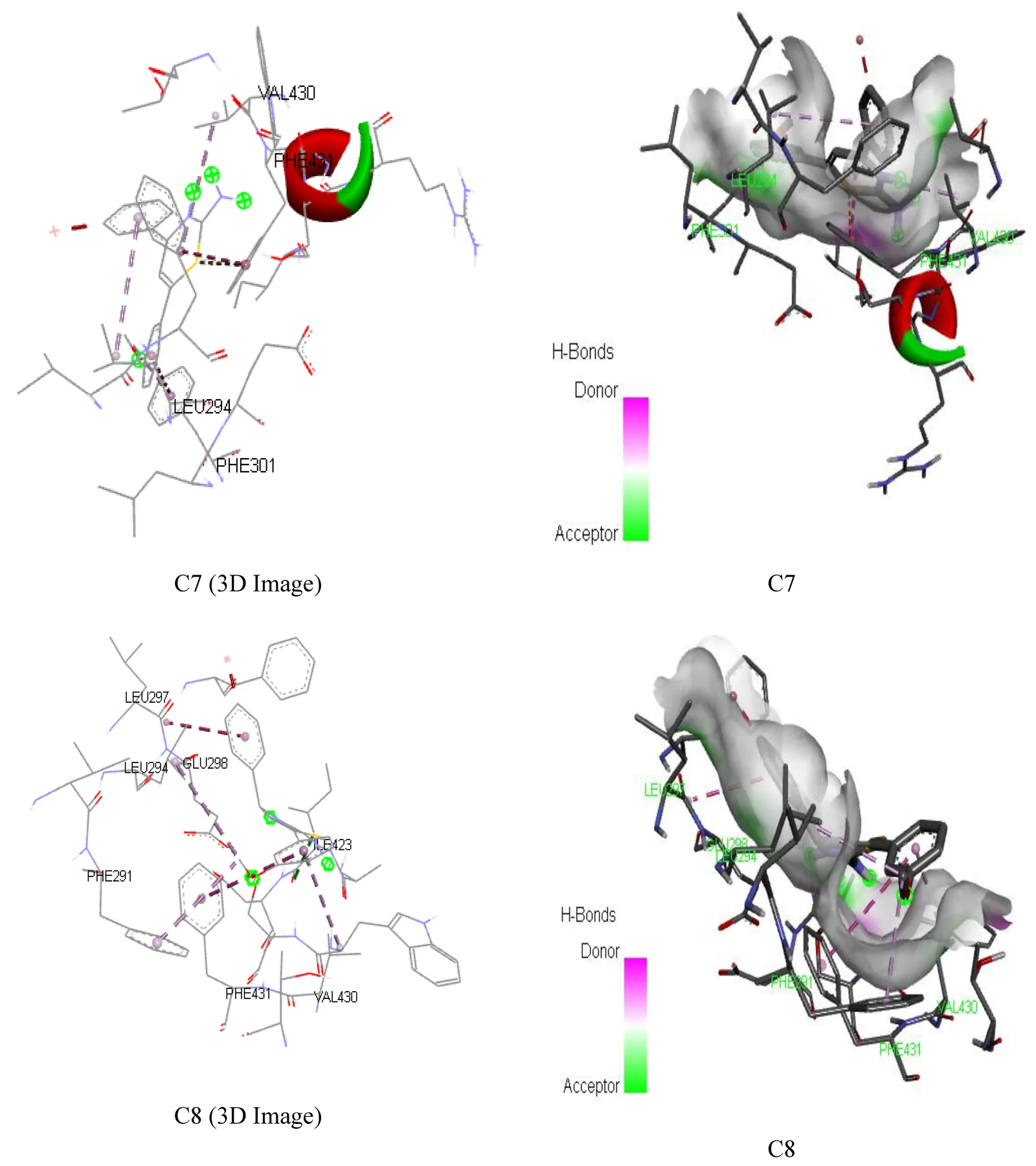

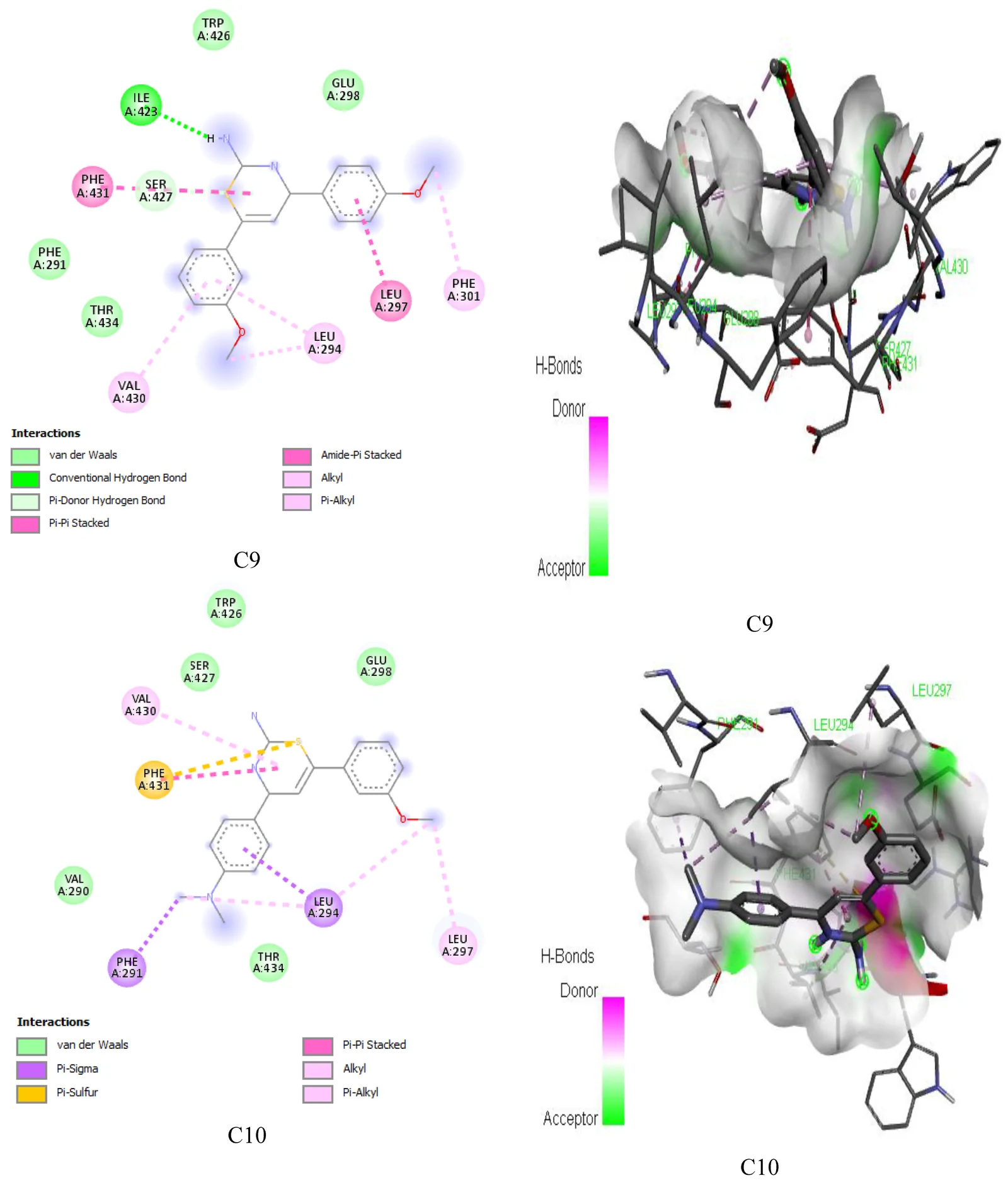
2D and 3D molecular docking interactions of thiazine derivatives (C1–C10) with the tar-get GABAA receptor.

Ten thiazine derivatives (C1–C10) and a standard drug were docked with the GABA_A_ receptor to predict the binding energy by AutoDock Vina 4.2. The binding energy of all thiazine derivatives is shown in Table 4. The standard drug alprazolam exhibited a predicted docking score of −8.1 kcal/mol. The predicted docking scores of all the synthesized thiazine derivatives based on chalcones ranged from −9.1 to −6.8 kcal/mol. Compound C8 exhibited the least favorable predicted docking score (−6.8 kcal/mol), whereas compounds C3 and C6 exhibited the most favorable predicted docking scores (−9.0 and −9.1 kcal/mol, respectively). The test compounds were found to make hydrogen bond interactions with backbone Arg114, Lys112, Ser427, Asp84, Asn85, Phe291, and Ile423, whereas pi-Alkyl interactions were observed for Met115, Ala422, Ile423, Leu294, Leu297, and Val430, but Leu294 and Val430 showed similarities with the standard drug alprazolam. 2D molecular docking interactions of thiazine derivatives and the standard drug alprazolam with the target, the GABA_A_ receptor, are presented in Figure 1.

### 2.4. Structure–Activity Relationship (SAR) of Thiazine Derivative

The structure–activity relationship of the synthesized thiazine derivatives was evaluated by considering the predicted docking scores, experimentally determined GABA-AT inhibitory activity and anxiolytic-like activity in the elevated plus maze (EPM) model. The observed activity was influenced not only by the electron-withdrawing or electron-donating nature of the substituent but also by its position, steric characteristics, lipophilicity, and hydrogen-bonding potential. Among the synthesized compounds, C3 bearing a para-chloro substituent showed a favorable predicted docking score (−9.0 kcal/mol) and good GABA-AT inhibitory activity (IC_50_ = 14.61 ± 1.72 µM). The chloro substituent may contribute to hydrophobic interactions and increased lipophilic character, which may favor molecular recognition.

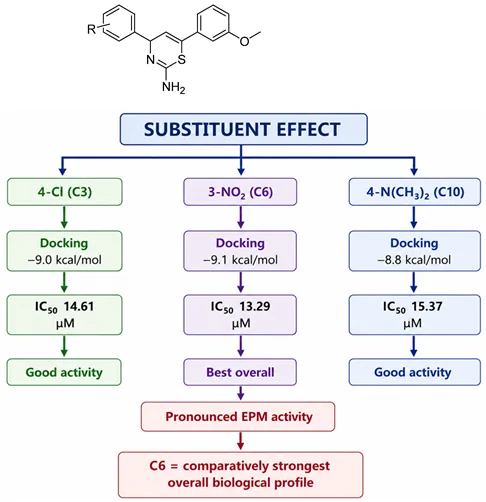



Compound C6, containing a meta-nitro substituent, exhibited the most favorable predicted docking score (−9.1 kcal/mol), strong GABA-AT inhibitory activity (IC_50_ = 13.29 ± 1.62 µM), and the most pronounced anxiolytic-like activity in the EPM model. The nitro group provides strong electron-withdrawing character and additional hydrogen-bond-accepting capacity, which may contribute to favorable molecular interactions. In contrast, C10 containing a para-dimethylamino substituent also showed a favorable docking score (−8.8 kcal/mol), together with good GABA-AT inhibitory activity (IC_50_ = 15.37 ± 1.90 µM) and anxiolytic-like activity. This observation indicates that both electron-withdrawing and electron-donating substituents can support biological activity when appropriately positioned on the aromatic ring.

The comparison of docking, GABA-AT inhibition, and EPM results suggests that the three datasets do not follow a strictly linear relationship. Although C3, C6, and C10 showed comparatively favorable overall profiles, docking scores alone cannot be considered direct predictors of experimental enzyme inhibition or behavioral efficacy. Rather, the docking results provide supportive information regarding possible molecular interactions, while the GABA-AT IC_50_ and EPM findings provide experimental evidence of enzyme inhibition and anxiolytic-like activity, respectively. Therefore, the combined results suggest that substituent position, steric effects, lipophilicity, hydrogen-bonding potential, and electronic characteristics collectively contribute to the observed SAR.

### 2.5. Acute Toxicity Results

All synthesized compounds C1–C10 underwent preliminary toxicity testing on animals in accordance with OECD guidelines. Every synthetic derivative was given orally. The animals always had free access to mineral water and were given an ad libitum regular feed diet. Every animal was closely observed for 4 h the first time and then every day for 14 d after that. The parameters of toxicity, such as ocular assessment of mortality, any changes in physical appearance, skin irritation, hyperactivity, eye twitching, catalepsy, convulsions, and edema, were all carefully observed. The effects of skin irritation, CNS stimulation, and CNS depression were observed. The parameters of CNS depression were ataxia, catatonia, and sedation. The observation was recorded for 0.5 h, 4 h, 24 h, 48 h, 7 d, and 14 d. The observation showed that none of the specified parameters were present. No observable signs of acute toxicity were detected under the experimental conditions and doses evaluated. Animals showing no signs or symptoms of acute toxicity were recorded. No observable signs of acute toxicity were detected for the tested compounds at the evaluated dose of 50 mg/kg b.w. under the experimental conditions. Based on these observations, 5 mg/kg b.w. (one-tenth of the evaluated dose) was selected for the subsequent pharmacological evaluation.

### 2.6. Anxiolytic Activity Results

The anxiolytic-like activity of the synthesized thiazine derivatives was evaluated using the elevated plus maze (EPM) model, and the results are presented in Table 5. Among the tested compounds, compound C6 exhibited the most pronounced anxiolytic-like effect under the experimental conditions employed. The behavioral parameters evaluated in the EPM included time spent in the open arms, number of open-arm entries, and percentage of open-arm entries. The results were expressed as mean ± SEM (n = 6 animals/group).

### 2.7. Statistical Analysis

Data were expressed as mean ± SEM. Statistical analyses were performed using GraphPad Prism version 9.0 (Graph Pad Software, San Diego, CA, USA). Differences among groups were analyzed using one-way analysis of variance (ANOVA), followed by Dunnett’s multiple-comparisons test. A value of *p* < 0.05 was considered statistically significant. The sample size (n = 6) represents the number of animals per group. IC_50_ values were determined by nonlinear regression analysis.

### 2.8. In Vitro GABA-AT Enzyme Assay Results

IC_50_ values of synthesized derivatives were assessed by a commercially available kit to acquire impending GABA-AT inhibition action in the samples of brain tissue. The results are presented in Table 5. Among the synthesized compounds, C6 exhibited the strongest GABA-AT inhibitory activity with an IC_50_ value of 13.29 ± 1.62 µM under the experimental conditions employed.

## 3. Materials and Methods

### 3.1. Chemicals, Reagents and Instruments

All chemicals and reagents were used without additional purification after being acquired from commercial vendors (CDH Pvt. Ltd., New Delhi, India; Loba Chemie Pvt. Ltd., Mumbai, India). Melting points (M.P.) are uncorrected and were measured using an electric melting point instrument and the open capillary tube method. Thin layer chromatography (TLC) using silica gel G plates was used to track the reaction’s progress, and an iodine chamber was used to see the spots. TLC was carried out using hexane and Ethyl acetate (7:3) as the mobile phase. The FT-IR spectrophotometer (8400SS, Shimadzu, Kyoto, Japan) was used to perform FT-IR spectroscopy. The ^1^H NMR and ^13^C NMR spectra were recorded in CDCl_3_, and the chemical shifts (δ, ppm) were referenced to the residual solvent peak of CDCl_3_ (δH = 7.26 ppm and δC = 77.16 ppm).

### 3.2. Synthesis

#### 3.2.1. Synthesis of Chalcones (**B1**–**B10**)

50 mL of absolute alcohol were used to dissolve 0.01 mol of substituted benzaldehyde and 0.01 mol of 3-methoxyacetophenone (0.01 mol, 1.50 g, 1.39 mL). After that, 10% aqueous NaOH solution (1 mmol) was added at 0 to 15 °C, the reaction mixture was agitated for 3–4 h. TLC monitored the progress of the reaction. The precipitate was then obtained by adding 50 mL of water. After that, the solid precipitate was filtered, cleaned with water, and allowed to dry. Absolute alcohol was utilized to recrystallize the dry crude product.

#### 3.2.2. Thiazine Derivatives Synthesized from Chalcone Intermediates (**B1**–**B10**)

In an ethanolic NaOH solution, 0.01 mol of chalcones and thiourea (0.01 mol, 0.761 g) were dissolved and agitated for 2 h at room temperature. The reaction mixture was then refluxed for 4–6 h. The reaction mixture was cooled, concentrated, and then added to ice-cold water while being constantly stirred for 1 h. To get the precipitate, the reaction mixture was refrigerated for the whole night. To obtain the pure product, the precipitate was filtered, washed with water, and recrystallized from absolute alcohol [21].

**Scheme 3:**
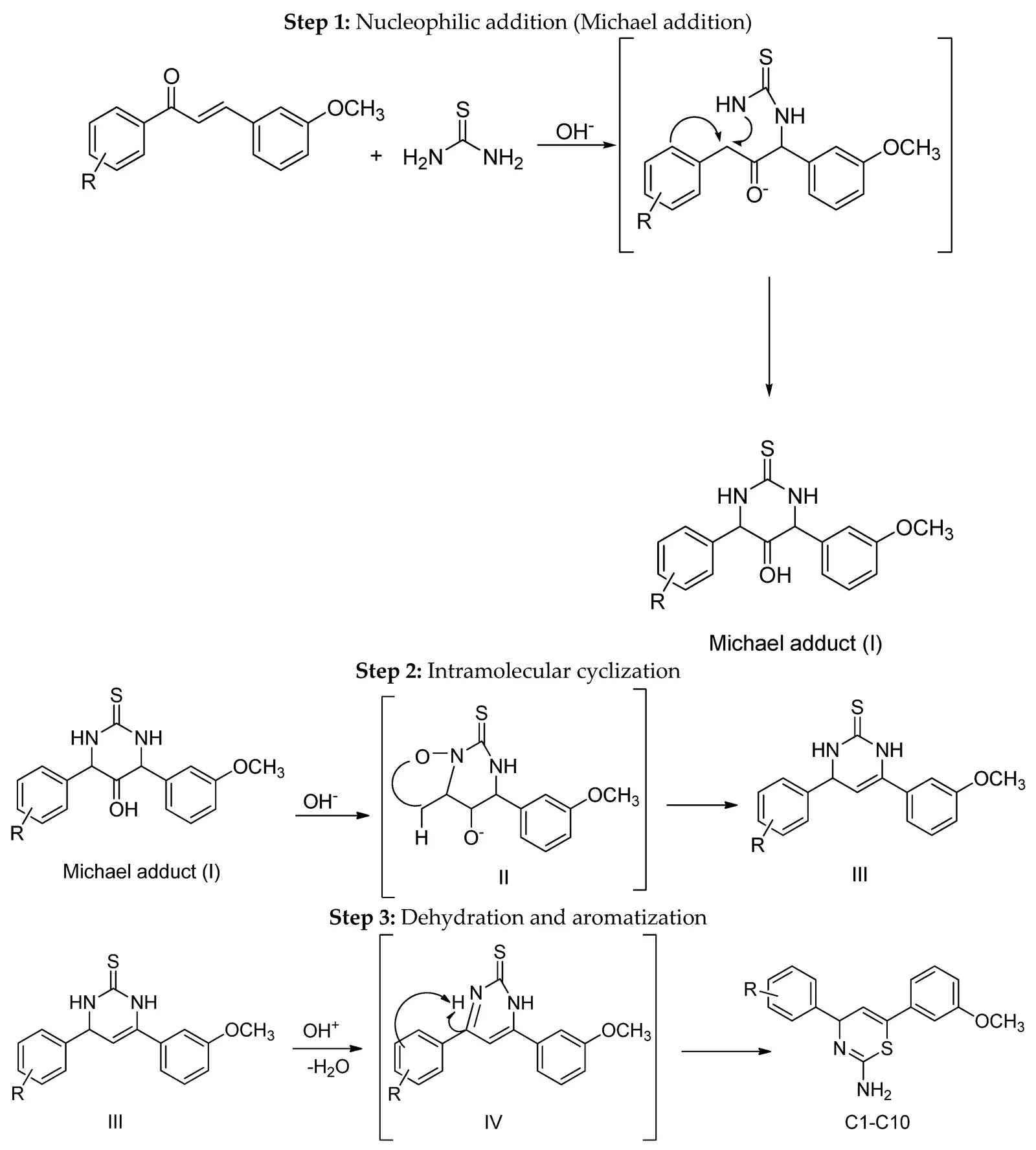
Proposed mechanism for the formation of thiazine derivatives (**C1–C10)**.

The synthesized thiazine derivatives were obtained in moderate yields (42–67%). The relatively modest yields may be attributed to the cyclization efficiency of the chalcone intermediates with thiourea, as well as the electronic and steric effects of different substituents on the aromatic ring. Electron-withdrawing and electron-donating substituents can influence the reaction rate and product formation, leading to variations in the isolated yields. Despite the moderate yields, the products were obtained in sufficient purity and quantity for complete characterization and subsequent biological evaluation Scheme 3.

##### 6-(3-Methoxyphenyl)-4-phenyl-4H-[1,3]thiazin-2-ylamine (**C1**)

White solid, yield: 52.80%, m.p.: 112–114 °C; Rf = 0.79 (Hexane: Ethyl acetate, 7:3); FT-IR (KBr, cm^−1^): 3015 (C-H Str., Ar.), 3473 (N-H str.), 1636 (C=N Str.), 1030 (C-O Str.), 1464 (CH=CH); ^1^H-NMR (500 MHz, CDCl_3_) δ: 7.43 (*d*, 2H, *J* =1.0 Hz, Ar.), 7.41 (*d*, 2H, *J* =1.5 Hz, Ar.), 7.29 (*t*, 2H, *J* = 0.5 Hz, Ar.), 7.26 (*d*, 2H, *J* = 2.0 Hz, Ar.), 6.78 (*t*, 1H, *J* = 1.5 Hz, Ar.), 6.50 (*d*, 2H, NH_2_), 6.02 (*s*, 1H, CH), 5.58 (*d*, 1H, CH, thiazine), 3.78 (*s*, 3H,-OCH_3_); ^13^C-NMR (125 MHz, CDCl_3_) δ: 129.43, 129.03, 127.17, 126.01, 119.80, 118.57, 115.04, 113.55, 68.35, 55.44; Mass: *m*/*z*: (M + 1) 297.01; Anal. Calcd: C, 68.89; H, 5.44; O, 5.40; N, 9.45. Found: C, 68.73; H, 5.39; O, 5.36; N, 9.40.

##### 4-(3-Chloro-phenyl)-6-(3-methoxyphenyl)-4H-[1,3] thiazin-2-ylamine (**C2**)

White solid, yield: 54.66%, m.p.: 109–111 °C; Rf = 0.45 (Hexane: Ethyl acetate, 7:3); FT-IR (KBr, cm^−1^): 3077 (C-H Str., Ar.), 3472 (N-H Str.), 1619 (C=N Str.), 1180 (C-O Str.), 2861 (O-CH_3_ Str.), 1457 (CH=CH), 604 (C-Cl Str.); ^1^H-NMR (500 MHz, CDCl_3_) δ: 7.52 (*t*, 1H, *J* = 2.5 Hz, Ar.), 7.46 (*d*, 1H, *J* = 8.0 Hz, Ar.), 7.39 (*d*, 2H, *J* =1.0 Hz, Ar.), 7.37 (*d*, 1H, *J* = 1.5 Hz, Ar.), 7.31 (*s*, 1H, Ar.), 7.10 (*t*, 1H, *J* = 2.0 Hz, Ar.), 6.88 (*s*, 1H, Ar.), 6.50 (*s*, 2H, NH_2_), 5.89 (*d*, 1H, CH), 5.49 (*d*, CH, thiazine), 3.78 (*s*, 3H,-OCH_3_); ^13^C-NMR (100 MHz, CDCl_3_) δ:130.06, 129.43, 128.12, 127.58, 124.89, 119.80, 118.42, 115.04, 113.55, 68.63, 55.44; Mass: *m*/*z*: (M + 1) 331.00; Anal. Calcd: C, 61.72; H, 4.57; O, 4.84; N, 8.47. Found: C, 61.65; H, 4.50; O, 4.78; N, 8.41.

##### 4-(4-Chloro-phenyl)-6-(3-methoxyphenyl)-4H-[1, 3] thiazin-2-ylamine (**C3**)

White solid, yield: 67.20%, m.p.: 119–121 °C; Rf = 0.65 (Hexane: Ethyl acetate, 7:3); FT-IR (KBr, cm^−1^): 2935 (C-H Str., Ar.), 3414 (N-H Str.), 1636 (C=N Str.), 1090 (C-O Str.), 2836 (O-CH_3_ Str.), 622 (C-Cl Str.), 1488 (CH=CH); ^1^H-NMR (500 MHz, CDCl_3_) δ: 7.43 (*d*, 2H, *J* = 1.0 Hz, Ar.), 7.39 (*d*, 2H, *J* = 1.5 Hz, Ar.), 7.31 (*d*, 1H, *J* = 2.0 Hz, Ar.), 7.29 (*d*, 1H, *J* = 2.0 Hz, Ar.), 7.10 (*s*, 1H, Ar.); 6.90 (*t*, 1H, *J* = 1.0 Hz, Ar.), 6.50 (*s*, 2H, NH_2_), 6.03 (*d*, 1H, CH), 5.56 (*d*, CH, thiazine), 3.78 (s, 3H,-OCH3), ^13^C-NMR (125 MHz, CDCl_3_) δ: 129.43, 129.23, 127.36, 119.80, 118.65, 115.04, 113.55, 68.12, 55.44; Mass: *m*/*z*: (M + 1) 331.01; Anal. Calcd: C, 61.72; H, 4.57; O, 4.84; N, 8.47. Found: C, 61.68; H, 4.49; O, 4.80; N, 8.40.

##### 3-[2-Amino-6-(3-methoxyphenyl)-4H-[1, 3] thiazin-4-yl]-phenol (**C4**)

Dark brown solid, yield: 56.62%, m.p.: 122–124 °C; Rf = 0.48 (Hexane: Ethyl acetate, 7:3); FT-IR (KBr, cm^−1^): 3272 (Ar-OH Str.), 3092 (C-H Str., Ar.), 3416 (N-H Str.), 1599 (C=N Str.), 1003 (C-O Str.), 2862 (O-CH_3_ Str.), 1494 (CH=CH); ^1^H-NMR (500 MHz, CDCl_3_) δ: 7.43 (*d*, 1H, *J* = 1.0 Hz, Ar.), 7.31 (*d*, 1H, *J* = 5.5 Hz, Ar.), 7.28 (*d*, 1H, *J* = 1.5 Hz, Ar.), 7.19 (*d*, 1H, *J* = 3.5 Hz, Ar.), 7.10 (*t*, 1H, *J* = 2.0 Hz, Ar.), 6.93 (*s*, 1H, Ar.), 6.88 (*t*, 1H, *J* = 1.0 Hz, Ar.), 6.74 (*s*, 1H, *J* = 1, Ar.), 6.50 (*s*, 2H, NH_2_), 5.89 (*d*, 1H, CH), 5.44 (*d*, CH, thiazine), 3.78 (*s*, 3H-OCH_3_), 8.14 (s,1H OH); ^13^C-NMR (125 MHz, CDCl_3_) δ:129.83, 129.43, 119.80, 118.46, 118.26, 116.02, 113.61, 113.55, 68.70, 55.44; Mass: *m*/*z*: (M + 1) 313.03; Anal. Calcd: C, 65.36; H, 5.16; O, 10.24; N, 8.97. Found: C, 65.28; H, 5.12; O, 10.18; N, 8.90.

##### 4-[2-Amino-6-(3-methoxyphenyl)-4H-[1, 3] thiazin-4-yl]-phenol (**C5**)

Brown solid, yield: 44.12%, m.p.: 110–112 °C; Rf = 0.56 (Hexane: Ethyl acetate, 7:3); FT-IR (KBr, cm^−1^): 3235 (Ar-OH Str.), 3072 (C-H Str, Ar.), 3417 (N-H Str.), 1636 (C=N, Str.), 1025 (C-O Str.), 1458 (CH=CH); ^1^H-NMR (500 MHz, CDCl_3_) δ: 7.43 (t, 1H, *J* = 1.0 Hz, Ar.), 7.31 (*d*, 3H, *J* = 3.0 Hz, Ar.), 7.10 (*d*, 1H, *J* = 2.0 Hz, Ar.), 6.90 (*d*, 1H, *J* = 1.0 Hz, Ar.), 6.79 (*d*, 2H, *J* = 2.0 Hz, Ar.), 6.50 (*s*, 2H, NH_2_), 5.87 (*d*, 1H, CH), 5.57 (*d*, CH, thiazine), 3.78 (*s*, 3H,-OCH_3_), 7.86 (*s*,1H, OH);^13^C-NMR (125 MHz, CDCl_3_) δ: 129.43, 127.40, 119.80, 118.32, 115.99, 115.55, 155.04, 113.55, 68.53, 55.44; Mass: *m*/*z*: (M + 1) 313.08; Anal. Calcd: C, 65.36; H, 5.16; O, 10.24; N, 8.97. Found: C, 65.25; H, 5.14; O, 10.20; N, 8.89.

##### 6-(3-Methoxyphenyl)-4-(3-nitro-phenyl)-4H-[1, 3] thiazin-2-ylamine (**C6**)

Black solid, yield: 42.56%, m.p.: 126–128 °C; Rf = 0.66 (Hexane: Ethyl acetate, 7:3); FT-IR (KBr, cm^−1^): 3012 (C-H Str. Ar.), 3415 (N-H Str.), 1604 (C=N Str.), 1109 (C-O Str.), 2864 (O-CH_3_ Str.), 1509 (N-O Str.), 1604 (CH=CH); ^1^H-NMR (500 MHz, CDCl_3_) δ: 8.31 (*d*, 1H, *J* = 0.5 Hz, Ar.), 8.10 (*s*, 1H, Ar.), 7.72 (*d*, 1H, *J* = 1.5 Hz, Ar.), 7.57 (*t*, 1H, *J* = 8.0 Hz, Ar.), 7.42 (*t*, 1H, *J* = 1.0 Hz, Ar.), 7.31 (*d*, 2H, *J* =8.0 Hz, Ar.), 7.10 (*s*, 1H, Ar.), 6.88 (*d*, 1H, *J* = 1.0 Hz, Ar.), 6.50 (*s*, 2H, NH_2_), 5.89 (*d*, 1H, CH), 5.71 (*d*, CH, thiazine), 3.78 (*s*, 3H,-OCH_3_); ^13^C-NMR (125 MHz, CDCl_3_) δ:131.88, 130.39, 129.43, 123.08, 122.21, 119.80, 118.66, 115.04, 113.55, 68.28, 55.44; Mass: *m*/*z*: (M + 1) 342.08; Anal. Calcd: C, 59.81; H, 4.43; O, 14.06; N, 12.31. Found: C, 59.75; H, 4.39; O, 13.99; N, 12.28.

##### 4-(3-Bromo-phenyl)-6-(3-methoxy-phenyl)-4H-[1,3]thiazin-2-ylamine (**C7**)

Dark black solid, yield: 60.62%, m.p.: 145–146 °C; Rf = 0.75 (Hexane: Ethyl acetate, 7:3); FT-IR (KBr, cm^−1^): 3036 (C-H Str., Ar.), 3470 (N-H Str.), 1618 (C=N Str.), 1233 (C-O Str.), 2875 (O-CH_3_ Str.), 1500 (CH=CH), 598 (C-Br Str.); ^1^H-NMR (500 MHz, CDCl_3_) δ: 3.78 (*s*,3H,-OCH_3_), 6.50 (*s*, 2H, NH_2_), 5.57 (*d*, CH, thiazine), 6.03 (*d*, 1H, CH), 7.53 (*d*, 2H, *J* =1.5 Hz, Ar.), 7.41 (*d*, 2H, *J* =1.5 Hz, Ar.), 7.36 (*d*, 1H, *J* = 3.5 Hz, Ar.), 7.29 (*d*, 1H, *J* = 2.5 Hz, Ar.), 7.12 (*s*, 1H, Ar.); 6.88 (*t*, 1H, *J* = 1.0 Hz, Ar.); ^13^C-NMR (125 MHz, CDCl_3_) δ:131.99, 129.43, 127.25, 119.80, 118.65, 115.04, 113.55, 68.51, 55.44; Mass: *m*/*z*: (M + 1) 375.003; Anal. Calcd: C, 54.41; H, 4.03; O, 4.26; N, 7.46. Found: C, 54.35; H, 3.97; O, 4.19; N, 7.38.

##### 4-(4-Bromo-phenyl)-6-(3-methoxyphenyl)-4H-[1, 3] thiazin-2-ylamine (**C8**)

Yellow solid, yield: 51.81%, m.p.: 141–143 °C; Rf = 0.78 (Hexane: Ethyl acetate, 7:3); FT-IR (KBr, cm^−1^): 2966 (C-H Str., Ar.), 3416 (N-H Str.), 1618 (C=N Str.), 1089 (C-O Str), 2875 (O-CH_3_ Str.), 1522 (CH=CH), 538 (C-Br Str.); ^1^H-NMR (500 MHz, CDCl_3_) δ: 7.53 (*d*, 2H, *J* = 1.5 Hz, Ar.), 7.45 (*d*, 2H, *J* =1.5 Hz, Ar.), 7.41 (*d*, 1H, *J* = 1.0 Hz, Ar.), 7.31 (*t*, 1H, *J* = 7.5 Hz, Ar.), 7.09 (*d*, 1H, *J* = 2.0 Hz, Ar.), 6.90 (*s*, 1H, Ar.), 6.52 (*s*, 2H, NH_2_), 6.01 (*d*, 1H, CH), 5.56 (*d*, CH, thiazine), 3.78 (*s*, 3H,-OCH_3_); ^13^C-NMR (125 MHz, CDCl_3_) δ:131.99, 129.43, 127.25, 119.80, 118.65, 115.04, 133.55, 68.51, 55.44; Mass: *m*/*z*: (M + 1) 375.00; Anal. Calcd: C, 54.41; H, 4.03; O, 4.26; N, 7.46. Found: C, 54.38; H, 3.95; O, 4.20; N, 7.41.

4-(4-Methoxyphenyl)-6-(3-methoxyphenyl)-4H-[1,3]thiazin-2-ylamine (**C9**)

Brown solid, yield: 58.41%, m.p.: 153–155 °C; Rf = 0.51 (Hexane: Ethyl acetate, 7:3); FT-IR (KBr, cm^−1^): 3046 (C-H Str., Ar.), 3416 (N-H Str.), 1615 (C=N Str.), 1171 (C-O Str.), 2836 (O-CH_3_ Str.),1465 (CH=CH); ^1^H-NMR (500 MHz, CDCl_3_) δ: 7.43 (*d*, 1H, *J* = 1.0 Hz, Ar.), 7.41 (*d*, 1H, *J* = 1.0 Hz, Ar.), 7.33 (*d*, 1H, *J* = 7.5 Hz, Ar.), 7.28 (*d*, 1H, *J* = 11.5 Hz, Ar.), 7.10 (*t*, 1H, *J* = 2.0 Hz, Ar.), 6.94 (*d*, 1H, *J* = 1.0 Hz, Ar.), 6.90 (*d*, 1H, *J* = 1.5 Hz, Ar.), 6.87 (*d*, 1H, *J* = 1.5 Hz, Ar.), 6.50 (*s*, 2H, NH_2_), 5.87 (*d*, 1H, CH), 5.55 (*d*, CH, thiazine), 3.79 (*s*, 6H, -OCH_3_); ^13^C-NMR (100 MHz, CDCl_3_) δ: 129.43, 127.03, 119.80, 118.29, 115.04, 114.23, 113.55, 68.42, 55.44, 55.34; Mass: *m*/*z*: (M + 1) 327.10; Anal. Calcd: C, 66.23; H, 5.56; O, 9.80; N, 8.58. Found: C, 66.16; H, 5.48; O, 9.77; N, 8.41.

4-(4-Dimethylamino-phenyl)-6-(3-methoxyphenyl)-4H-[1, 3] thiazin-2-ylamine (**C10**)

Yellow solid, yield: 52.30%, m.p.: 132–134 °C; Rf = 0.72 (Hexane: Ethyl acetate, 7:3);FT-IR (KBr, cm^−1^): 3067 (C-H Str., Ar.), 3414 (N-H, str.), 1635 (C=N Str.), 1026 (C-O Str.), 2861 (O-CH_3_, Str.), 1453 (CH=CH); ^1^H-NMR (500 MHz, CDCl_3_) δ: 7.42 (*d*, 1H, *J* = 1.5 Hz, Ar.), 7.31 (*d*, 1H, *J* = 3.0 Hz, Ar.), 7.28 (*d*, 1H, *J* = 1.5 Hz, Ar.), 7.27 (*d*, 1H, *J* = 2.0 Hz, Ar.), 7.10 (*t*, 1H, *J* = 2.0 Hz, Ar.), 6.90 (*d*, 1H, *J* = 1.0 Hz, Ar.), 6.89 (*d*, 1H, *J* = 1.0 Hz, Ar.), 6.80 (*d*, 1H, *J* = 2.0 Hz, Ar.), 6.50 (*s*, 2H, NH_2_), 6.01 (*d*, 1H, CH), 5.56 (*d*, CH, thiazine), 3.78 (*s*, 3H,-OCH_3_), 2.92 (*s*, 6H, 2CH_3_); ^13^C-NMR (100 MHz, CDCl_3_) δ:129.43, 126.76, 119.80,118.65, 115.04, 113.55, 112.71, 67.67, 55.44, 40.31; Mass: *m*/*z*: (M + 1) 340.01; Anal. Calcd: C, 67.23; H, 6.24; O, 4.71; N, 12.38. Found: C, 67.19; H, 6.18; O, 4.65; N, 12.31.

### 3.3. ADME and Toxicity Studies

ADME and physicochemical properties of the synthesized compounds were predicted using the SwissADME web-based platform, whereas toxicity-related endpoints were evaluated using the ProTox 3.0 web server. Drug-likeness was assessed with reference to Lipinski’s rule of five, and the predicted pharmacokinetic and toxicity parameters were interpreted together with the results presented in Table 3 [22,23].

### 3.4. Molecular Docking

The Protein Data Bank provided the target proteins for the synthesized compounds (**C1**–**C10**) used in this investigation, which were then docked with the GABA_A_ receptor (PDB ID: 4COF).

The docking protocol was validated by re-docking the native co-crystallized ligand into the designated binding site using the same receptor preparation, grid box, and docking settings applied to the test compounds. The re-docked pose was superimposed on the experimental crystallographic pose, and the root mean square deviation (RMSD) between the two poses was used as the validation criterion [24,25,26]. The RMSD values reported for the synthesized compounds in Table 4 are docking-pose RMSD values and should not be interpreted as the receptor-validation RMSD obtained from re-docking the co-crystallized ligand.

### 3.5. Anxiolytic Activity

The Elevated Plus Maze Test was used to measure the anxiolytic activity. The animal ethics committee’s prior consent was obtained, and all procedures and experiments were carried out in compliance with established guidelines. The Institutional Animal Ethics Committee of IFTM University, which was established in accordance with the Committee for the Purpose of Control and Supervision of Experimental Animals guidelines, gave its approval to the experimental protocols. IAEC/2022/33 is the ethics approval number, and 837/PO/ReBiBt/S/04/CPCSEA is the resolution number. The study conforms to all legal requirements.

### 3.6. Acute Toxicity Study

The synthesized derivatives underwent toxicity screening in accordance with the guideline of OECD 423. Ten groups of female animals were prepared with three animals in each group (n = 3). In the starting phase of acute toxicity evaluation, doses of 5 and 50 mg/kg body weight were administered orally to animals. During a three-hour observation period, their behavioral, neurological, and autonomic responses were evaluated through visual inspection. The observation was carried out for a whole day, with 30 m intervals between each measurement or until the animals died [27,28].

#### 3.6.1. Elevated Plus Maze Method

Swiss albino mice weighing 22–25 g were used (n = 6 animals per group) and maintained under standard housing conditions with food and water available ad libitum at 25 ± 2 °C under a natural light–dark cycle. Fresh solutions of the test compounds were prepared in 1% polyethylene glycol and administered intraperitoneally at 5 mg/kg. Alprazolam (2 mg/kg, i.p.) was used as the reference drug, while the vehicle-treated control group received 1% polyethylene glycol in saline. Treatments were administered 60 min before evaluation in the elevated plus maze. The apparatus consisted of two open arms (15 × 4 cm) and two closed arms (15 × 4 × 11 cm). Each mouse was placed on the central platform facing an open arm and observed for 5 min. The number of open-arm and closed-arm entries and the time spent in the open arms were recorded. The percentage of open-arm entries was calculated as [open-arm entries/(open-arm entries + closed-arm entries)] × 100 [29,30,31].

#### 3.6.2. In Vitro GABA-AT Enzyme Assay

The synthesized derivatives were screened in vitro for GABA-AT inhibitory activity using whole mouse brain tissue and a commercially available assay kit, following the manufacturer’s instructions with minor modifications. The assay is based on sequential GABA-AT-mediated transamination and glutamate dehydrogenase reactions coupled to the reduction in iodonitrotetrazolium (INT) to INT-formazan, which was monitored at 492 nm (epsilon = 18 mM^−1^ cm^−1^). Approximately 50 mg of brain tissue was homogenized in 1 mL of 1× cell lysis solution at 4 °C for 3 min. The supernatant was collected for the enzyme activity assay and stored at −8 °C until analysis [32]. IC50 values were determined by nonlinear regression analysis as described in Section 2.7.

## 4. Conclusions

In this study, a variety of physiochemical techniques were used to characterize the newly synthesized thiazine derivatives (C1–C10). A deeper comprehension of their chemical makeup and their uses resulted from these methods’ significant contributions to our knowledge of their spectroscopic and structural characteristics. Molecular docking studies predicted favorable binding interactions of the synthesized compounds with the GABAA receptor (PDB ID: 4COF). The docking protocol was assessed by re-docking the native co-crystallized ligand and comparing the re-docked pose with its crystallographic pose using RMSD. Among the synthesized compounds, C3 and C6 exhibited the most favorable predicted docking scores (−9.0 and −9.1 kcal/mol, respectively) and favorable hydrogen-bonding interactions with the GABAA receptor.

Moreover, an in silico evaluation of the compounds’ ADMET properties using the SwissADME web-based platform provided valuable information on their drug-likeness and potential for further development. However, the predicted ADMET profiles indicated certain limitations, including Ames positivity, hERG II liability, and hepatotoxicity for some compounds, highlighting the need for further experimental safety evaluation. The synthesized compounds were evaluated for anxiolytic-like activity using the elevated plus maze model. Among the synthesized compounds, C6 exhibited the most pronounced anxiolytic-like activity and showed comparatively favorable GABA-AT inhibitory activity. The comparatively higher activity of C6 suggests that the 3-NO_2_ substitution may contribute to its pharmacological profile; however, additional analogues are required to establish this structure–activity relationship. Overall, C6 may be considered a preliminary lead candidate for further investigation; however, its predicted Ames positivity and hepatotoxicity, together with the need for additional mechanistic and safety studies, should be carefully considered before further lead development.

## Figures and Tables

**Table 1:** Structures of synthesized thiazine derivatives (**C1**–**C10**).

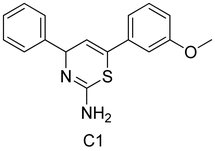	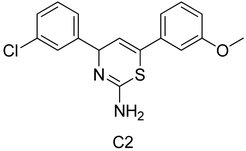
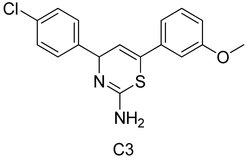	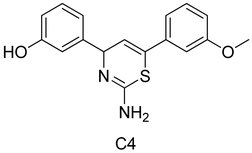
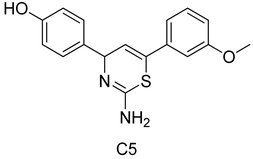	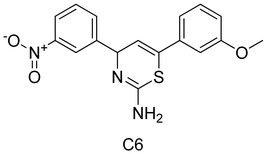
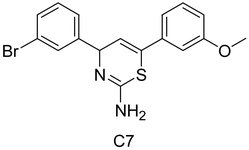	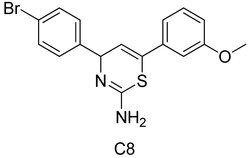
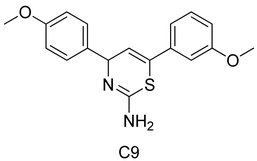	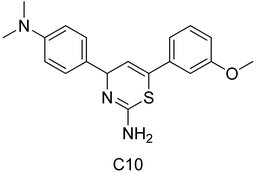

**Table 2:** Physical evaluation parameters of thiazine derivatives (**C1**–**C10**).

S. No.	Compound	R	Melting Point (°C)	% Yield
1	C1	H	112–114	53
2	C2	3-Cl	109–111	55
3	C3	4-Cl	119–121	67
4	C4	3-OH	122–124	57
5	C5	4-OH	110–112	44
6	C6	3-NO_2_	126–128	43
7	C7	3-Br	145–146	61
8	C8	4-Br	141–143	52
9	C9	4-OCH_3_	153–155	58
10	C10	4-N(CH_3_)_2_	132–134	52

R_f_ values were determined by TLC using hexane: ethyl acetate (7:3, *v*/*v*) as the eluent.

**Table 3:** **ADMET and physicochemical properties of synthesized thiazine derivatives (C1–C10).**

Parameter	C1	C2	C3	C4	C5	C6	C7	C8	C9	C10
Molecular Weight (g/mol)	296.395	330.84	330.84	312.394	312.394	341.392	375.291	375.291	326.421	339.464
LogP	3.839	4.492	4.492	3.545	3.545	3.747	4.601	4.601	3.848	3.905
Rotatable Bonds	3	3	3	3	3	4	3	3	4	4
H-bond Acceptors	4	4	4	5	5	6	4	4	5	5
H-bond Donors	1	1	1	2	2	1	1	1	1	1
Surface Area	128.09	138.393	138.393	132.884	132.884	142.743	141.957	141.957	139.568	146.579
Water Solubility	−4.741	−5.539	−5.487	−4.346	−4.264	−5.132	−5.603	−5.603	−5.178	−5.144
Human Intestinal Absorption (%)	91.117	90.281	89.915	90.703	90.292	88.678	89.848	89.848	93.494	91.515
BBB Permeability	0.19	0.141	0.148	−0.073	−0.081	−0.321	0.147	0.147	−0.054	0.143
CNS Permeability	−1.32	−1.315	−1.322	−1.952	−1.961	−1.973	−1.322	−1.322	−1.947	−1.855
CYP3A4 Substrate	Yes	Yes	Yes	Yes	No	Yes	Yes	Yes	Yes	Yes
AMES Toxicity	Yes	Yes	Yes	Yes	Yes	Yes	Yes	Yes	No	Yes
hERG II Inhibitor	Yes	Yes	Yes	Yes	Yes	No	Yes	Yes	No	Yes
Hepatotoxicity	Yes	Yes	Yes	No	No	Yes	No	No	No	No

The detailed ADMET parameters are provided in the Supplementary Information.

**Table 4:** **Results of molecular docking studies of compounds (C1–C10) and alprazolam against the GABA_A_ receptor.**

Compound	Amino Acid Residues and Types of Interactions	Common Residues with Alprazolam	Predicted Docking Score (kcal/mol)	RMSD (Å)
C1	Tyr62: π–π T-shaped; Arg114: Conventional H-bond; Met115: π-Alkyl; Arg129: π-Cation	—	−7.6	1.215
C2	Tyr62: π–π T-shaped; Lys112: Conventional H-bond; Arg114: Conventional H-bond; Met115: π-Alkyl; Arg129: π-Cation	—	−6.9	2.516
C3	Leu297: Amide–π stacked; Phe301: Alkyl; Ala422: π-Alkyl; Ile423: π-Alkyl; Trp426: π–π stacked; Ser427: Carbon H-bond; Phe431: π-Alkyl	—	−9.0	3.020
C4	Gln64: Amide–π stacked; Asp84: Conventional H-bond; Asn85: Conventional H-bond; Asn113: Amide–π stacked; Met115: π-Alkyl	—	−8.2	3.546
C5	Leu294: π-Alkyl; Leu297: π-Alkyl; Val430: Alkyl; Phe431: π-Sulfur	Leu294; Val430; Phe431	−8.7	3.035
C6	Leu294: π-Alkyl; Phe301: π-Sigma; Val430: π-Alkyl	Leu294; Val430	−9.1	1.157
C7	Leu294: π-Alkyl; Phe301: π-Sigma; Val430: π-Alkyl; Phe431: π-Sulfur	Leu294; Val430; Phe431	−7.1	1.798
C8	Phe291: Conventional H-bond; Leu294: π- Alkyl; Leu297: π-Alkyl; Ile423: π-Alkyl; Val430: π-Alkyl; Phe431: π-Sulfur	Leu294; Val430; Phe431	−6.8	2.147
C9	Leu294: π-Alkyl; Leu297: π-Alkyl; Phe301: Amide–π stacked; Ile423: Conventional H-bond; Val430: π-Alkyl; Phe431: π-Sulfur	Leu294; Val430; Phe431	−7.2	3.208
C10	Phe291: π-Sigma; Leu294: π-Sigma; Leu297: π-Alkyl; Val430: π-Alkyl; Phe431: π-Sulfur	Leu294; Val430; Phe431	−8.8	3.889
Alprazolam	Leu294: π-Alkyl; Phe431: π-Alkyl; Val430: Alkyl	—	−8.1	2.751

RMSD, root mean square deviation.

**Table 5:** Results of anxiolytic activity and in vitro GABA-AT inhibitory activity.

Compound	Dose (mg/kg)	Time Spent in Open Arms (s)	Number of Open-Arm Entries	Percentage of Open-Arm Entries (%)	IC_50_ (µM)
C1	5	67.46 ± 1.61	8.12 ± 0.42	47.98	24.59 ± 2.56
C2	5	51.74 ± 3.86	4.16 ± 0.48	34.11	33.32 ± 3.08
C3	5	83.21 ± 1.72 *	10.54 ± 0.74 *	59.39	14.61 ± 1.72
C4	5	68.19 ± 1.93	7.42 ± 0.56 *	52.38	20.53 ± 2.18
C5	5	80.44± 0.71 *	9.50 ± 0.76 *	57.98	17.88 ± 1.58
C6	5	85.48 ± 0.86 *	9.94 ± 0.78 *	62.42	13.29 ± 1.62
C7	5	55.76 ± 2.98	3.81 ± 0.23	38.62	30.55 ± 3.05
C8	5	59.13 ± 1.72	5.68 ± 0.56	48.11	23.76 ± 2.91
C9	5	49.96 ± 3.72	4.12 ± 0.18	32.12	35.78 ± 3.15
C10	5	81.12 ± 1.65 *	10.26 ± 0.66 *	58.26	15.37 ± 1.90
Alprazolam	2	92.82± 2.32	10.11± 0.73	62.46	11.39 ± 1.06
Polyethylene glycol	1%	41.12 ± 3.44	2.82± 092	21.04	-

Data are expressed as mean ± SEM (n = 6 animals/group). Statistical significance was determined using one-way ANOVA followed by Dunnett’s multiple-comparisons test, with the vehicle-treated group as the reference. * *p* < 0.05 vs. vehicle-treated group.

## Data Availability

The original contributions presented in the study are included in the article and Supplementary Material, further inquiries can be directed to the corresponding author.

## Supplementary Materials

The following supporting information can be downloaded at https://www.mdpi.com/article/10.3390/ph19091364/s1. 1H NMR, 13C NMR of C1–C10 compounds.
