# Supplementary material for: Synthesis, Spectroscopic Characterization, Molecular Docking Studies Toward the GABAA Receptor, ADMET Prediction, GABA Aminotransferase Inhibition, and Anxiolytic Evaluation of Novel Thiazine Derivatives
Source: Pharmaceuticals (Basel). 2026 Aug 28;19(9):1364. doi: 10.3390/ph19091364 (PMC13610616; doi:10.3390/ph19091364)
Supplement: Supplementary file 1 [file pharmaceuticals-19-01364-s001.zip › C3 FT-IR.pdf]

C3 FT-IR

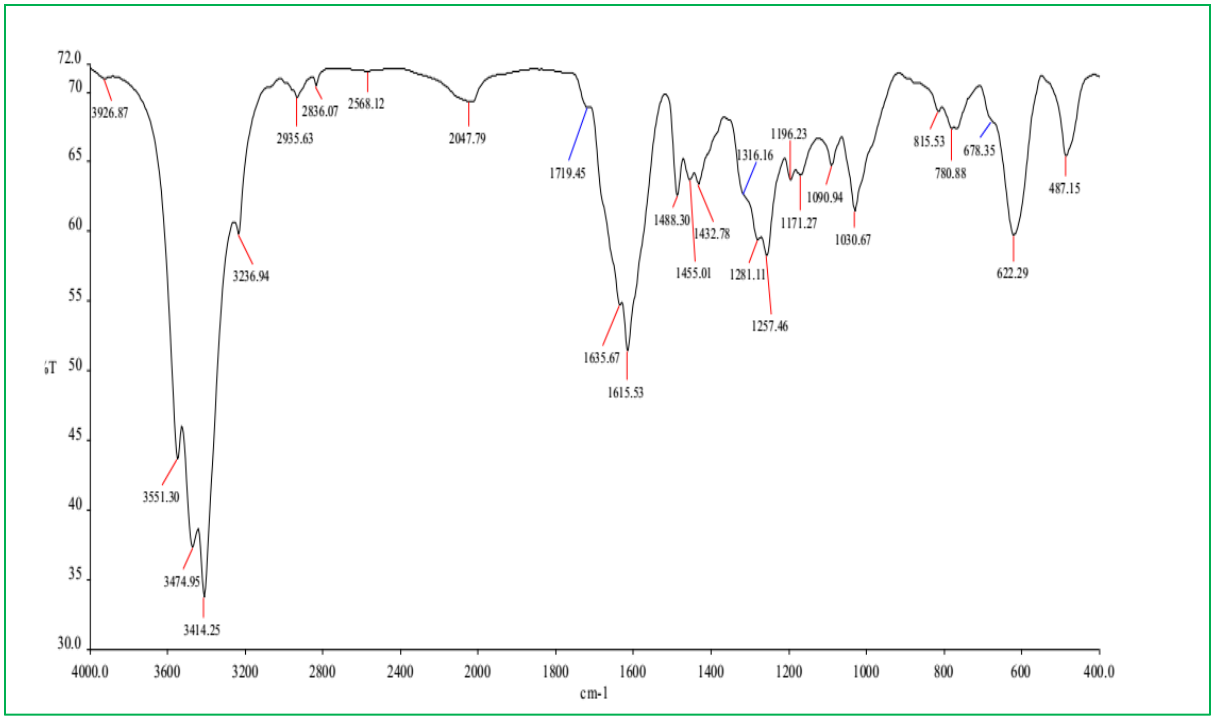

72.0
70
65
60
55
50
45
40
35
30.0
%T
4000.0
3600
3200
2800
2400
2000
1800
1600
1400
1200
1000
800
600
400.0
cm-1
3926.87
3551.30
3474.95
3414.25
3236.94
2935.63
2836.07
2568.12
2047.79
1719.45
1635.67
1615.53
1488.30
1455.01
1432.78
1316.16
1281.11
1257.46
1196.23
1171.27
1090.94
1030.67
815.53
780.88
678.35
622.29
487.15
