# Supplementary material for: Synthesis, Spectroscopic Characterization, Molecular Docking Studies Toward the GABAA Receptor, ADMET Prediction, GABA Aminotransferase Inhibition, and Anxiolytic Evaluation of Novel Thiazine Derivatives
Source: Pharmaceuticals (Basel). 2026 Aug 28;19(9):1364. doi: 10.3390/ph19091364 (PMC13610616; doi:10.3390/ph19091364)
Supplement: Supplementary file 1 [file pharmaceuticals-19-01364-s001.zip › C6 FT-IR.pdf]

C6 FT-IR

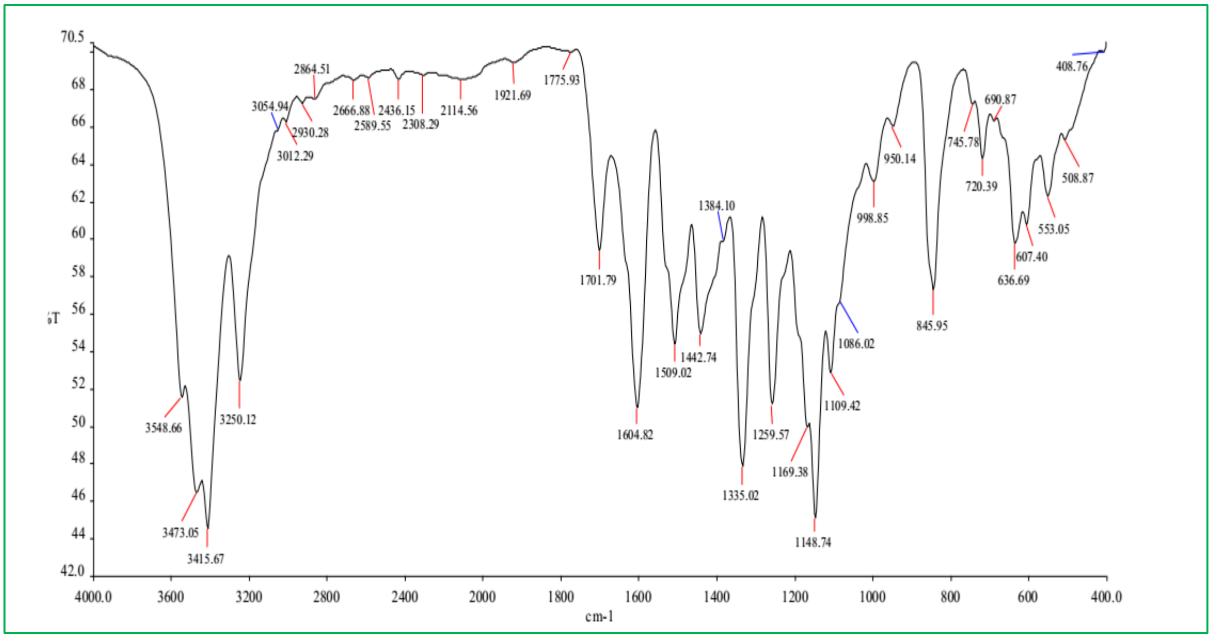

70.5
68
66
64
62
60
58
56
54
52
50
48
46
44
42.0
%T
4000.0
3600
3200
2800
2400
2000
1800
1600
1400
1200
1000
800
600
400.0
cm-1
3548.66
3473.05
3415.67
3250.12
3054.94
3012.29
2930.28
2864.51
2666.88
2589.55
2436.15
2308.29
2114.56
1921.69
1775.93
1701.79
1604.82
1509.02
1442.74
1384.10
1335.02
1259.57
1169.38
1148.74
1109.42
1086.02
998.85
950.14
845.95
745.78
720.39
690.87
636.69
607.40
553.05
508.87
408.76
