# Supplementary material for: Synthesis, Spectroscopic Characterization, Molecular Docking Studies Toward the GABAA Receptor, ADMET Prediction, GABA Aminotransferase Inhibition, and Anxiolytic Evaluation of Novel Thiazine Derivatives
Source: Pharmaceuticals (Basel). 2026 Aug 28;19(9):1364. doi: 10.3390/ph19091364 (PMC13610616; doi:10.3390/ph19091364)
Supplement: Supplementary file 1 [file pharmaceuticals-19-01364-s001.zip › C6 Mass spectrum.pdf]

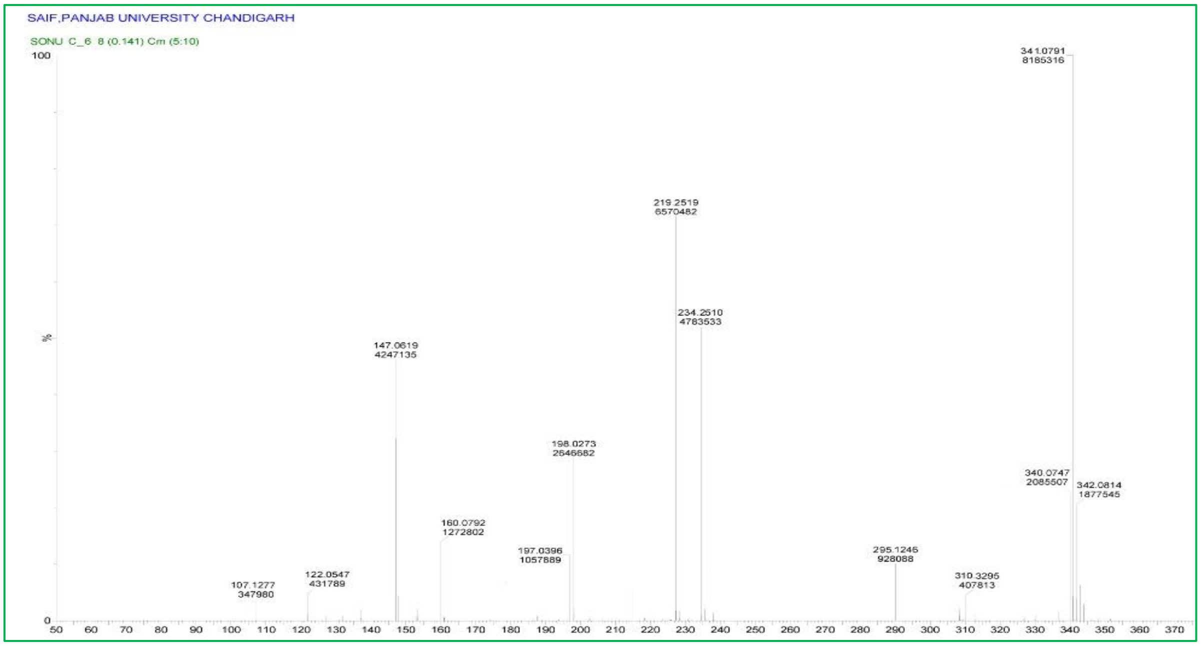

Mass spectrum of synthesized compound C6
