# Supplementary material for: Synthesis, Spectroscopic Characterization, Molecular Docking Studies Toward the GABAA Receptor, ADMET Prediction, GABA Aminotransferase Inhibition, and Anxiolytic Evaluation of Novel Thiazine Derivatives
Source: Pharmaceuticals (Basel). 2026 Aug 28;19(9):1364. doi: 10.3390/ph19091364 (PMC13610616; doi:10.3390/ph19091364)
Supplement: Supplementary file 1 [file pharmaceuticals-19-01364-s001.zip › C8 FT-IR.pdf]

C8 FT-IR

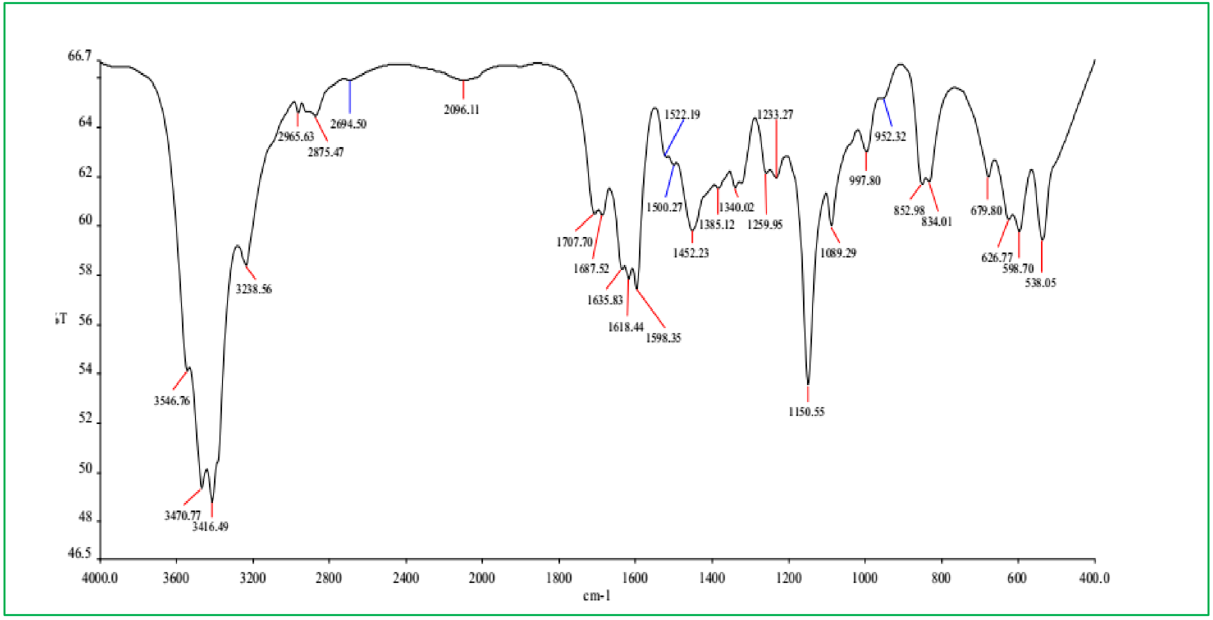
66.7
64
62
60
58
56
54
52
50
48
46.5
%T
4000.0
3600
3200
2800
2400
2000
1800
1600
1400
1200
1000
800
600
400.0
cm-1
3546.76
3470.77
3416.49
3238.56
2965.63
2875.47
2694.50
2096.11
1707.70
1687.52
1635.83
1618.44
1598.35
1522.19
1500.27
1452.23
1385.12
1340.02
1259.95
1233.27
1150.55
1089.29
997.80
952.32
852.98
834.01
679.80
626.77
598.70
538.05
