# Supplementary material for: Synthesis, Spectroscopic Characterization, Molecular Docking Studies Toward the GABAA Receptor, ADMET Prediction, GABA Aminotransferase Inhibition, and Anxiolytic Evaluation of Novel Thiazine Derivatives
Source: Pharmaceuticals (Basel). 2026 Aug 28;19(9):1364. doi: 10.3390/ph19091364 (PMC13610616; doi:10.3390/ph19091364)
Supplement: Supplementary file 1 [file pharmaceuticals-19-01364-s001.zip › Supplymentary File.pdf]

Supporting Information

Synthesis, Spectroscopic Characterization, Molecular Docking, ADMET Prediction, and Anxiolytic Evaluation of Novel Thiazine Derivatives Targeting the GABA$_A$ Receptor

Sonu[1,2*], Kamal Y. Thajudeen[3], Saad Ali Alshehri[3], Mohammed Muqtader Ahmed[4], Mayur Porwal[5], Sagar Verma[2]

[1]Drug Design Laboratory, School of Pharmaceutical Sciences, IFTM University, 244102, Moradabad, India

[2]Department of Pharmaceutical Chemistry, Shri Ram College of Pharmacy, Muzaffarnagar, 251001, India

[3]Department of Pharmacognosy, College of Pharmacy, King Khalid University, Abha, 62529, Saudi Arabia

[4]Department of Pharmaceutics, College of Pharmacy, Prince Sattam Bin Abdulaziz University, P.O. Box 173, AlKharj 11942, Saudi Arabia

[5]Teerthanker Mahaveer College of Pharmacy, Teerthanker Mahaveer University, Moradabad-244001, Uttar Pradesh, India

[*]Corresponding author

Sonu

E-mail:katariasonu376@gmail.com

ORCID ID: 0009-0004-7252-6447

Institutional Animal Ethics Committee
IFTM University, Moradabad

Ref No: IAEC/2022/33 **Date:** 27/01/2022

Registration No.: 837/PO/ReBiBt/S/04/CPCSEA

CERTIFICATE OF APPROVAL

This is to certify that the research project "**Synthesis and Computational Studies of Some New Heterocyclic Compounds of Biological Interest.**" submitted by **Mr. Sonu** has been approved in the Institutional Animal Ethics Committee (IAEC) meeting held on **27/01/2022** vide resolution No. **837/PO/ReBiBt/S/04/CPCSEA/33**. IAEC permitted him to carry out preclinical experiments on rats as per guidelines set by the Committee for the Purpose of Control and Supervision of Experiments on Animals (CPCSEA), Ministry of Fisheries, Animal Husbandry and Dairying, Department of Animal Husbandry and Dairying, Ministry of Agriculture and Farmers Welfare, Government of India.

Dr. Navneet Verma
Chairman (IAEC)

IAEC CHAIRMAN IFTM UNIVERSITY, MORADABAD

^{1}H NMR, ^{13}C NMR OF C1-C10 COMPOUNDS

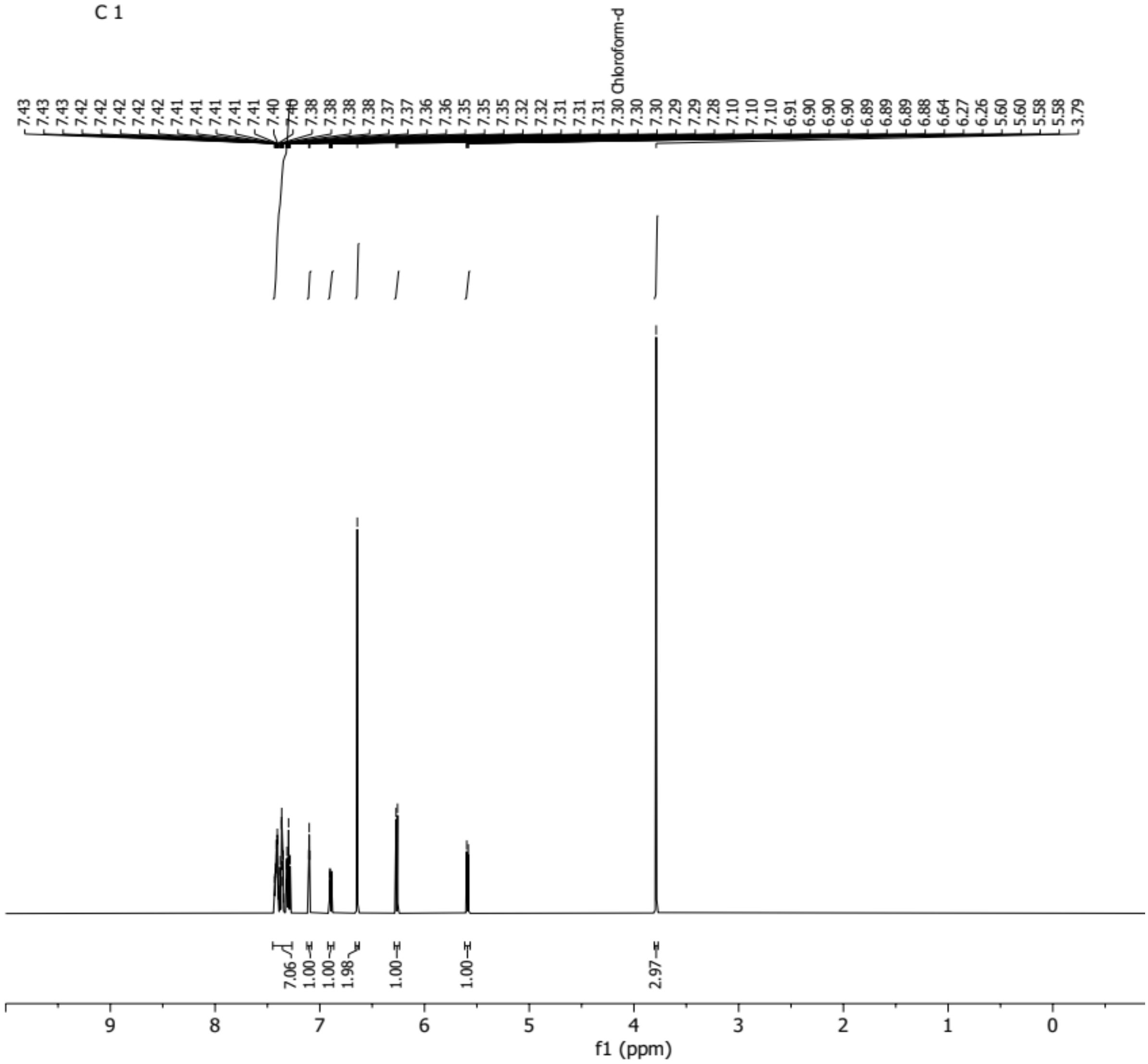

C 1

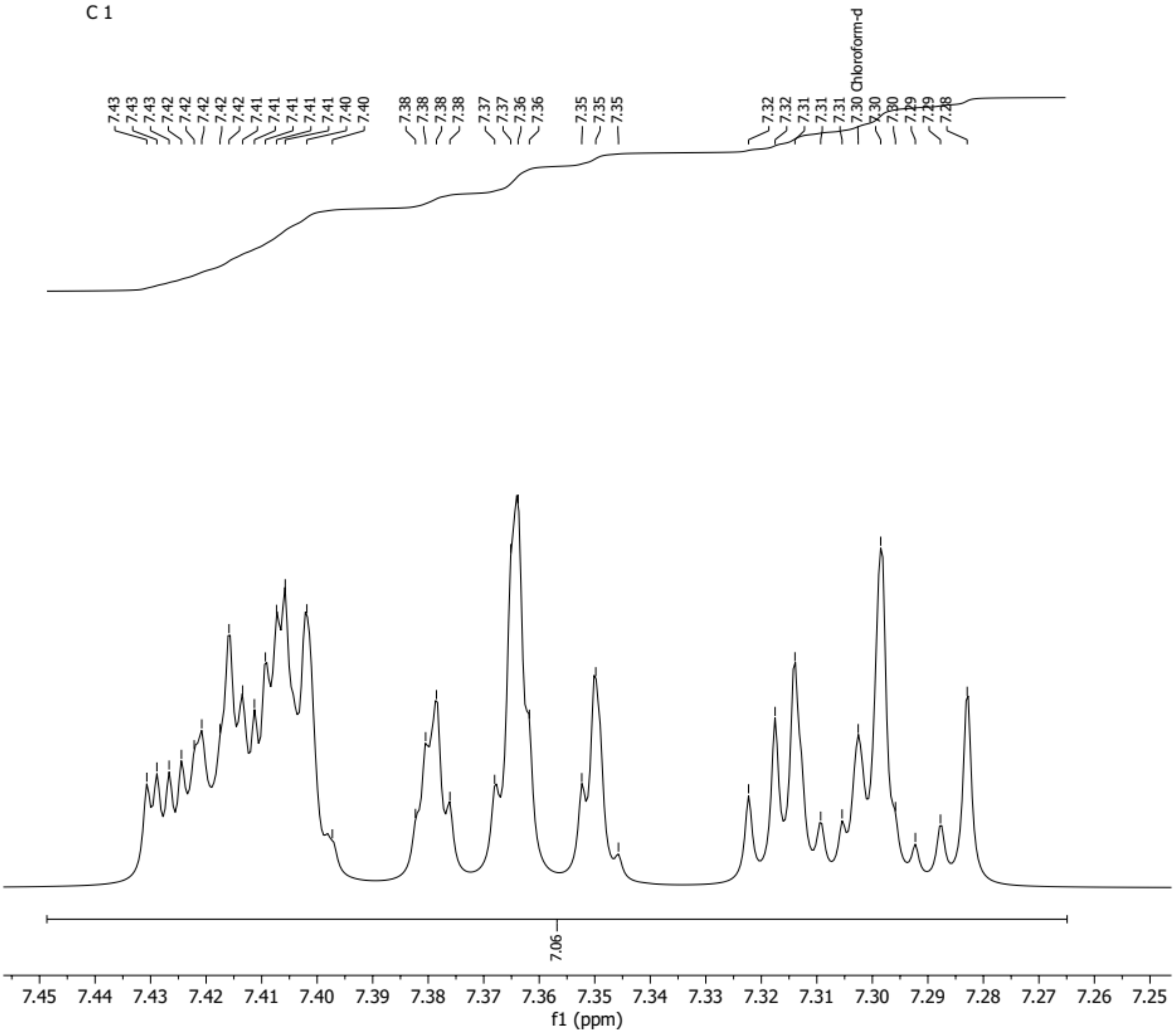

7.43
7.43
7.43
7.42
7.42
7.42
7.42
7.41
7.41
7.41
7.41
7.41
7.40
7.40
7.38
7.38
7.38
7.38
7.37
7.37
7.36
7.36
7.35
7.35
7.35
7.32
7.32
7.31
7.31
7.31
Chloroform-d
7.30
7.30
7.30
7.29
7.29
7.28
7.06
7.45 7.44 7.43 7.42 7.41 7.40 7.39 7.38 7.37 7.36 7.35 7.34 7.33 7.32 7.31 7.30 7.29 7.28 7.27 7.26 7.25
f1 (ppm)

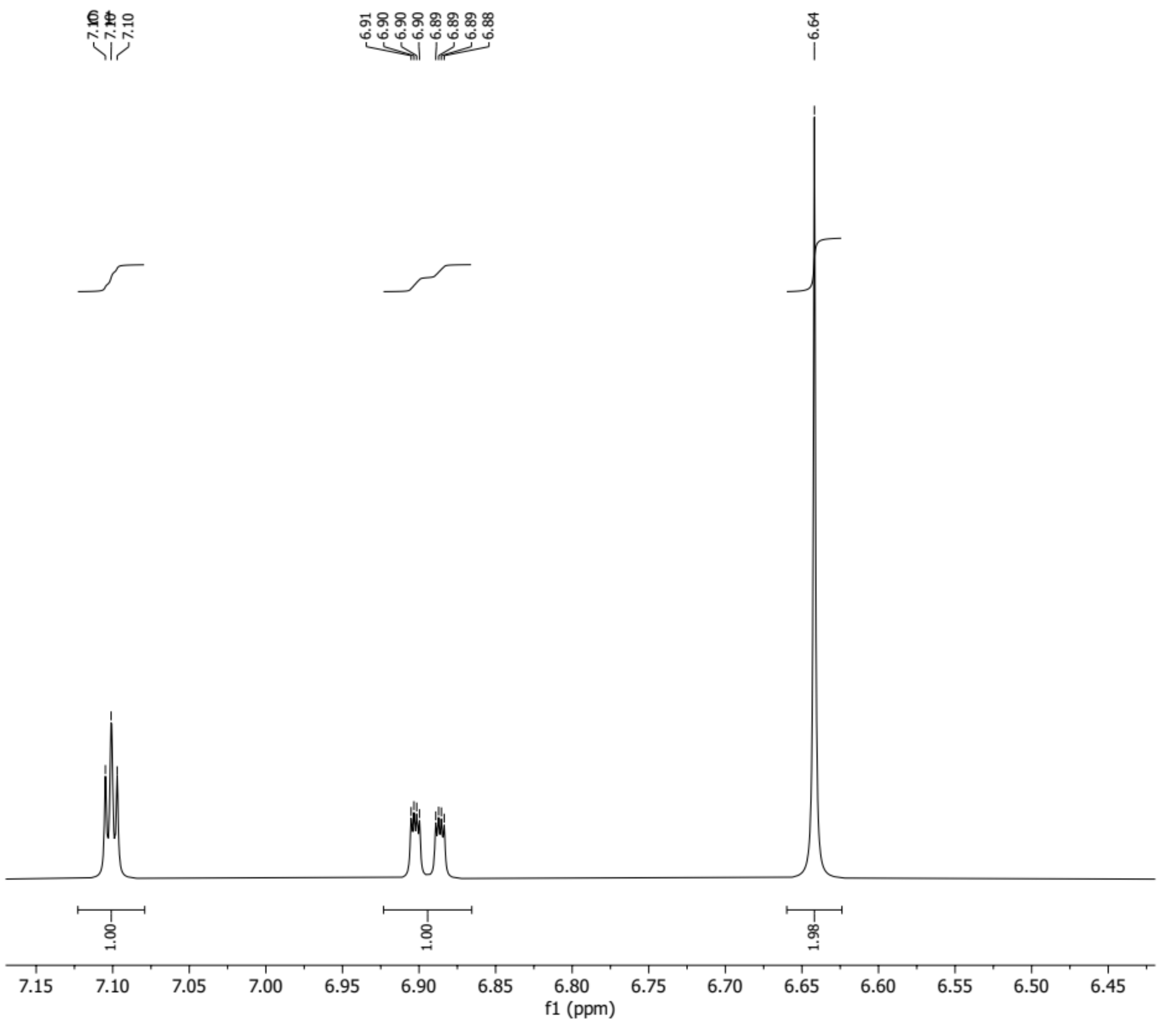
7.10
7.10
7.10
6.91
6.90
6.90
6.90
6.89
6.89
6.89
6.88
6.64
1.00
1.00
1.98
7.15 7.10 7.05 7.00 6.95 6.90 6.85 6.80 6.75 6.70 6.65 6.60 6.55 6.50 6.45
f1 (ppm)

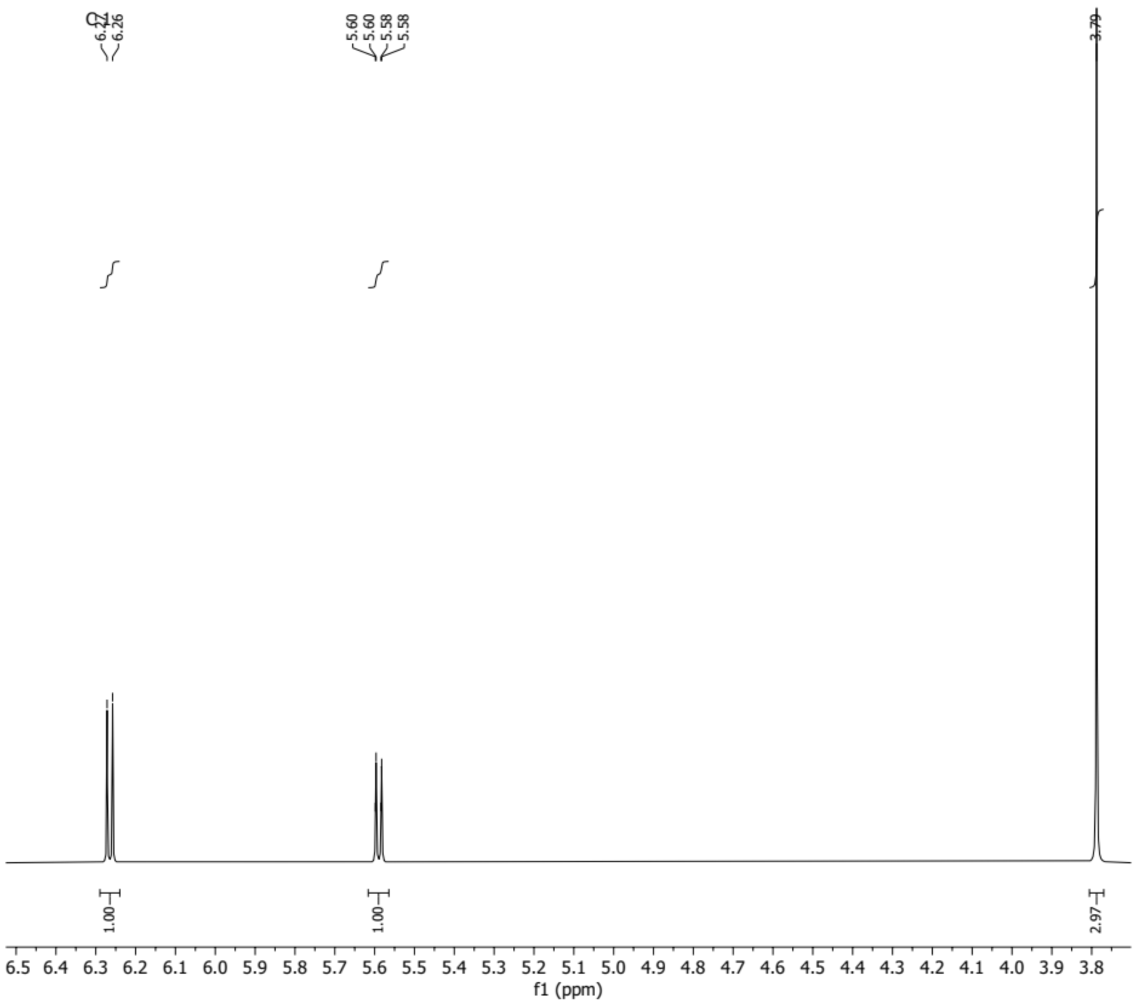

^{1}H NMR Spectrum of synthesized compound C1

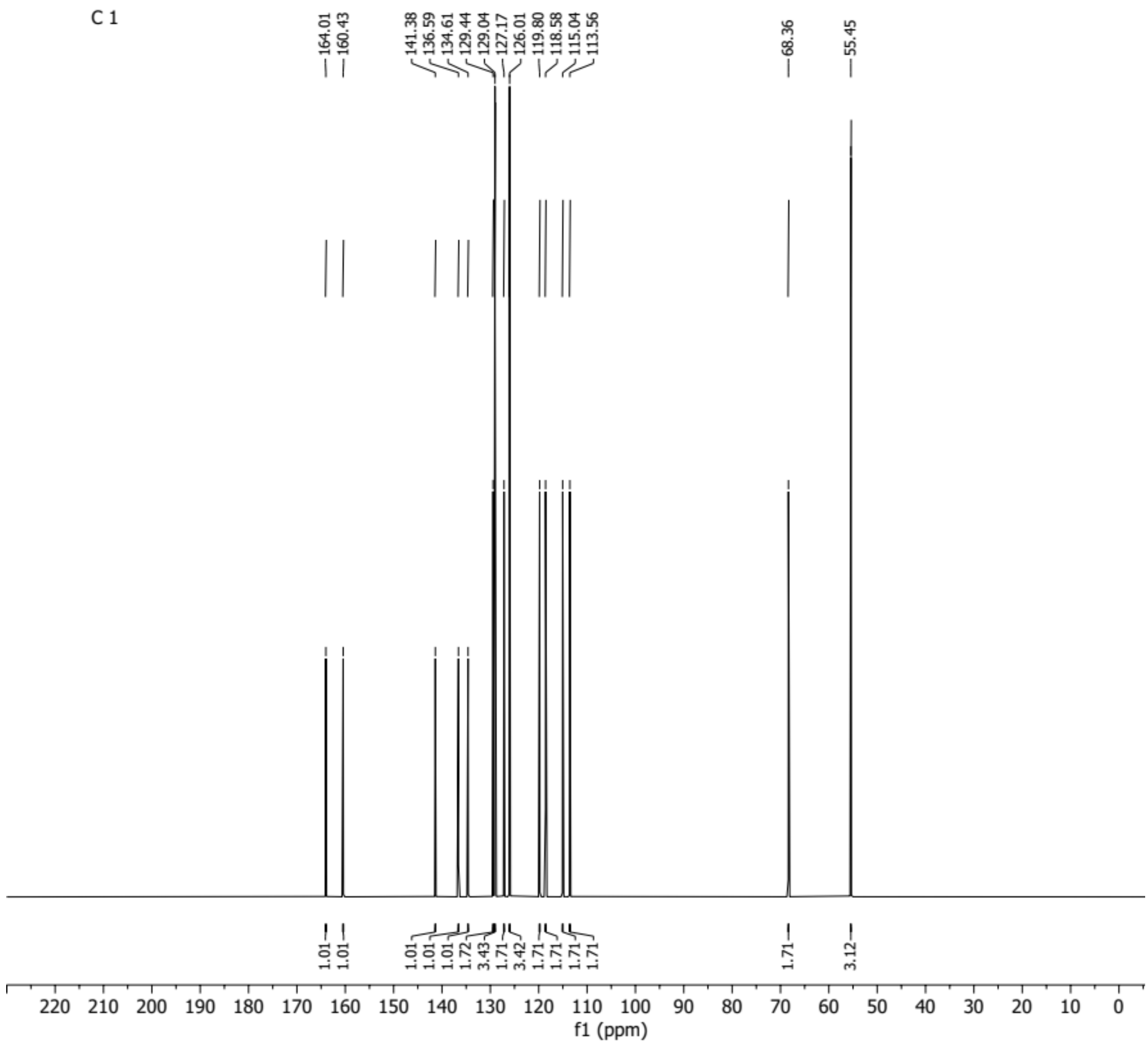

^{13}C spectrum of synthesized compound C1

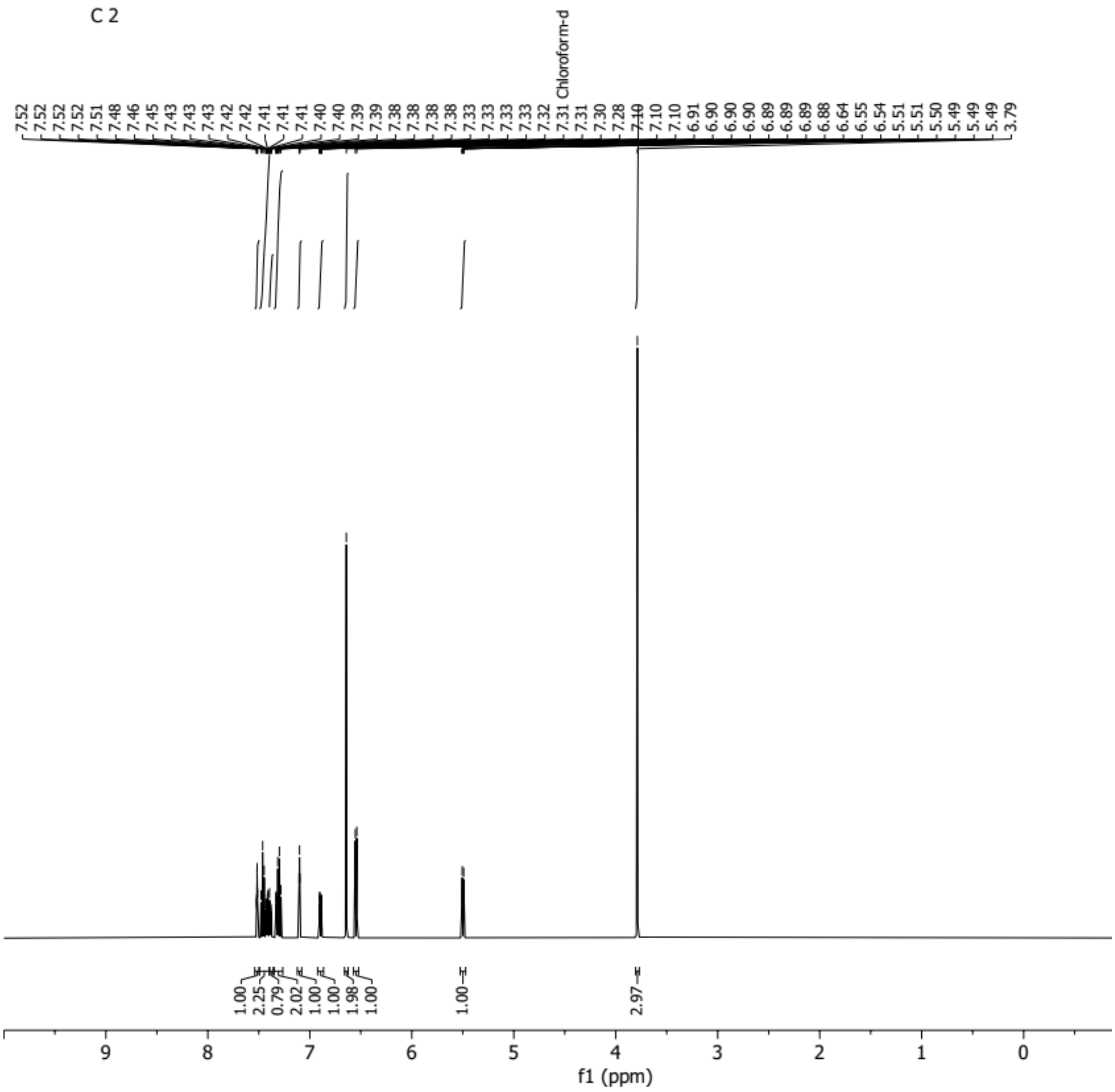
C 2
Chloroform-d
7.52
7.52
7.52
7.51
7.48
7.46
7.45
7.43
7.43
7.43
7.42
7.42
7.41
7.41
7.41
7.40
7.40
7.39
7.39
7.38
7.38
7.38
7.38
7.33
7.33
7.33
7.33
7.32
7.31
7.31
7.30
7.28
7.10
7.10
7.10
6.91
6.90
6.90
6.90
6.89
6.89
6.89
6.88
6.64
6.55
6.54
5.51
5.51
5.50
5.49
5.49
3.79
1.00
2.25
0.79
2.02
1.00
1.00
1.98
1.00
1.00
2.97
9
8
7
6
5
4
3
2
1
0
f1 (ppm)

C 2

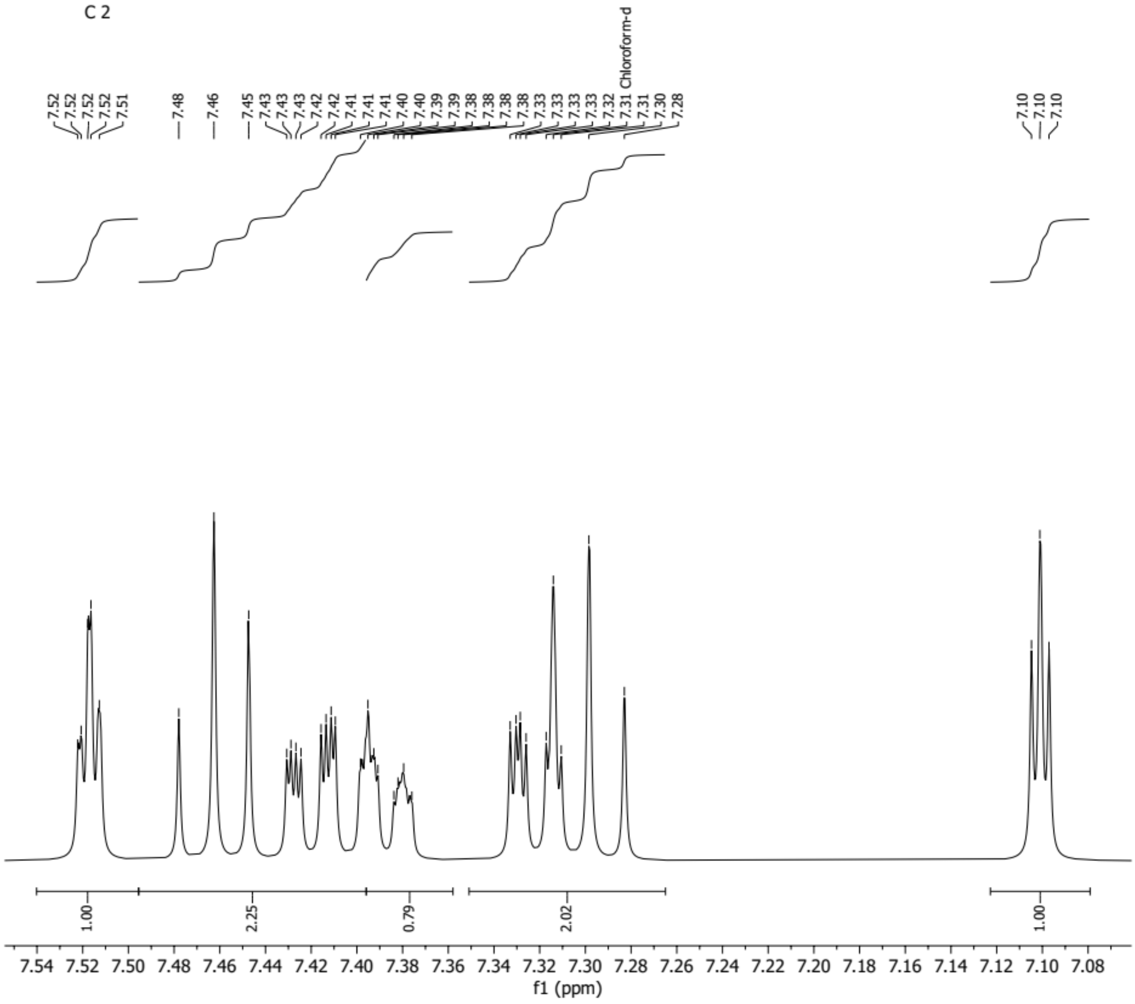

7.52
7.52
7.52
7.52
7.51
7.48
7.46
7.45
7.43
7.43
7.43
7.42
7.42
7.41
7.41
7.41
7.40
7.40
7.39
7.39
7.38
7.38
7.38
7.38
7.38
7.33
7.33
7.33
7.33
7.32
7.31 Chloroform-d
7.31
7.30
7.28
7.10
7.10
7.10
1.00
2.25
0.79
2.02
1.00
7.54 7.52 7.50 7.48 7.46 7.44 7.42 7.40 7.38 7.36 7.34 7.32 7.30 7.28 7.26 7.24 7.22 7.20 7.18 7.16 7.14 7.12 7.10 7.08
f1 (ppm)

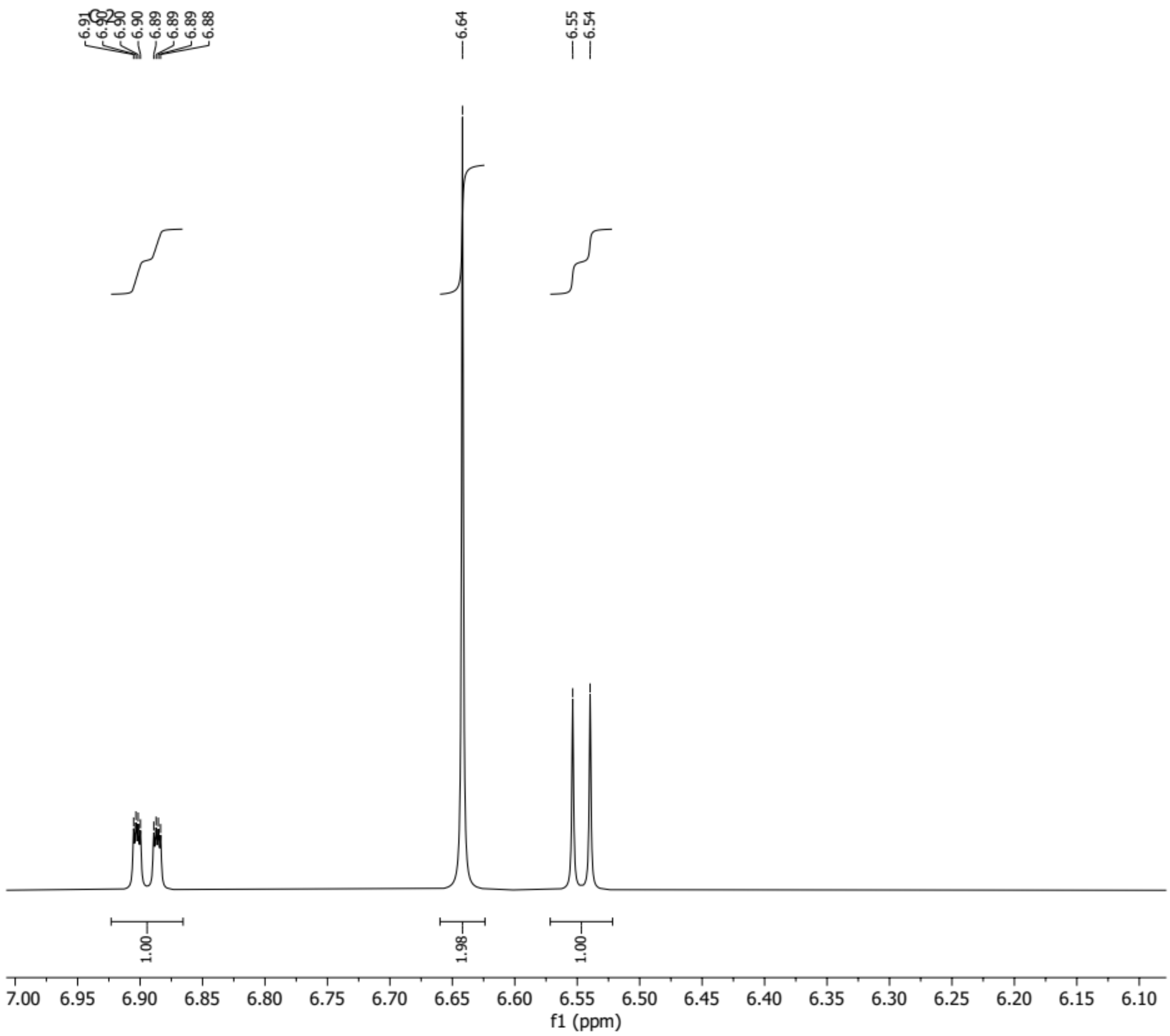

6.91
6.90
6.90
6.89
6.89
6.89
6.88
6.64
6.55
6.54
1.00
1.98
1.00
7.00 6.95 6.90 6.85 6.80 6.75 6.70 6.65 6.60 6.55 6.50 6.45 6.40 6.35 6.30 6.25 6.20 6.15 6.10
f1 (ppm)

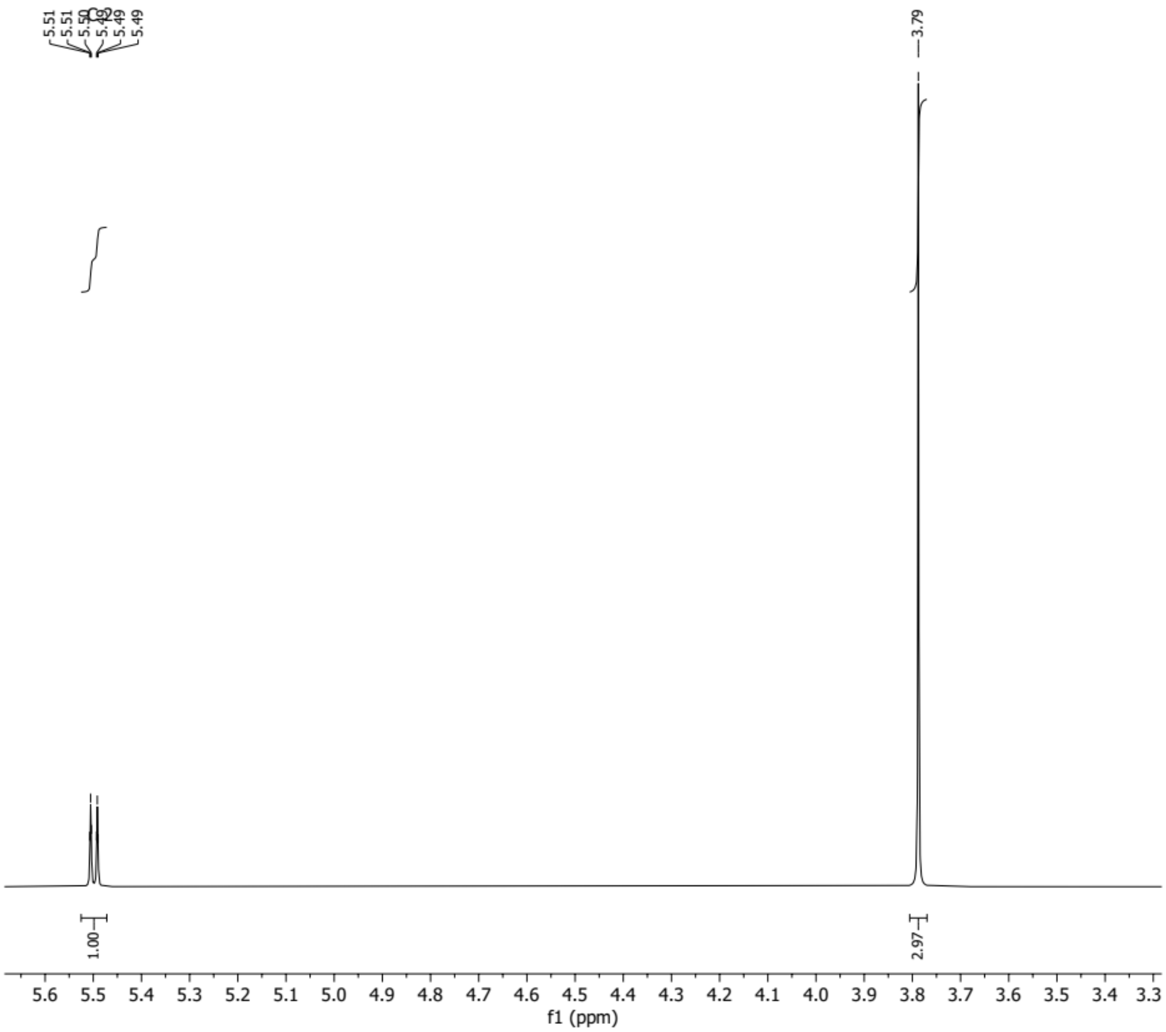

^{1}H NMR spectrum of synthesized compound C2

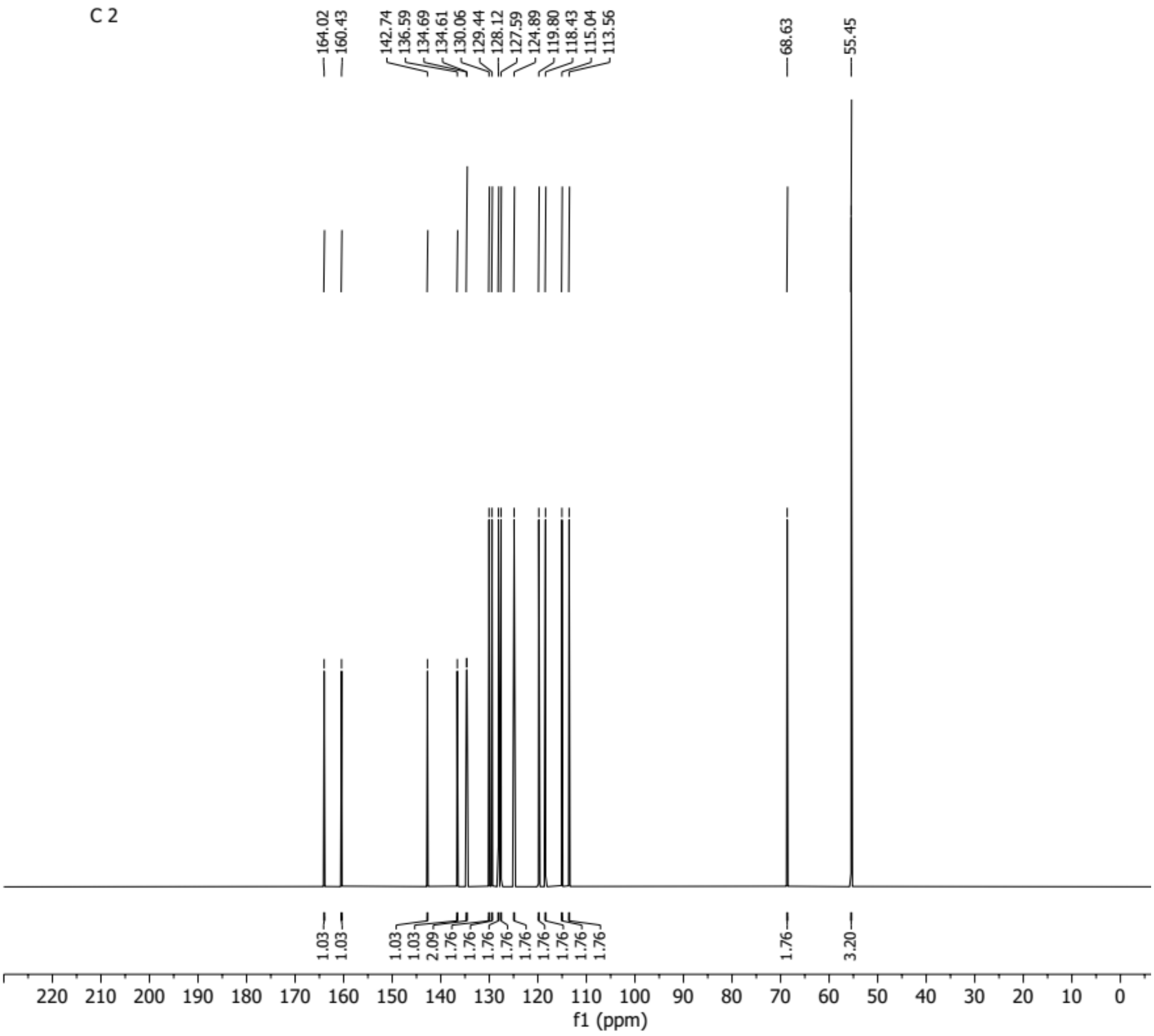

^{13}C NMR spectrum of synthesized compound C2

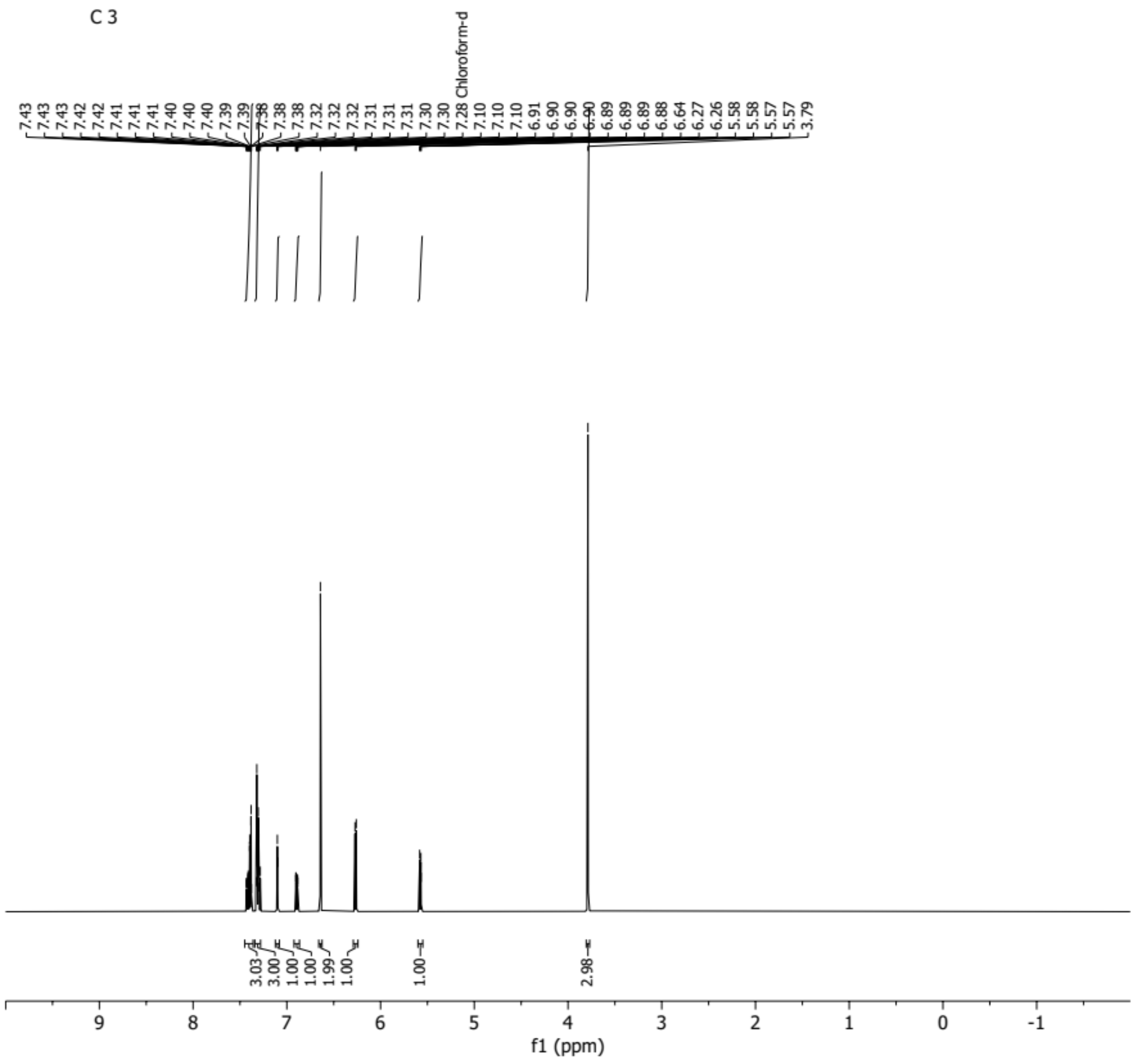

C 3
7.43
7.43
7.43
7.42
7.42
7.41
7.41
7.40
7.40
7.39
7.39
7.38
7.38
7.38
7.32
7.32
7.31
7.31
7.31
7.30
7.30
7.28 Chloroform-d
7.10
7.10
7.10
6.91
6.90
6.90
6.90
6.89
6.89
6.89
6.88
6.64
6.27
6.26
5.58
5.58
5.57
5.57
3.79
3.03
3.00
1.00
1.00
1.99
1.00
1.00
2.98
9
8
7
6
5
4
3
2
1
0
-1
f1 (ppm)

C 3

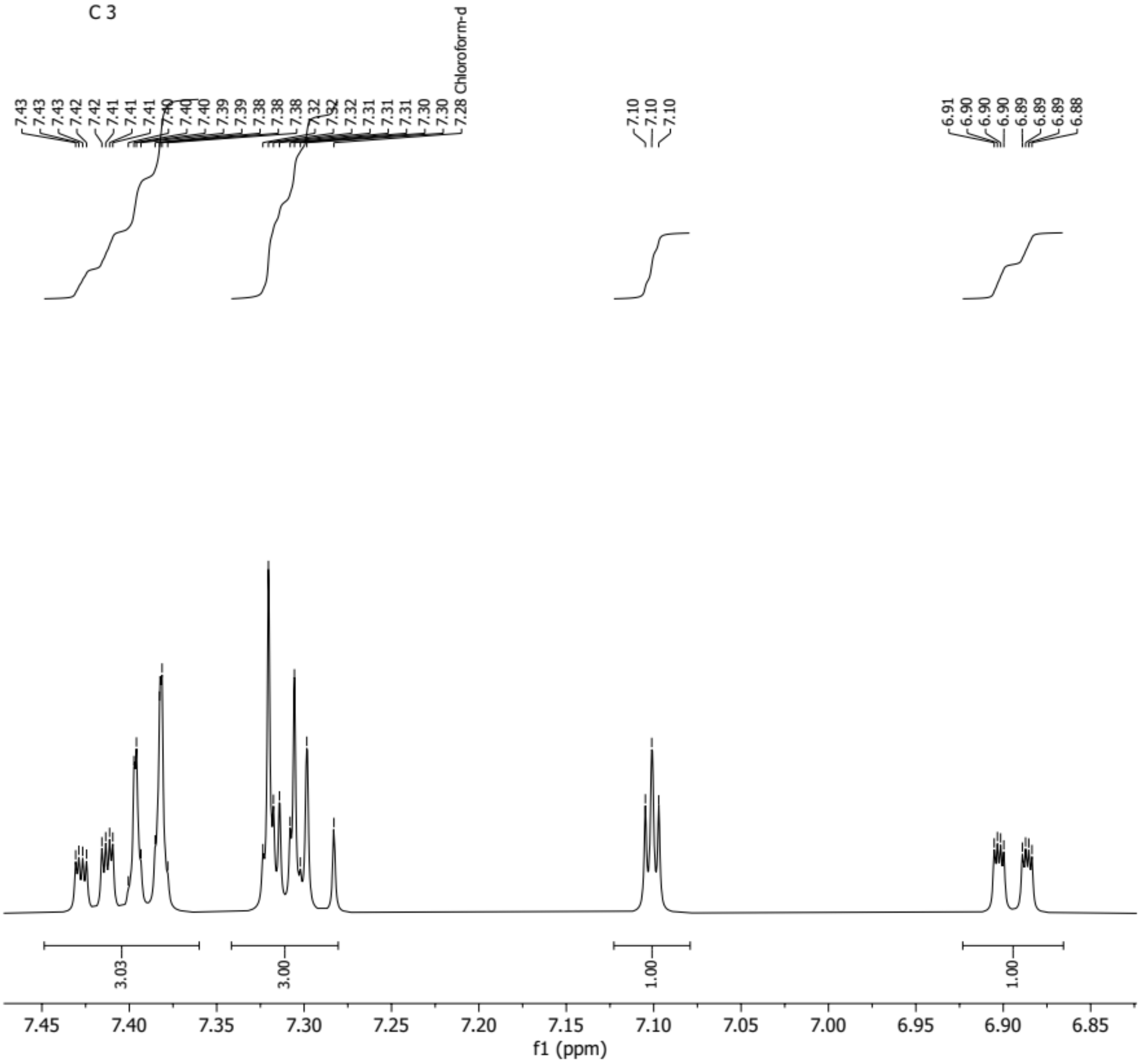
7.43
7.43
7.43
7.42
7.42
7.41
7.41
7.41
7.40
7.40
7.40
7.39
7.39
7.38
7.38
7.38
7.32
7.32
7.32
7.31
7.31
7.30
7.30
7.28 Chloroform-d
7.10
7.10
7.10
6.91
6.90
6.90
6.90
6.89
6.89
6.89
6.88
3.03
3.00
1.00
1.00
7.45
7.40
7.35
7.30
7.25
7.20
7.15
7.10
7.05
7.00
6.95
6.90
6.85
f1 (ppm)

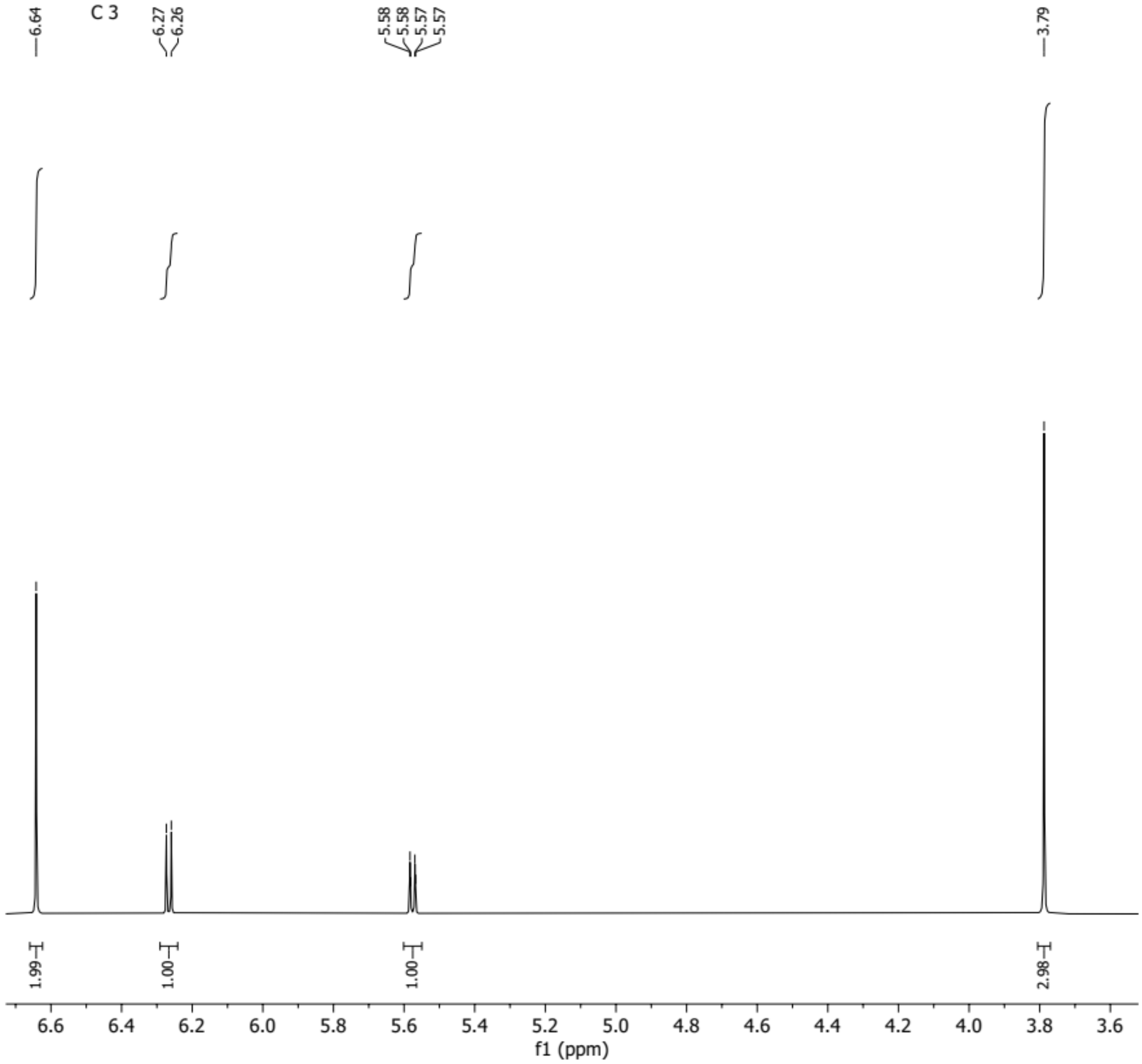

^{1}H NMR spectrum of synthesized compound C3

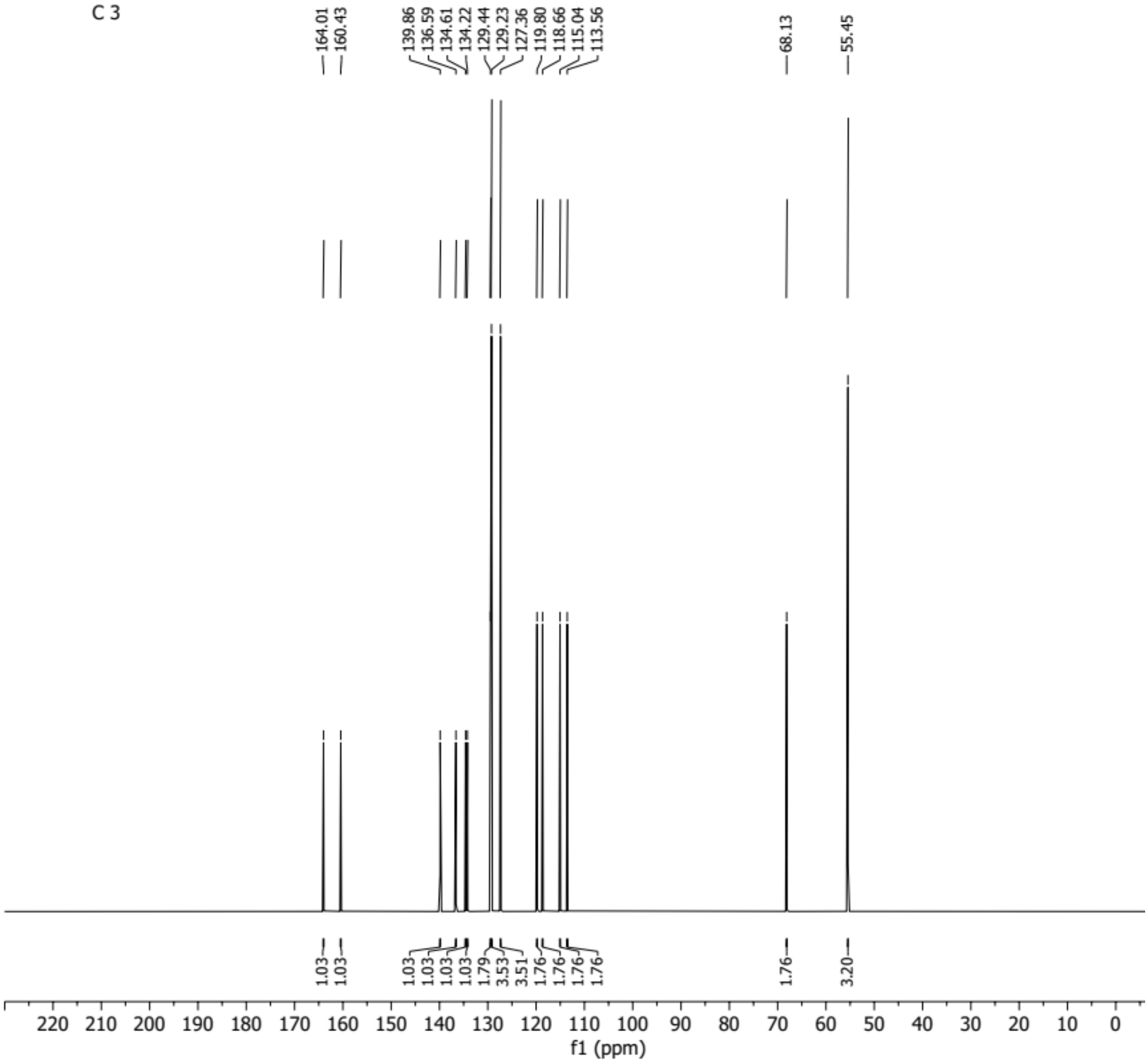

^{13}C NMR spectrum of synthesized compound C3

C 4

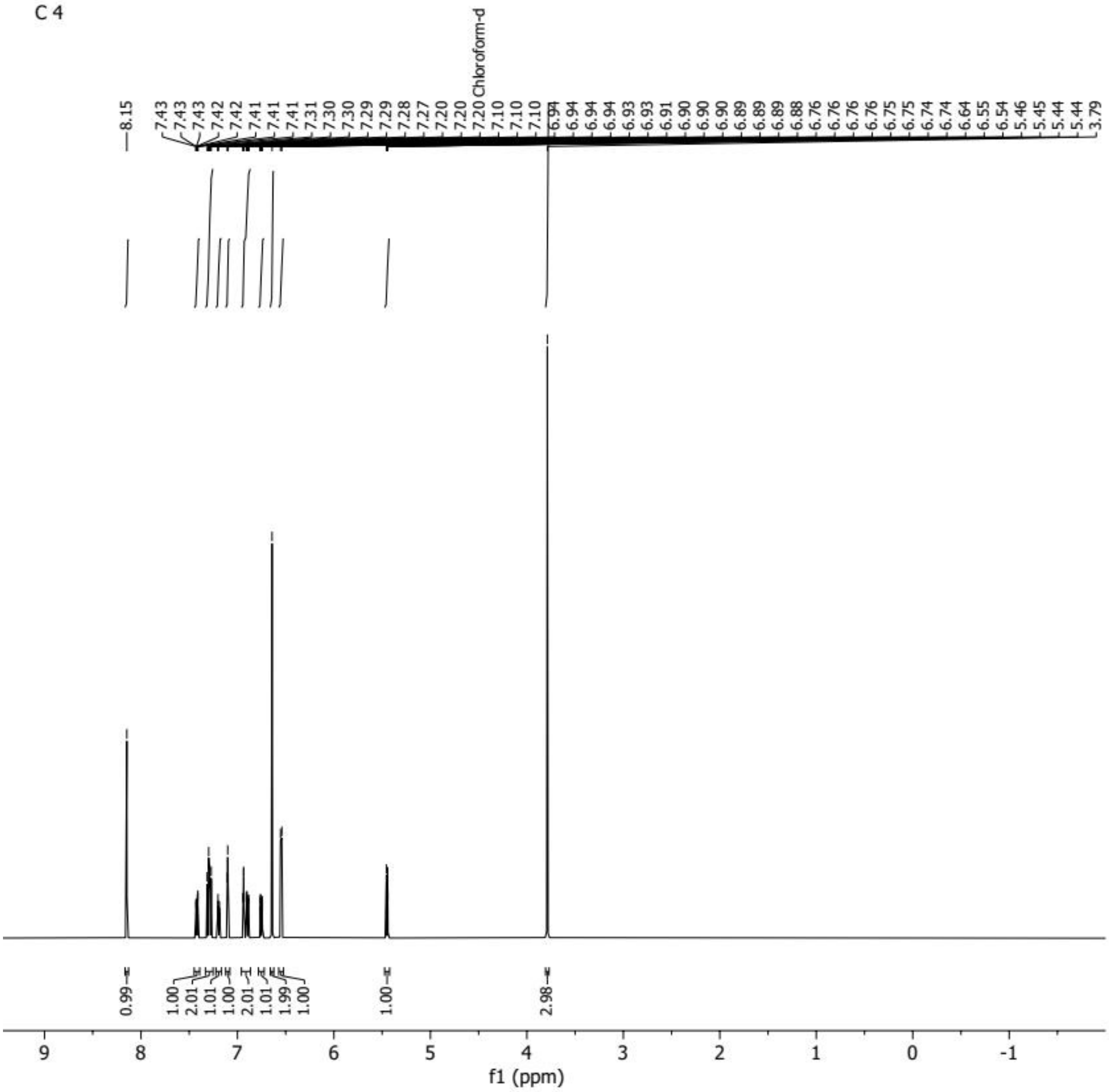

8.15
7.43
7.43
7.43
7.42
7.42
7.41
7.41
7.41
7.31
7.30
7.29
7.29
7.28
7.27
7.20
7.20
7.20 Chloroform-d
7.10
7.10
7.10
6.94
6.94
6.94
6.94
6.93
6.93
6.91
6.90
6.90
6.89
6.89
6.89
6.88
6.76
6.76
6.76
6.75
6.75
6.74
6.64
6.55
6.54
5.46
5.45
5.44
5.44
3.79
0.99
1.00
2.01
1.01
1.00
2.01
1.01
1.99
1.00
1.00
2.98
f1 (ppm)

C 4

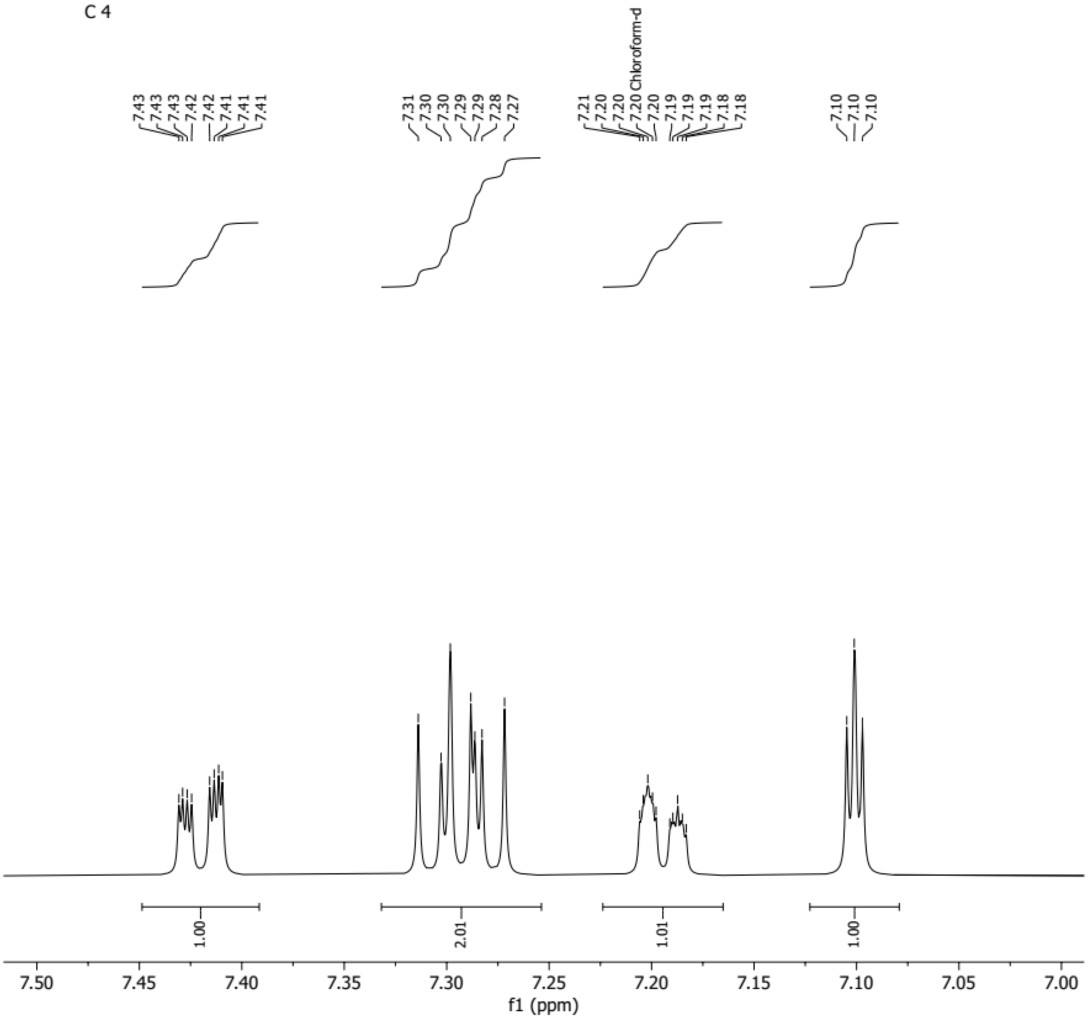
7.43
7.43
7.43
7.42
7.42
7.41
7.41
7.41
7.31
7.30
7.30
7.29
7.29
7.29
7.28
7.27
7.21
7.20
7.20
7.20 Chloroform-d
7.20
7.19
7.19
7.19
7.18
7.18
7.10
7.10
7.10
1.00
2.01
1.01
1.00
7.50
7.45
7.40
7.35
7.30
7.25
7.20
7.15
7.10
7.05
7.00
f1 (ppm)

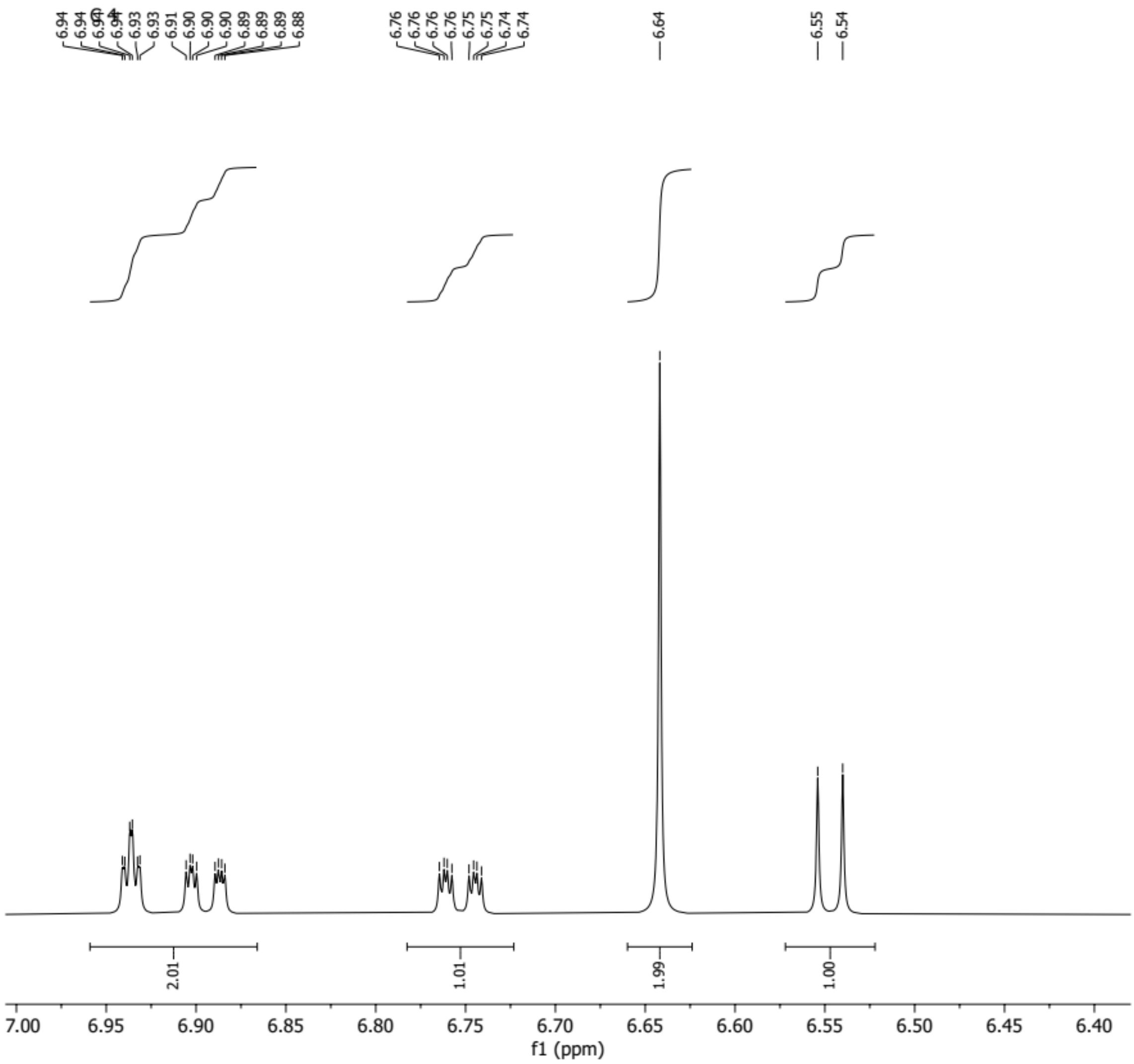
6.94
6.94
6.94
6.94
6.93
6.93
6.91
6.90
6.90
6.90
6.89
6.89
6.89
6.88
6.76
6.76
6.76
6.76
6.75
6.75
6.74
6.74
6.64
6.55
6.54
2.01
1.01
1.99
1.00
7.00
6.95
6.90
6.85
6.80
6.75
6.70
6.65
6.60
6.55
6.50
6.45
6.40
f1 (ppm)

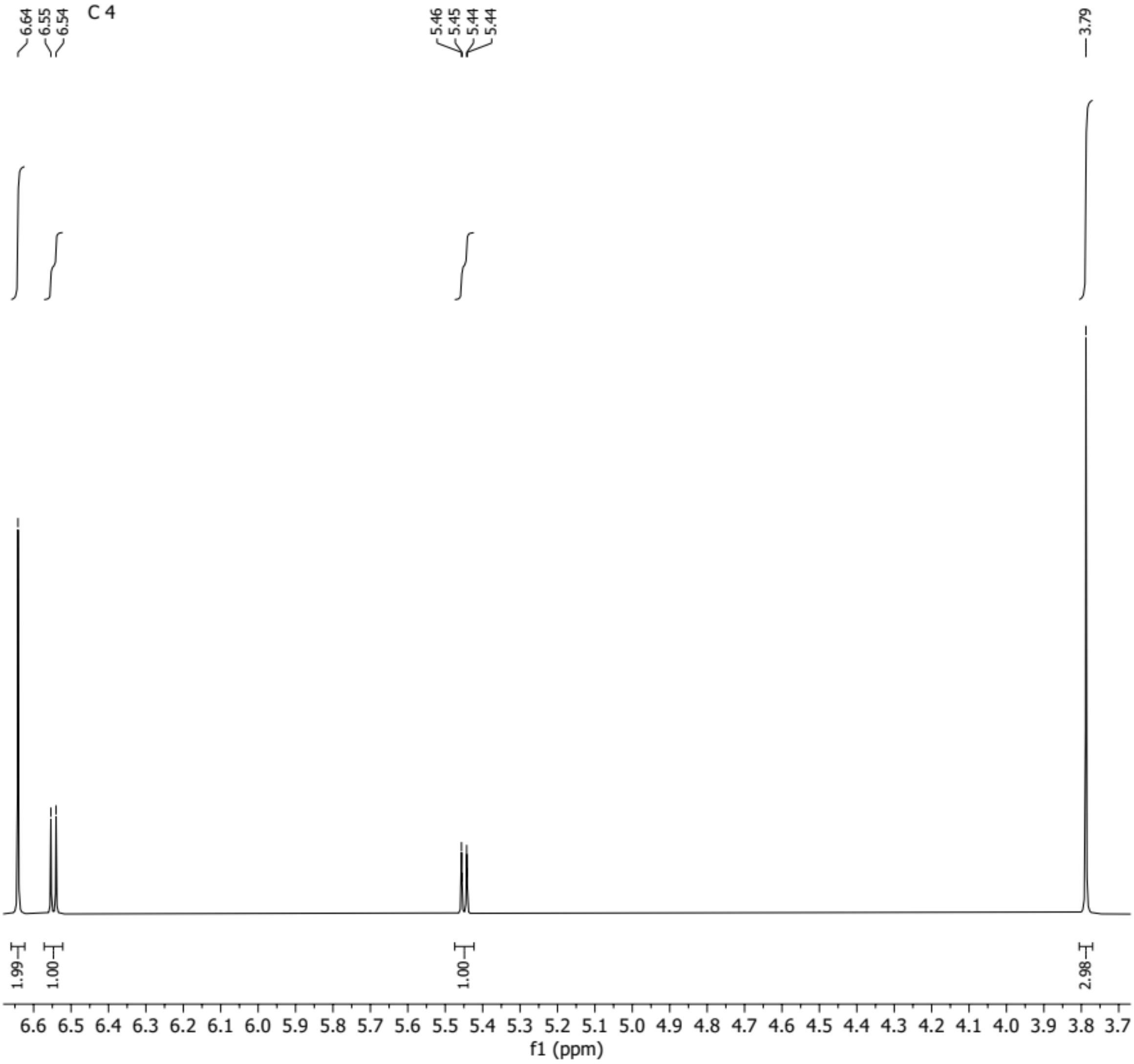

^{1}H NMR spectrum of synthesized compound C4

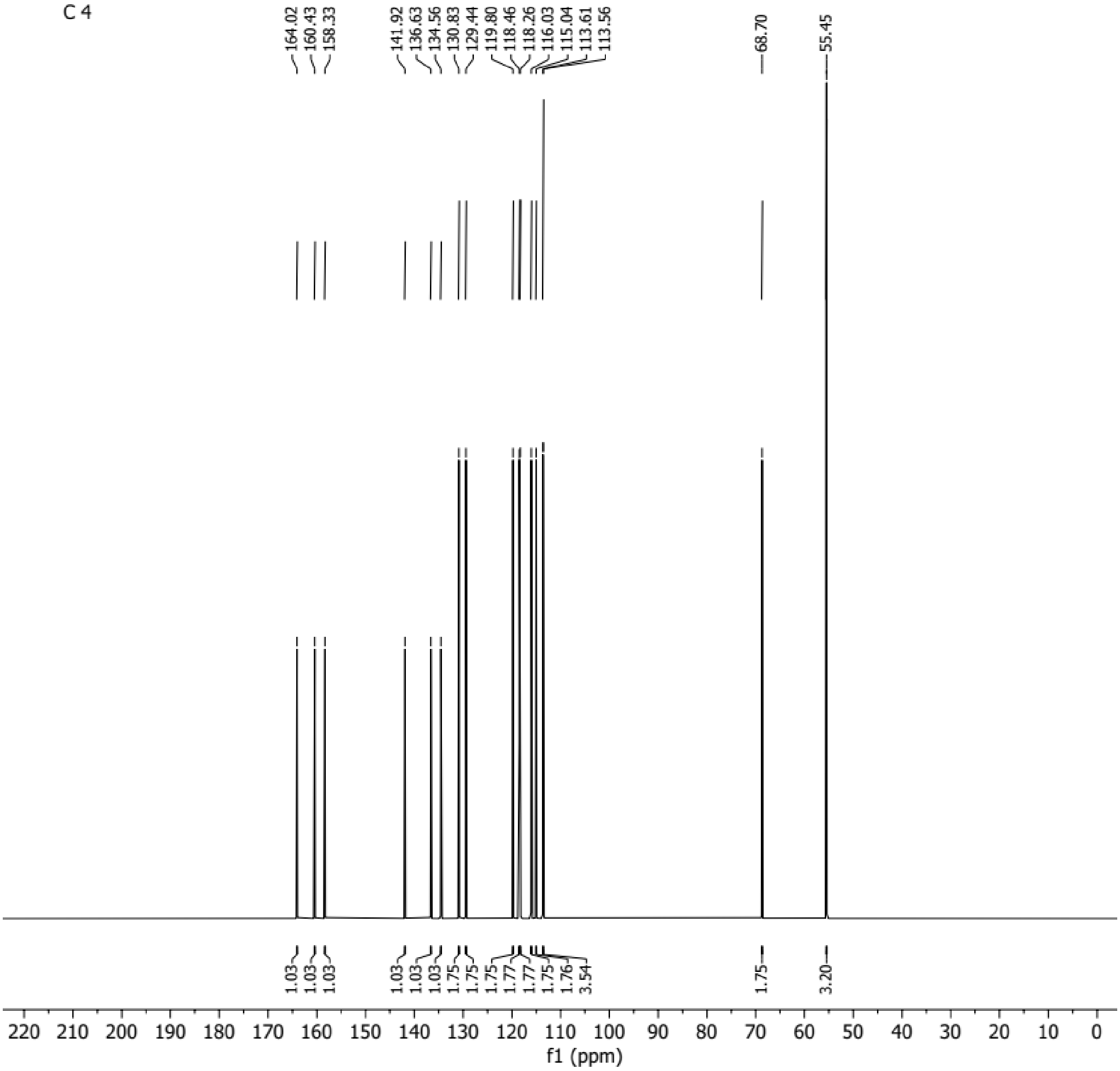

^{13}C NMR spectrum of synthesized compound C4

C 5

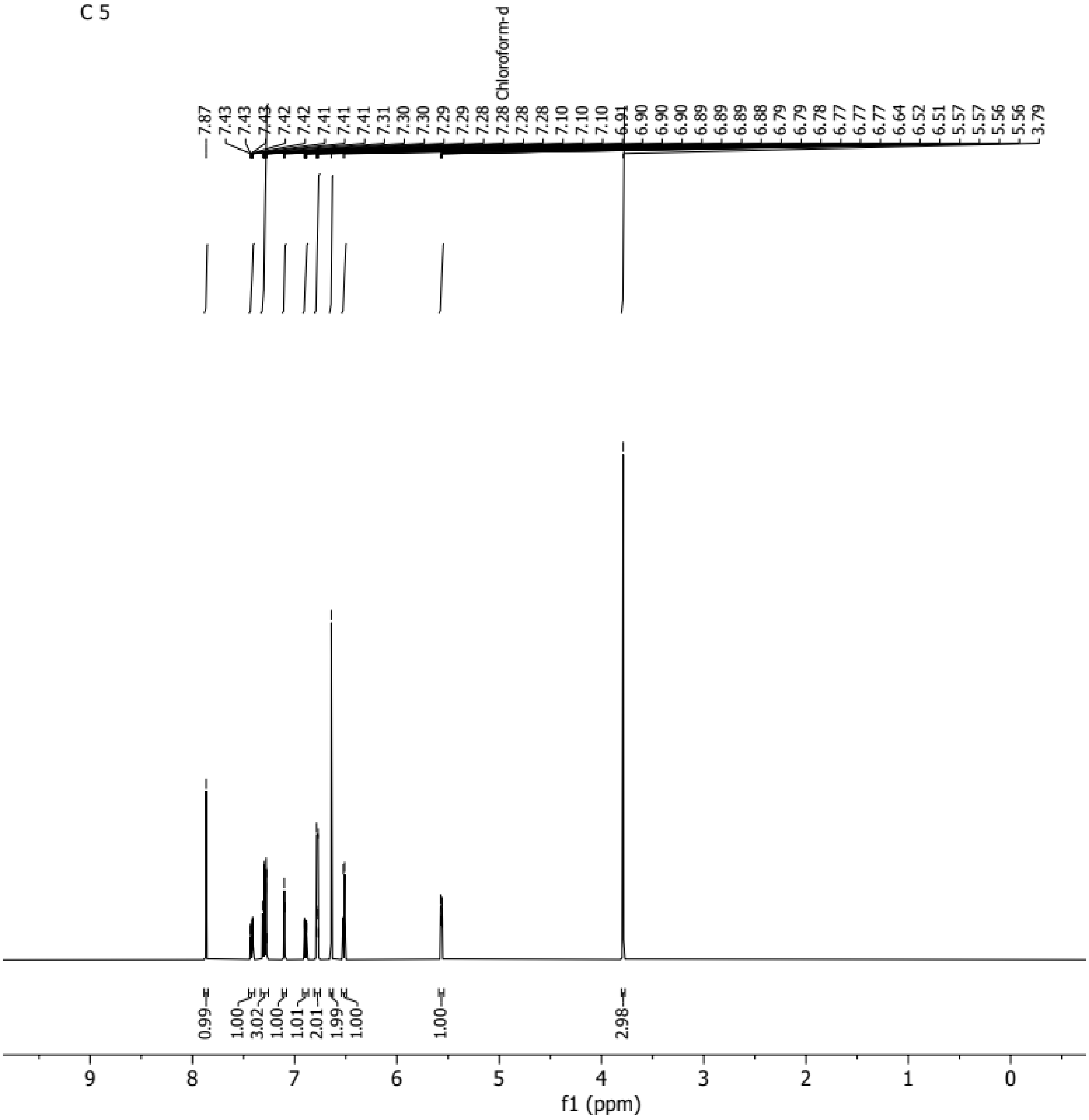
Chloroform-d
7.87
7.43
7.43
7.43
7.42
7.42
7.41
7.41
7.31
7.30
7.30
7.29
7.29
7.28
7.28
7.28
7.28
7.10
7.10
7.10
6.91
6.90
6.90
6.89
6.89
6.89
6.88
6.79
6.79
6.78
6.77
6.77
6.77
6.64
6.52
6.51
5.57
5.57
5.56
5.56
3.79
0.99
1.00
3.02
1.00
1.01
2.01
1.99
1.00
1.00
2.98
9
8
7
6
5
4
3
2
1
0
f1 (ppm)

C 5

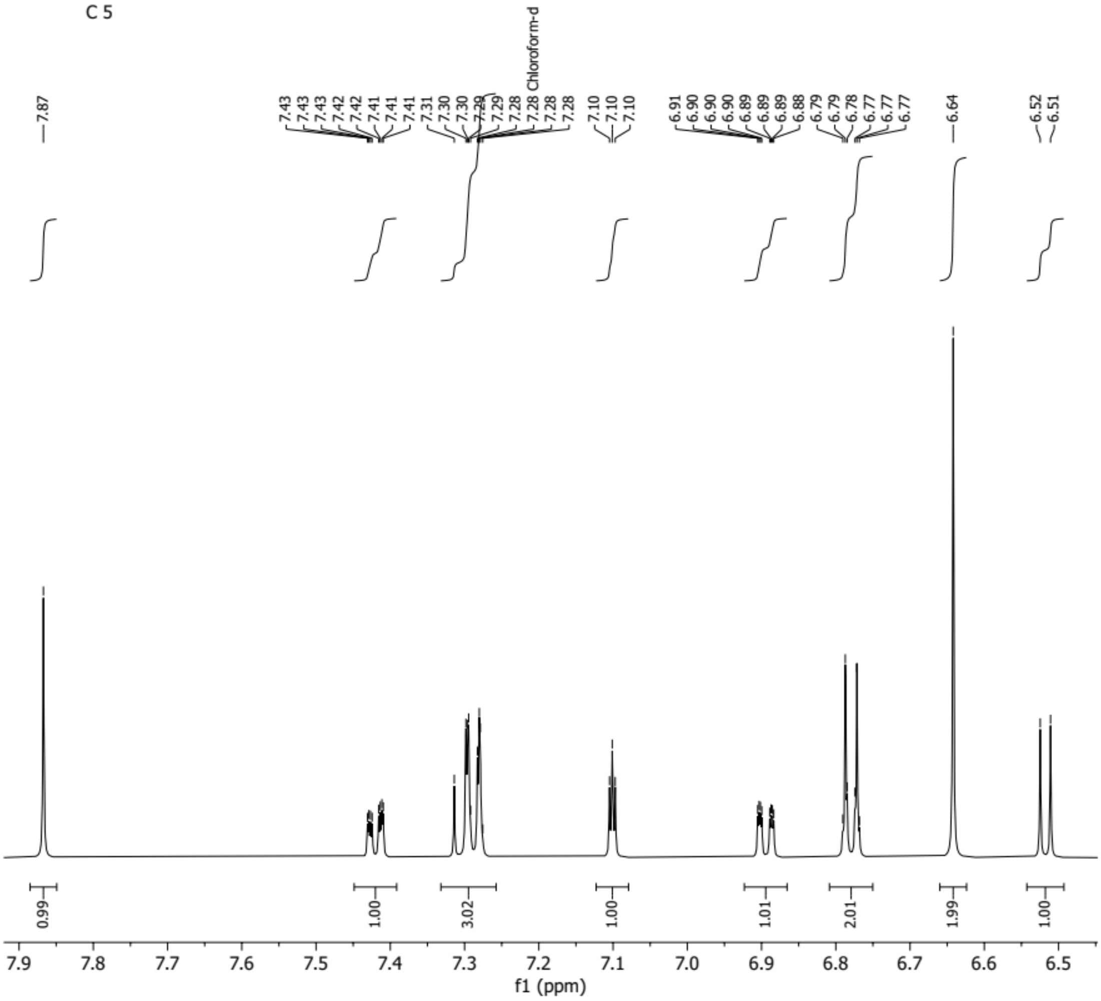

7.87
7.43
7.43
7.43
7.42
7.41
7.41
7.41
7.31
7.30
7.30
7.29
7.29
7.28 Chloroform-d
7.28
7.28
7.10
7.10
7.10
6.91
6.90
6.90
6.89
6.89
6.89
6.88
6.79
6.79
6.78
6.77
6.77
6.77
6.64
6.52
6.51
0.99
1.00
3.02
1.00
1.01
2.01
1.99
1.00
7.9
7.8
7.7
7.6
7.5
7.4
7.3
7.2
7.1
7.0
6.9
6.8
6.7
6.6
6.5
f1 (ppm)

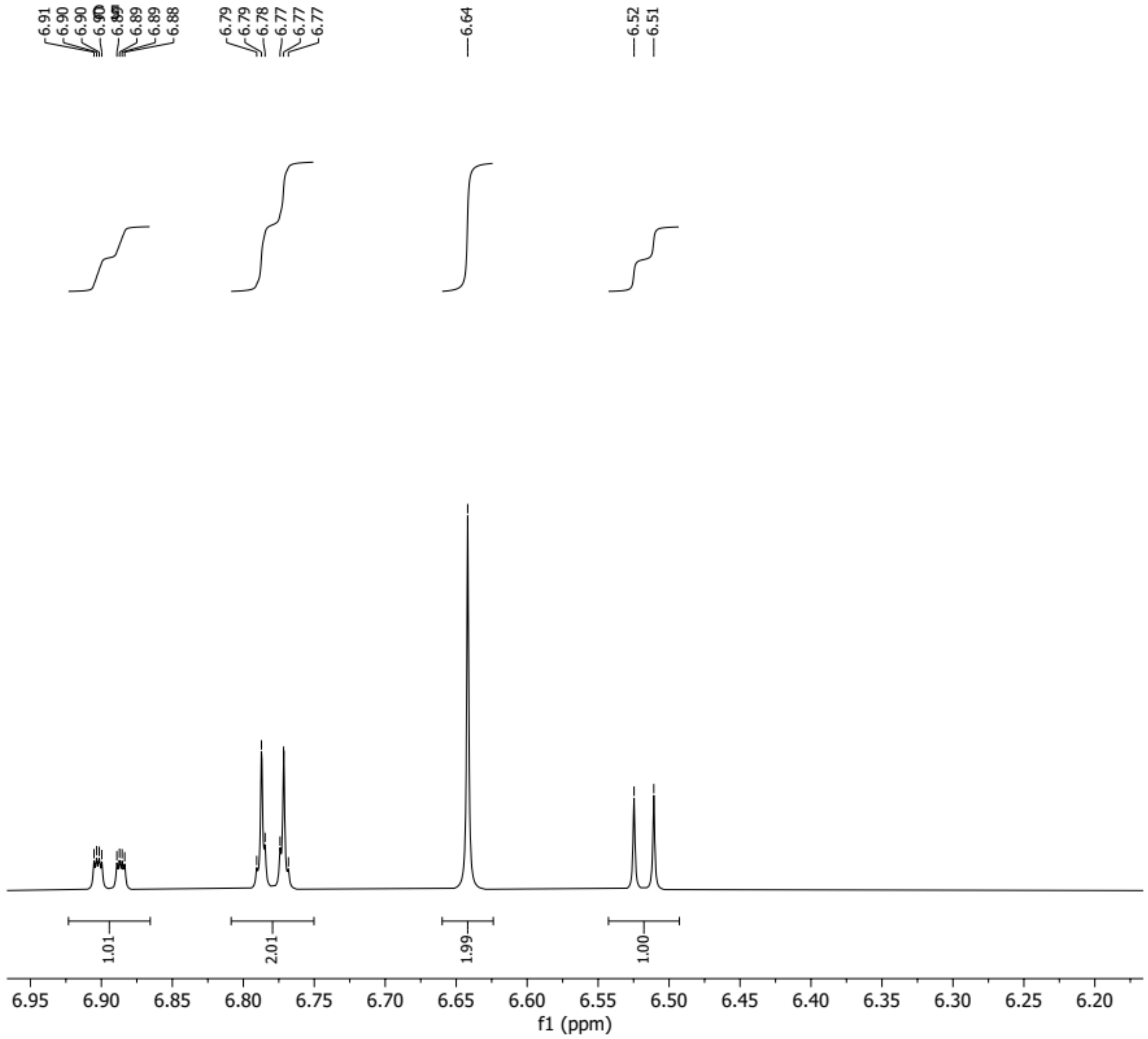

6.91
6.90
6.90
6.89
6.89
6.88
6.79
6.79
6.78
6.77
6.77
6.77
6.64
6.52
6.51
1.01
2.01
1.99
1.00
6.95 6.90 6.85 6.80 6.75 6.70 6.65 6.60 6.55 6.50 6.45 6.40 6.35 6.30 6.25 6.20
f1 (ppm)

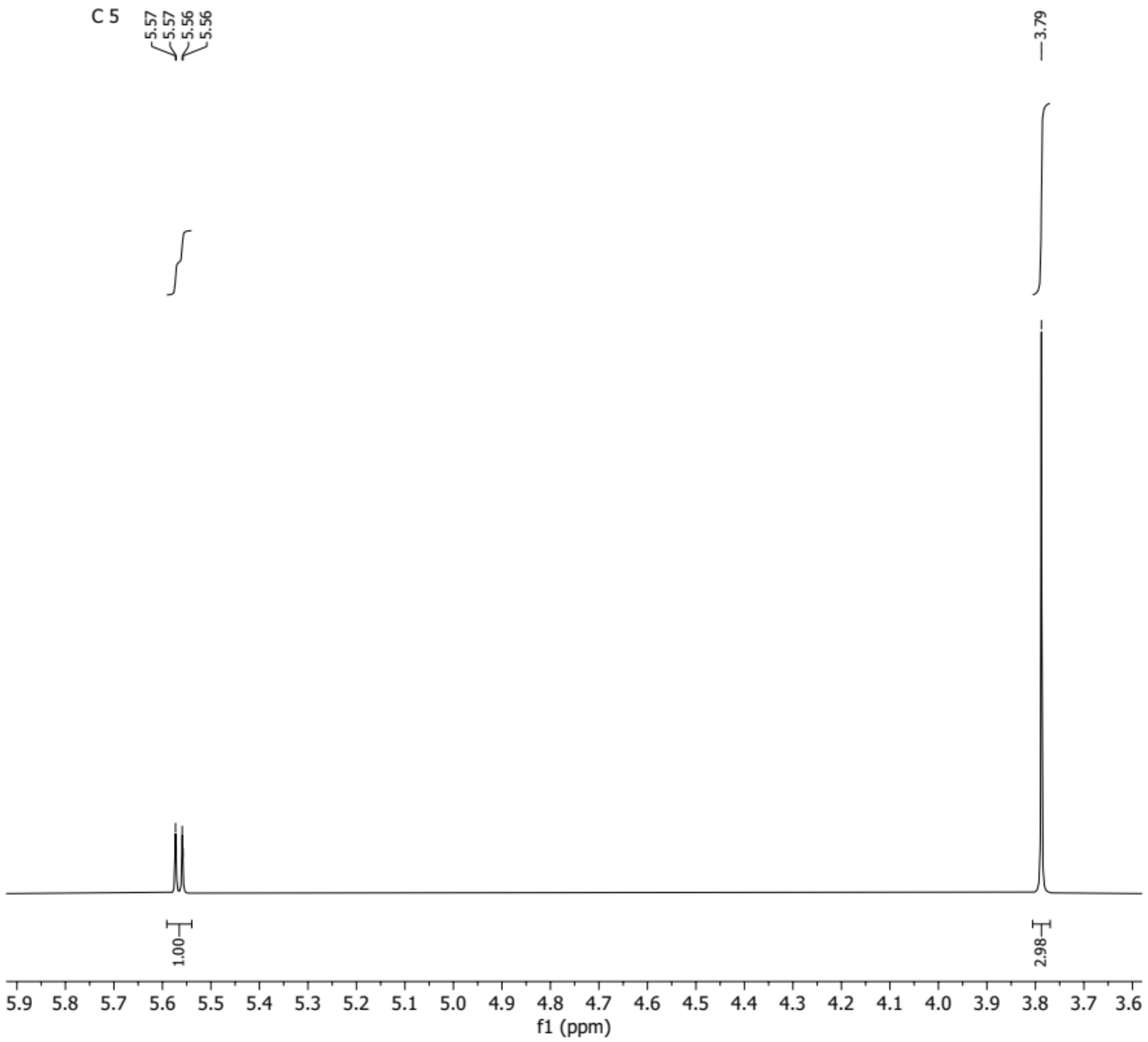

^{1}H NMR spectrum of synthesized compound C5

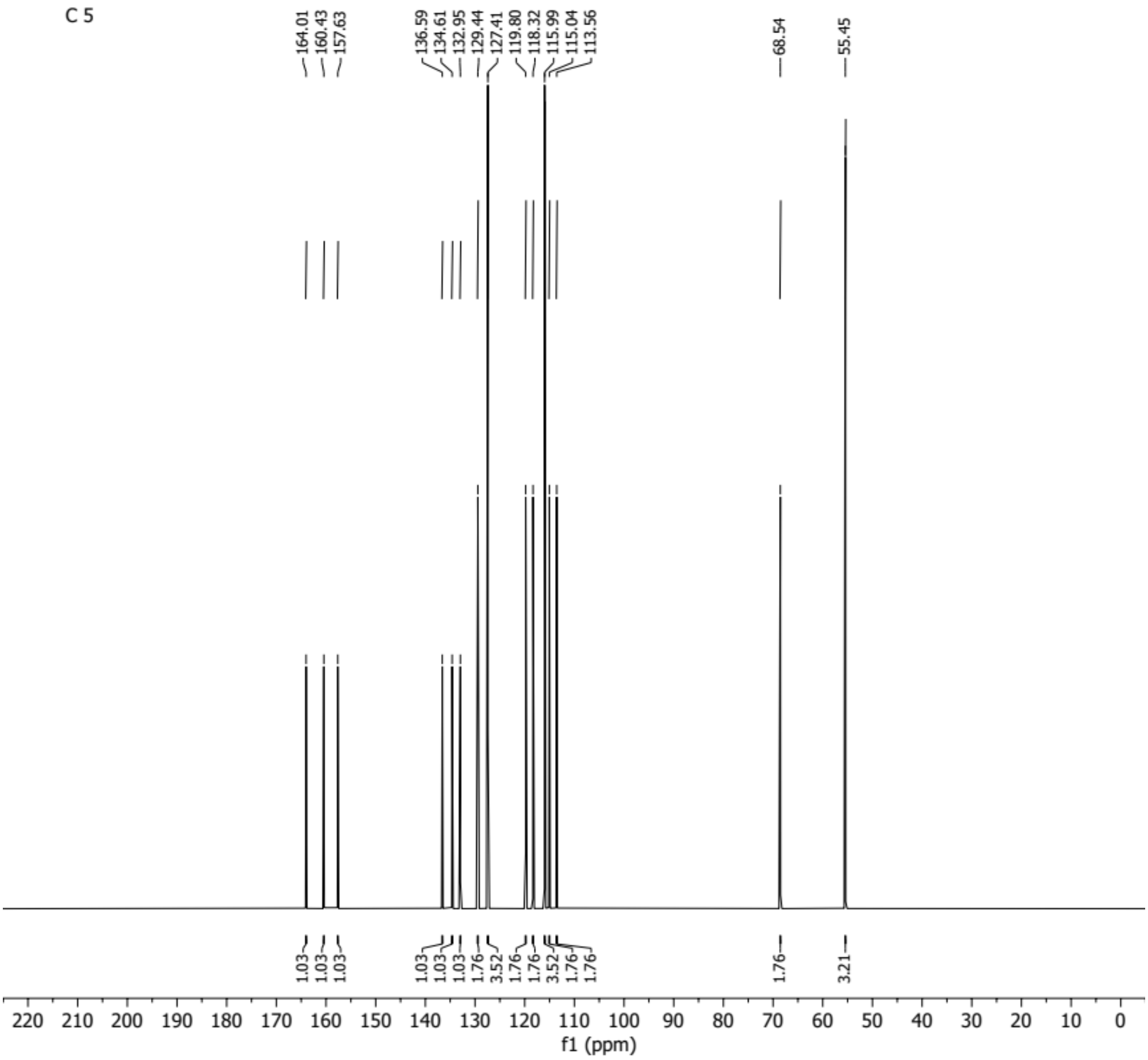

^{13}C NMR spectrum of synthesized compound C5

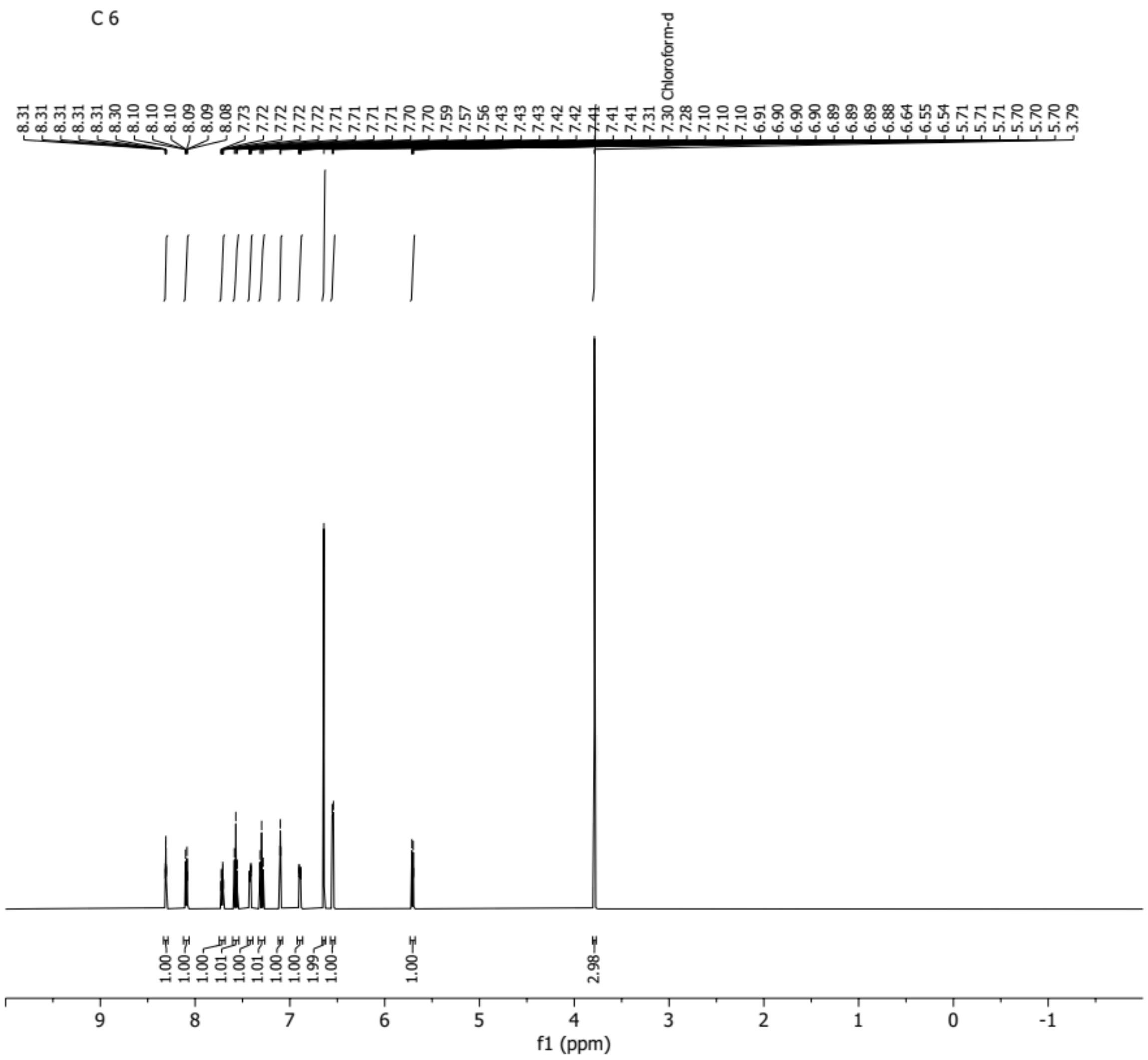
C 6
8.31
8.31
8.31
8.31
8.30
8.10
8.10
8.09
8.09
8.08
7.73
7.72
7.72
7.72
7.72
7.71
7.71
7.71
7.71
7.70
7.70
7.59
7.57
7.56
7.43
7.43
7.43
7.42
7.42
7.41
7.41
7.41
7.31
7.30 Chloroform-d
7.28
7.10
7.10
7.10
6.91
6.90
6.90
6.90
6.89
6.89
6.89
6.88
6.64
6.55
6.54
5.71
5.71
5.71
5.70
5.70
5.70
3.79
1.00
1.00
1.00
1.01
1.00
1.01
1.00
1.00
1.99
1.00
1.00
2.98
9
8
7
6
5
4
3
2
1
0
-1
f1 (ppm)

C 6

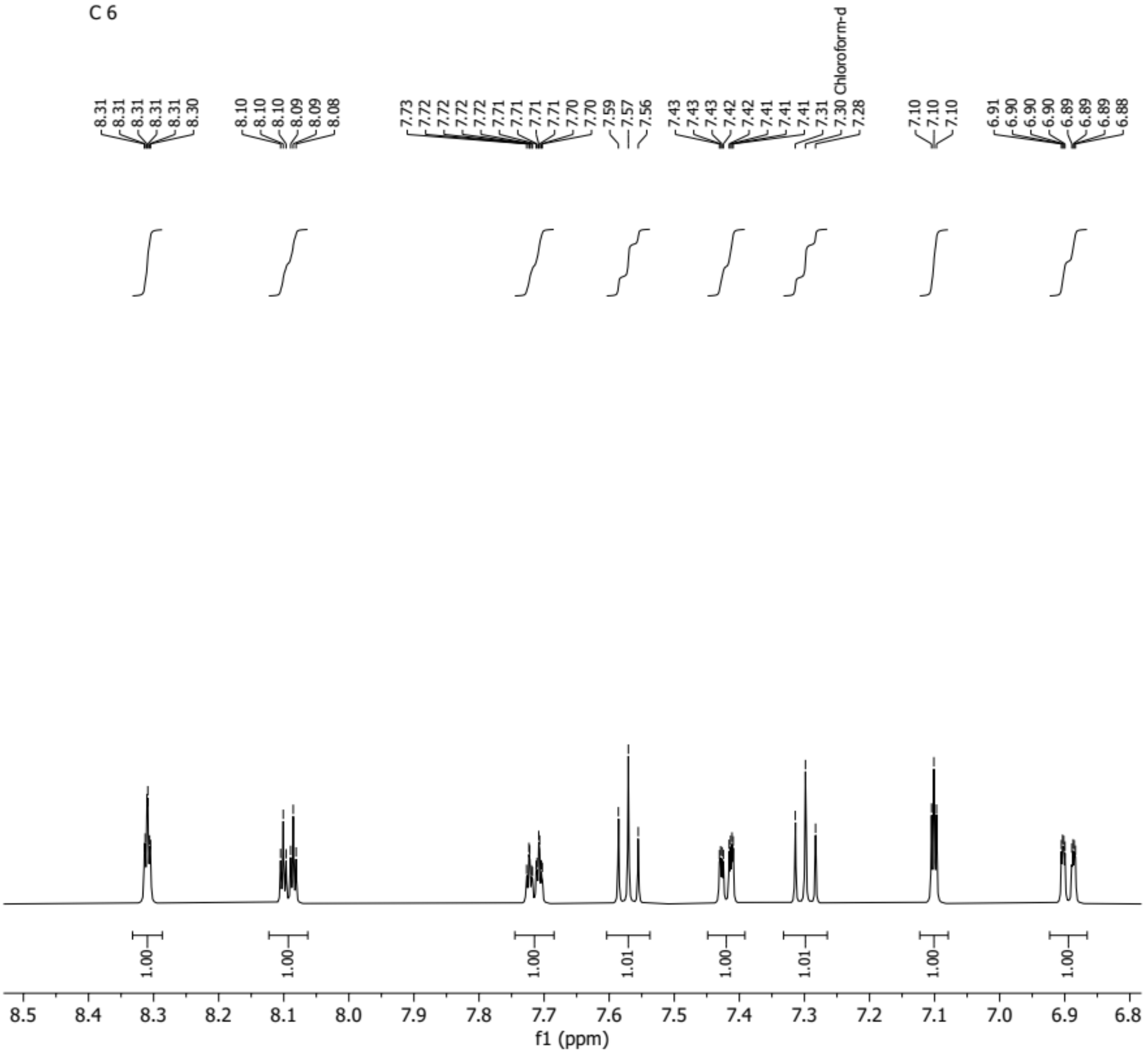

8.31
8.31
8.31
8.31
8.31
8.30
8.10
8.10
8.10
8.09
8.09
8.08
7.73
7.72
7.72
7.72
7.72
7.71
7.71
7.71
7.71
7.70
7.59
7.57
7.56
7.43
7.43
7.43
7.42
7.42
7.41
7.41
7.41
7.31
7.30 Chloroform-d
7.28
7.10
7.10
7.10
6.91
6.90
6.90
6.90
6.89
6.89
6.89
6.88
1.00
1.00
1.00
1.01
1.00
1.01
1.00
1.00
8.5
8.4
8.3
8.2
8.1
8.0
7.9
7.8
7.7
7.6
7.5
7.4
7.3
7.2
7.1
7.0
6.9
6.8
f1 (ppm)

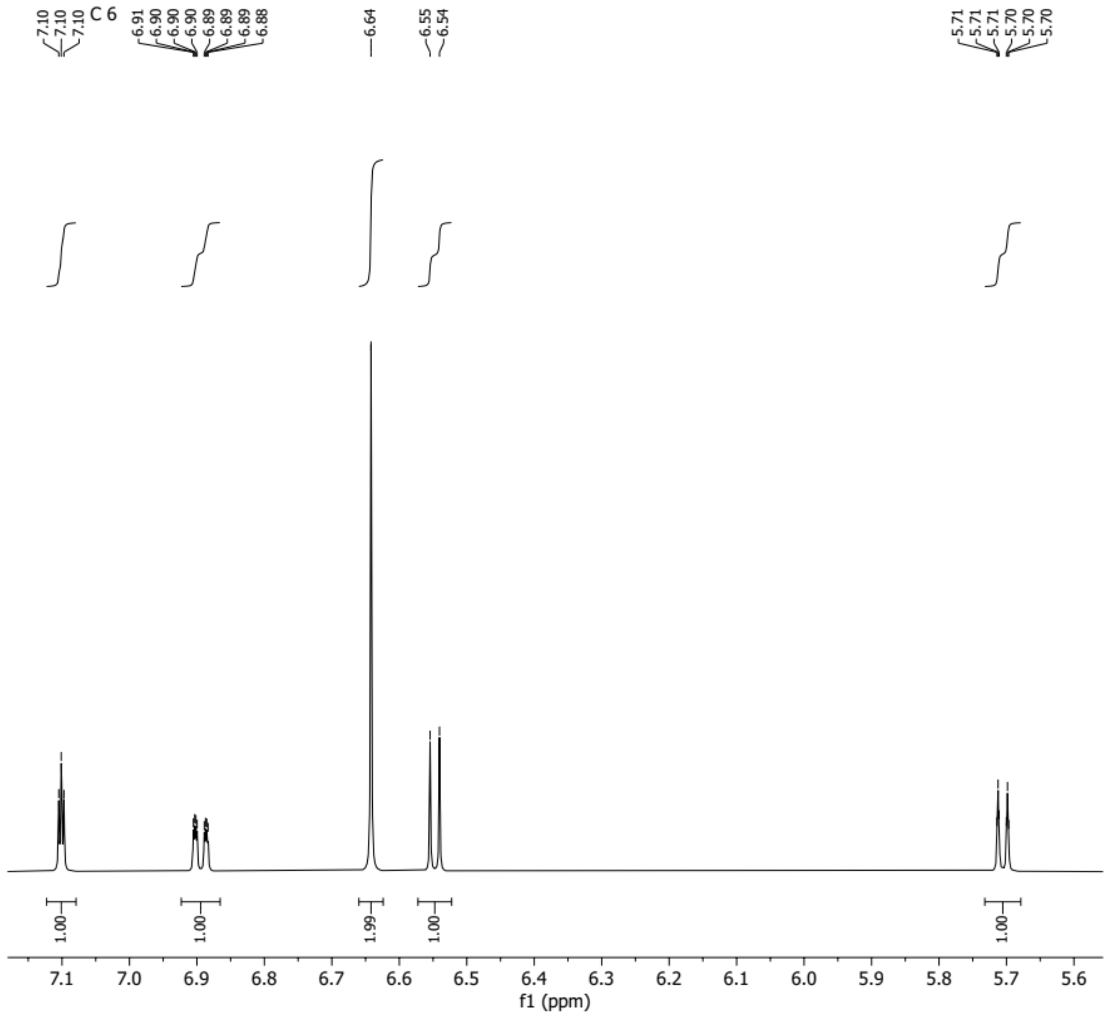

^{1}H NMR spectrum of synthesized compound C6

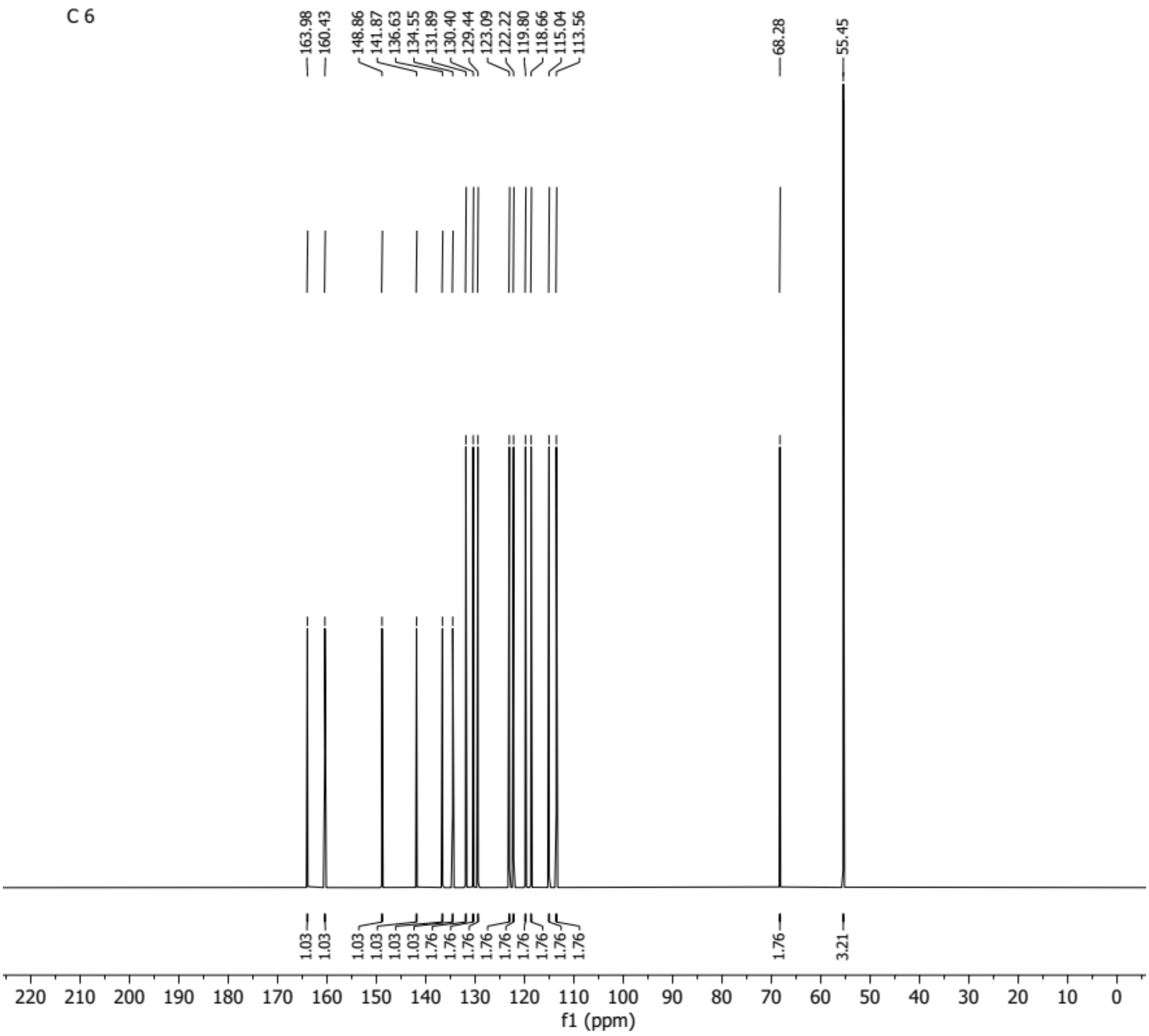

^{13}C NMR spectrum of synthesized compound C6

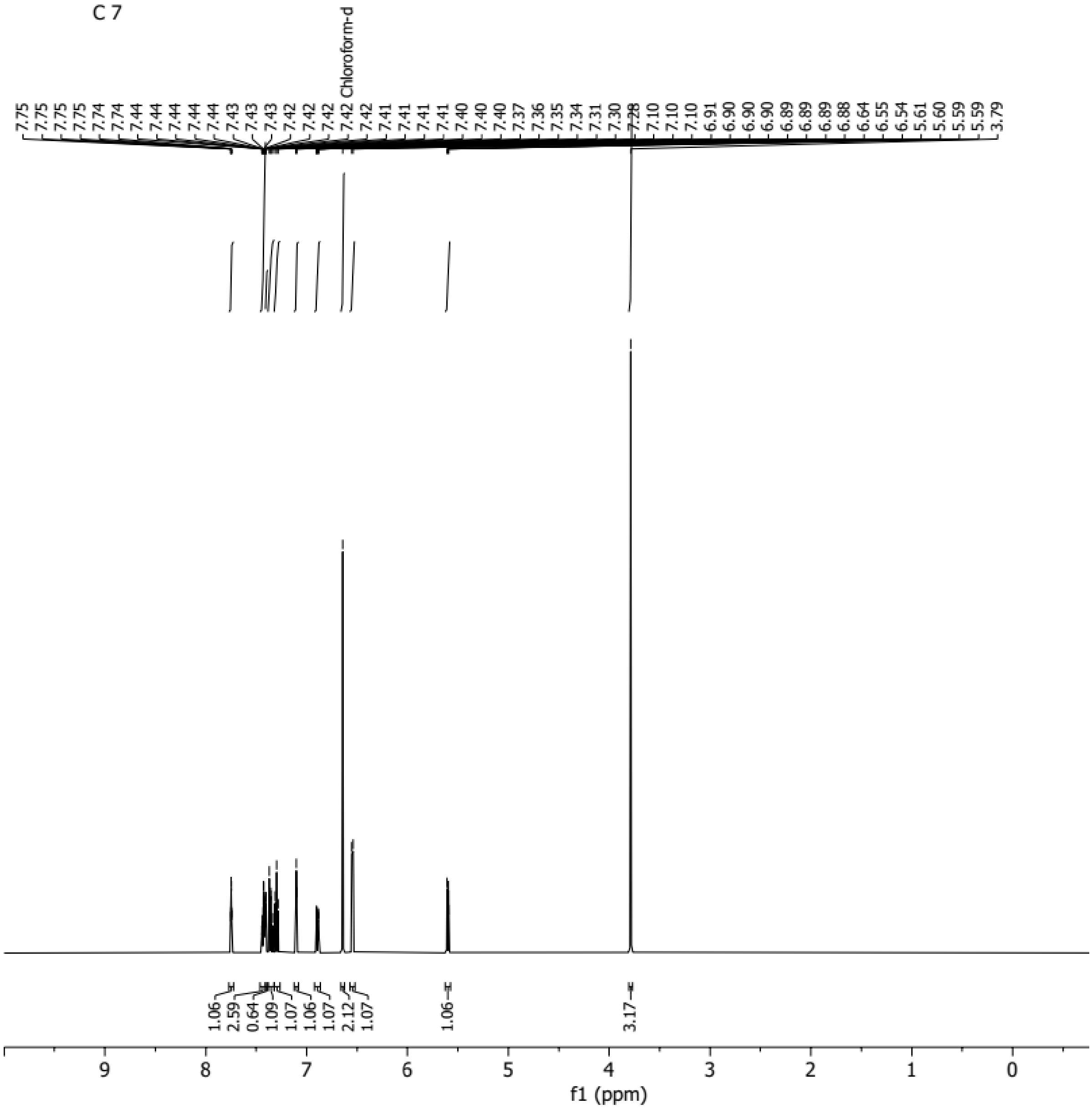
C 7
Chloroform-d
7.75
7.75
7.75
7.75
7.74
7.74
7.44
7.44
7.44
7.44
7.44
7.43
7.43
7.43
7.42
7.42
7.42
7.42
7.42
7.41
7.41
7.41
7.41
7.40
7.40
7.40
7.37
7.36
7.35
7.34
7.31
7.30
7.28
7.10
7.10
7.10
6.91
6.90
6.90
6.89
6.89
6.89
6.88
6.64
6.55
6.54
5.61
5.60
5.59
5.59
3.79
1.06
2.59
0.64
1.09
1.07
1.06
1.07
2.12
1.07
1.06
3.17
9
8
7
6
5
4
3
2
1
0
f1 (ppm)

C 7

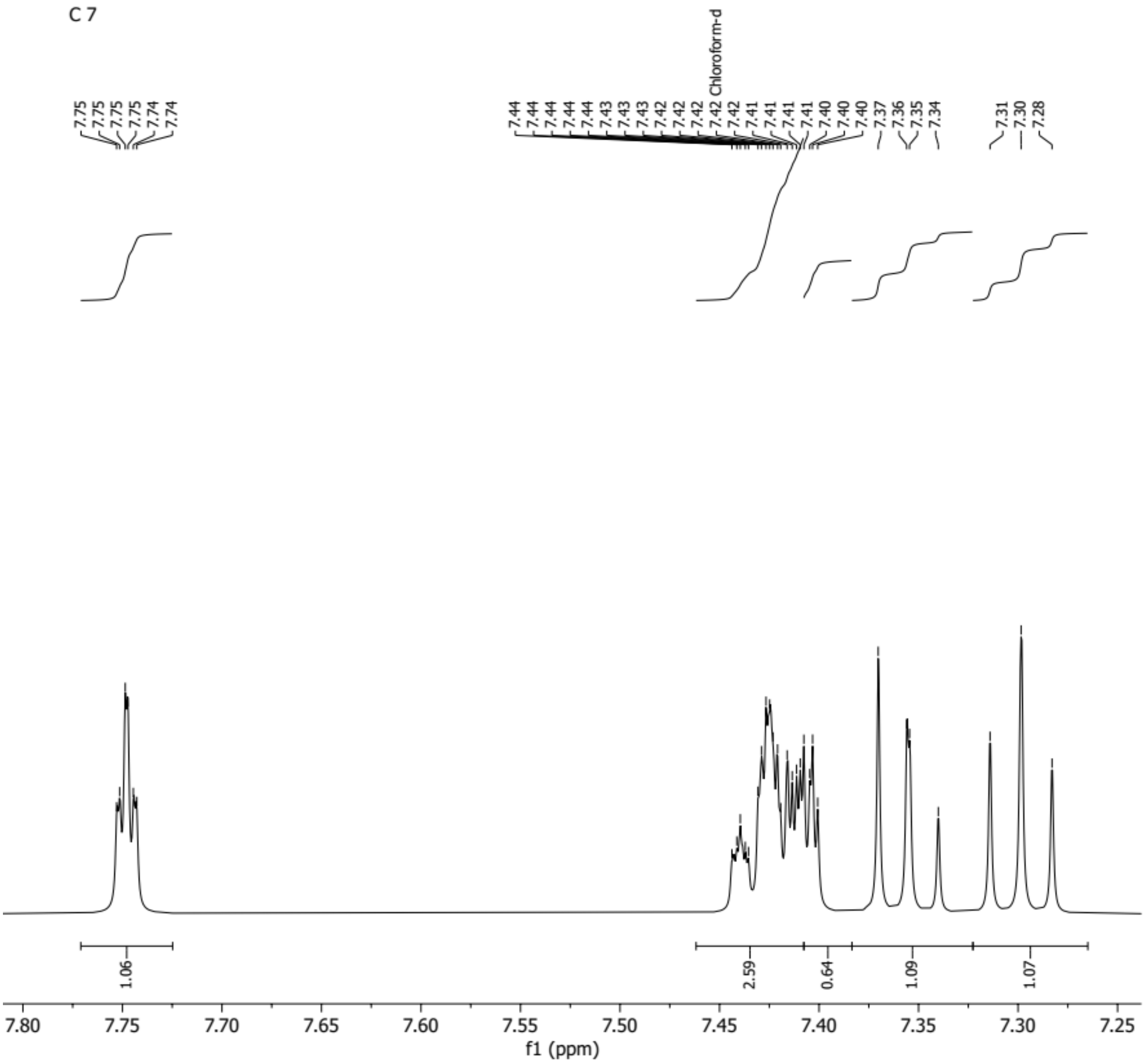
7.75
7.75
7.75
7.75
7.74
7.74
7.44
7.44
7.44
7.44
7.44
7.43
7.43
7.43
7.42
7.42
7.42
Chloroform-d
7.42
7.41
7.41
7.41
7.41
7.40
7.40
7.40
7.37
7.36
7.35
7.34
7.31
7.30
7.28
1.06
2.59
0.64
1.09
1.07
7.80
7.75
7.70
7.65
7.60
7.55
7.50
7.45
7.40
7.35
7.30
7.25
f1 (ppm)

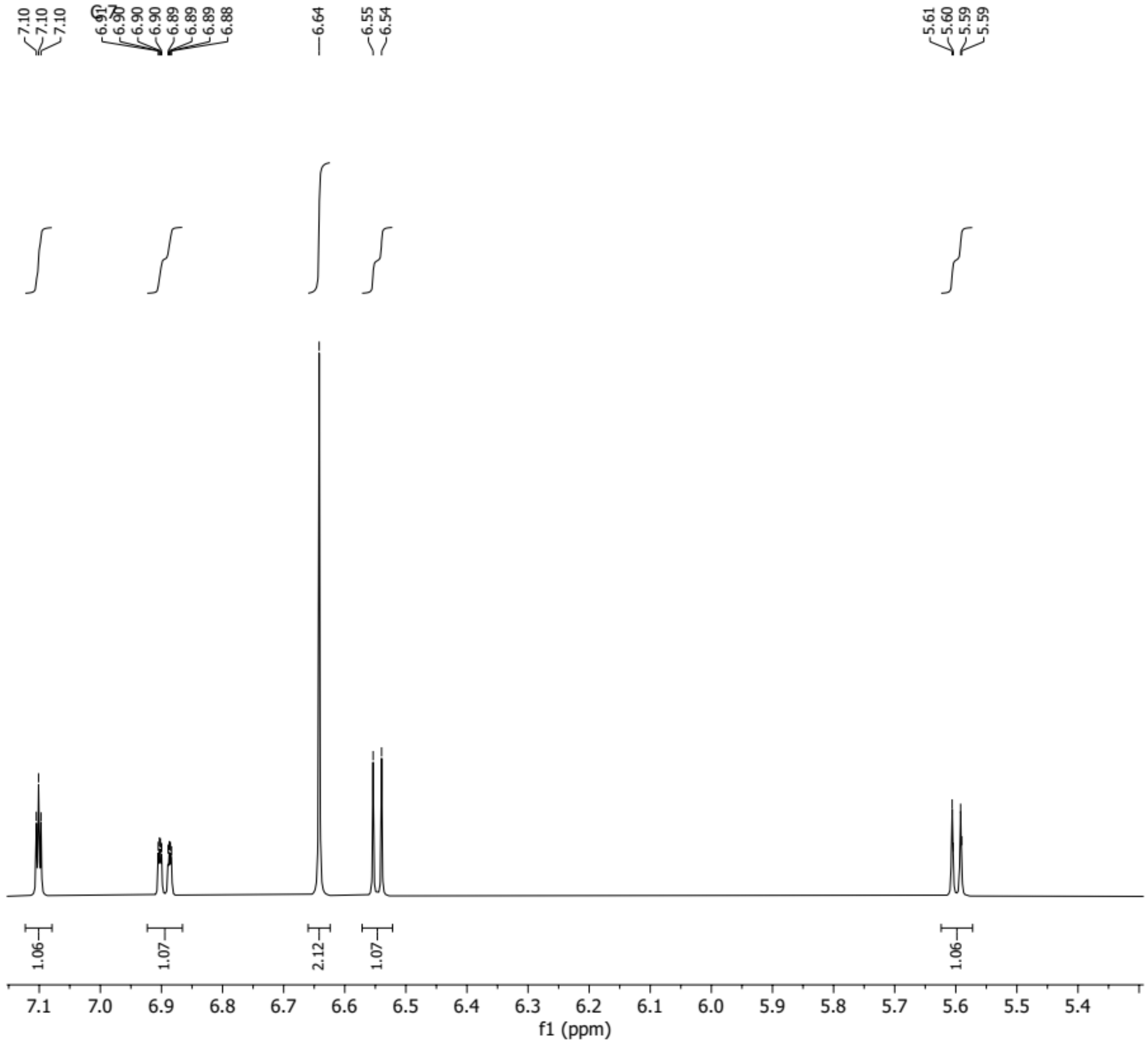

^{1}H NMR spectrum of synthesized compound C7

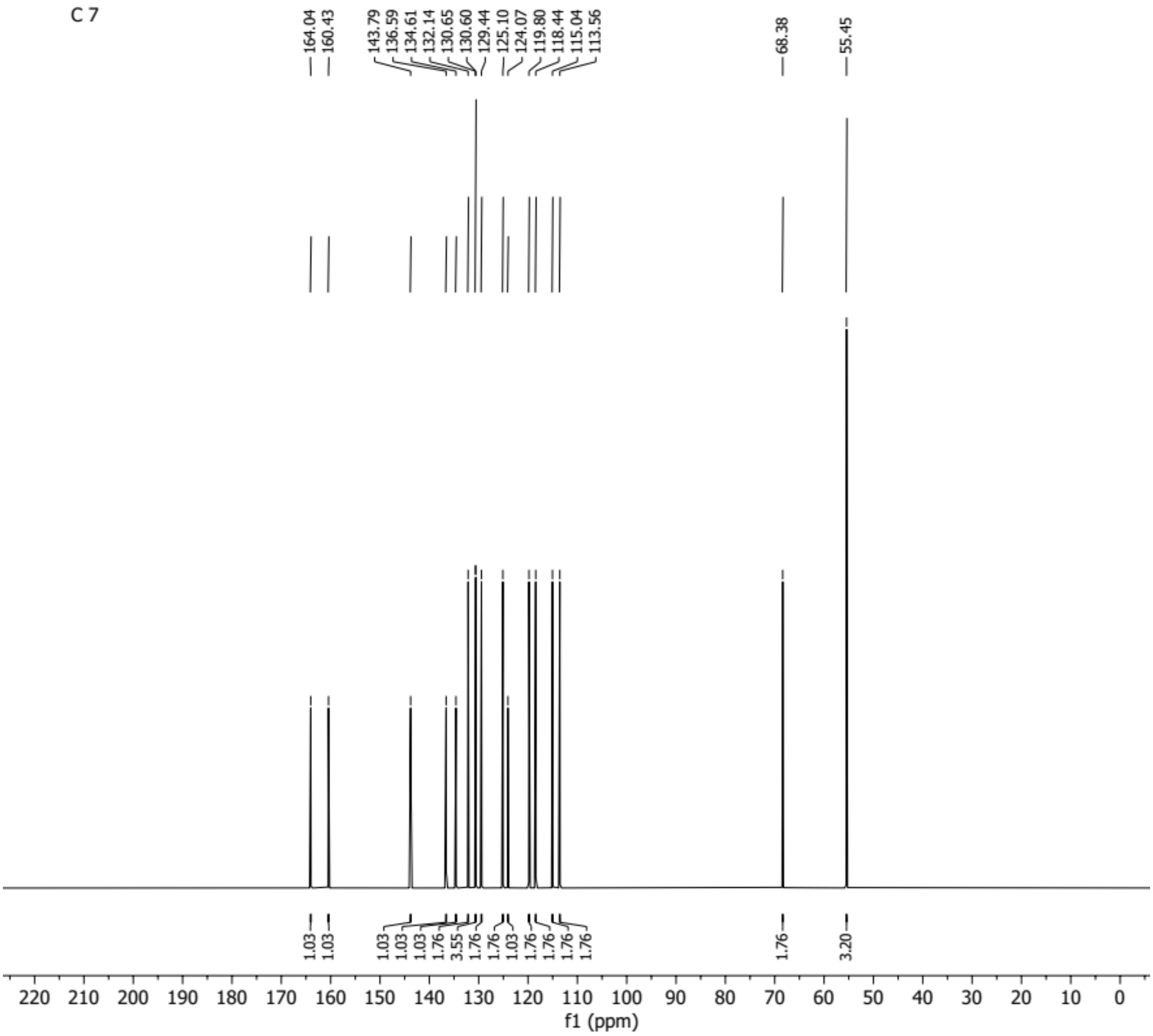

^{13}C NMR spectrum of synthesized compound C7

C 8

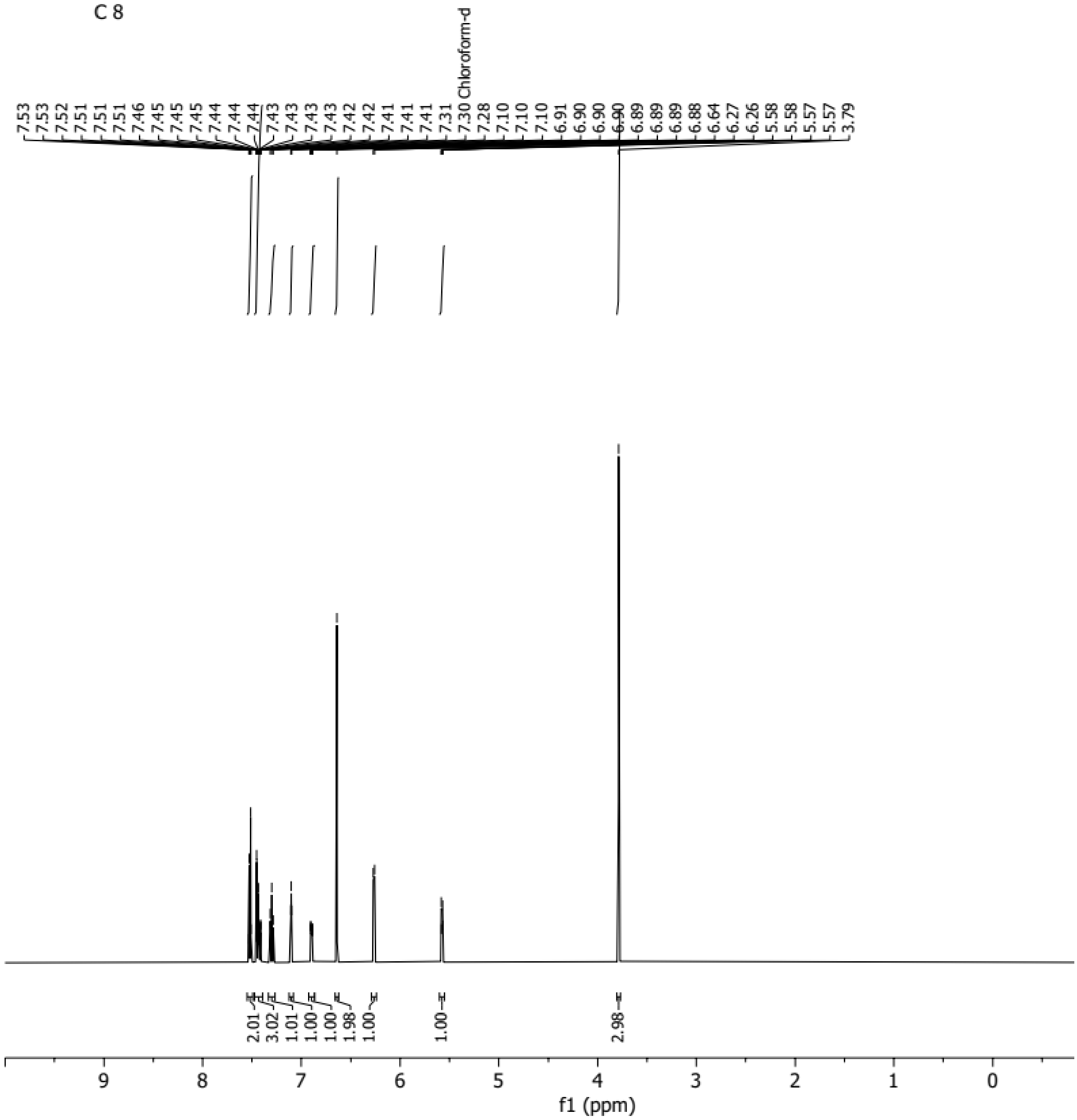

7.53
7.53
7.52
7.51
7.51
7.51
7.46
7.45
7.45
7.45
7.44
7.44
7.43
7.43
7.43
7.43
7.42
7.42
7.41
7.41
7.41
7.31
7.30
Chloroform-d
7.28
7.10
7.10
7.10
6.91
6.90
6.90
6.90
6.89
6.89
6.88
6.64
6.27
6.26
5.58
5.57
5.57
3.79
2.01
3.02
1.01
1.00
1.00
1.98
1.00
1.00
2.98
9
8
7
6
5
4
3
2
1
0
f1 (ppm)

C 8

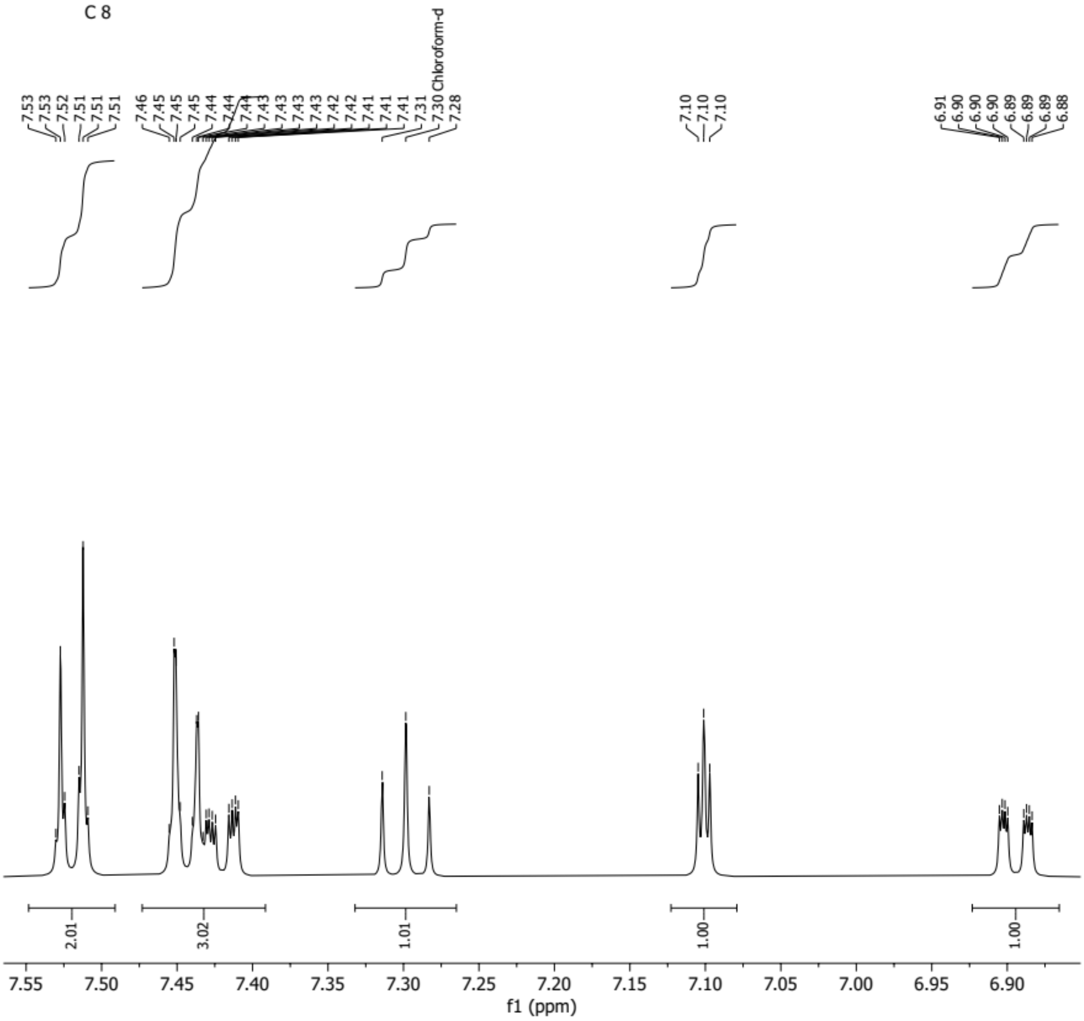
7.53
7.53
7.52
7.51
7.51
7.51
7.46
7.45
7.45
7.45
7.44
7.44
7.43
7.43
7.43
7.42
7.42
7.41
7.41
7.41
7.31
7.30 Chloroform-d
7.28
7.10
7.10
7.10
6.91
6.90
6.90
6.90
6.89
6.89
6.89
6.88
2.01
3.02
1.01
1.00
1.00
7.55
7.50
7.45
7.40
7.35
7.30
7.25
7.20
7.15
7.10
7.05
7.00
6.95
6.90
f1 (ppm)

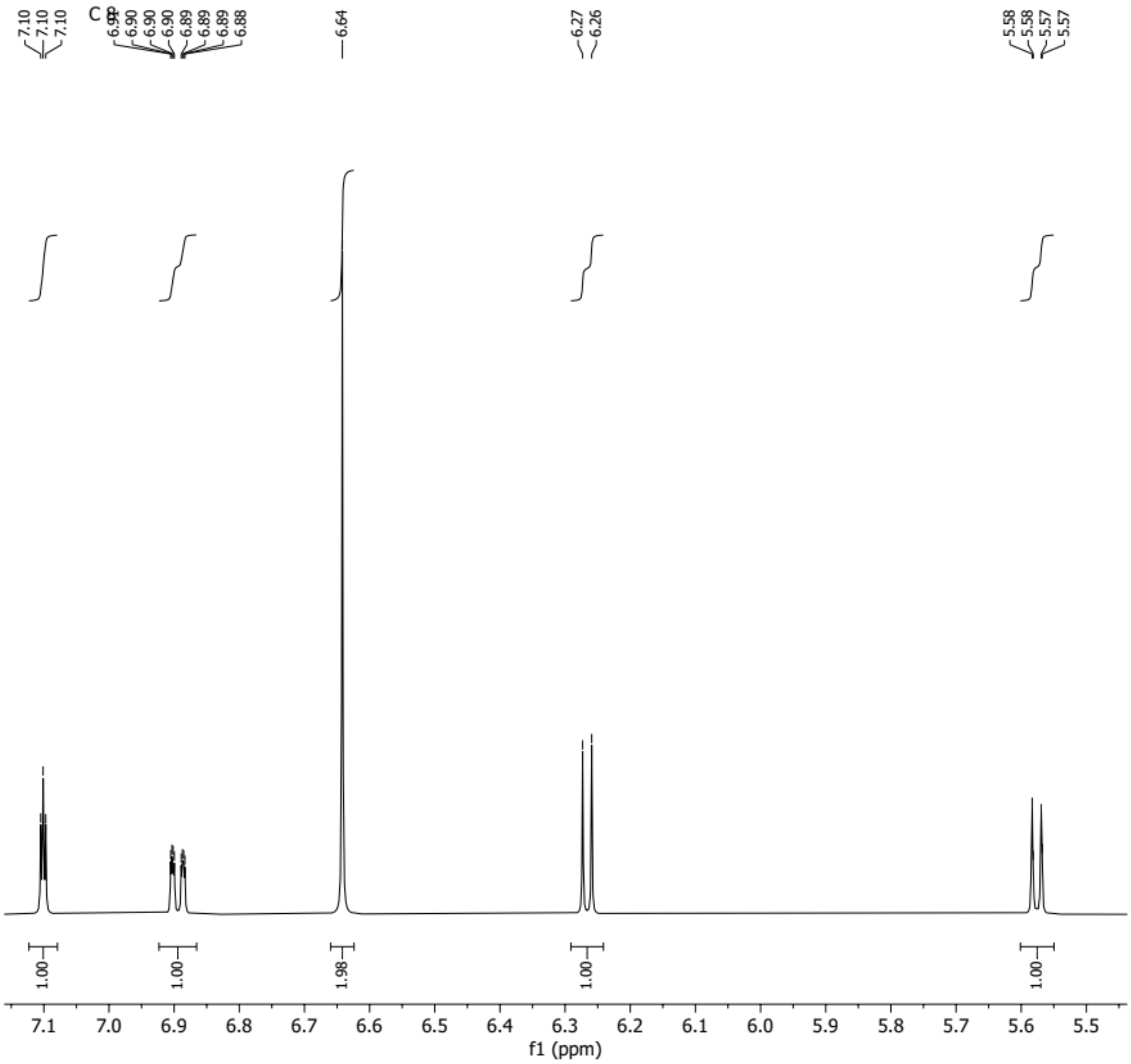
7.10
7.10
7.10
6.91
6.90
6.90
6.90
6.89
6.89
6.89
6.88
6.64
6.27
6.26
5.58
5.58
5.57
5.57
1.00
1.00
1.98
1.00
1.00
7.1
7.0
6.9
6.8
6.7
6.6
6.5
6.4
6.3
6.2
6.1
6.0
5.9
5.8
5.7
5.6
5.5
f1 (ppm)

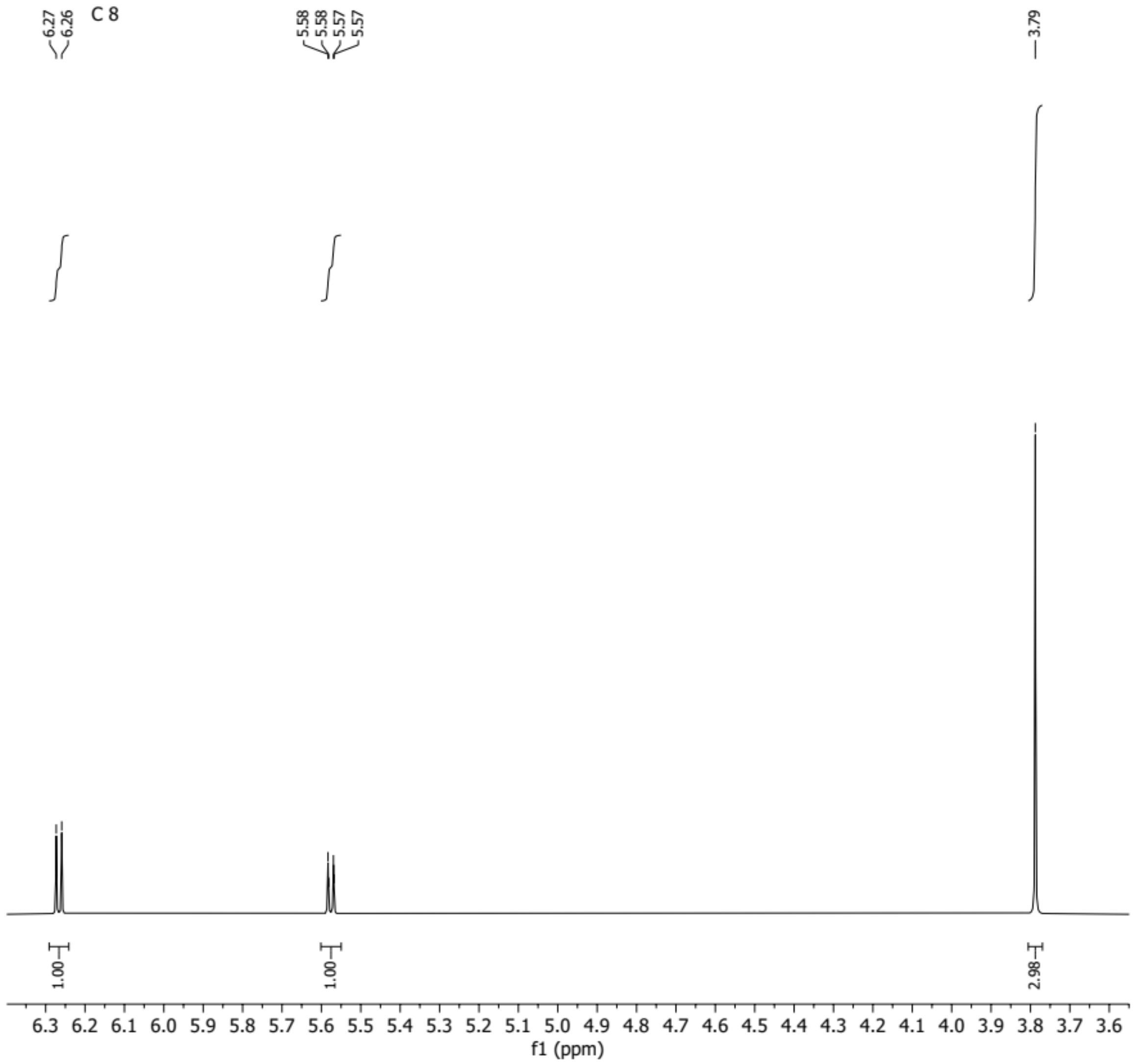

^{1}H NMR spectrum of synthesized compound C8

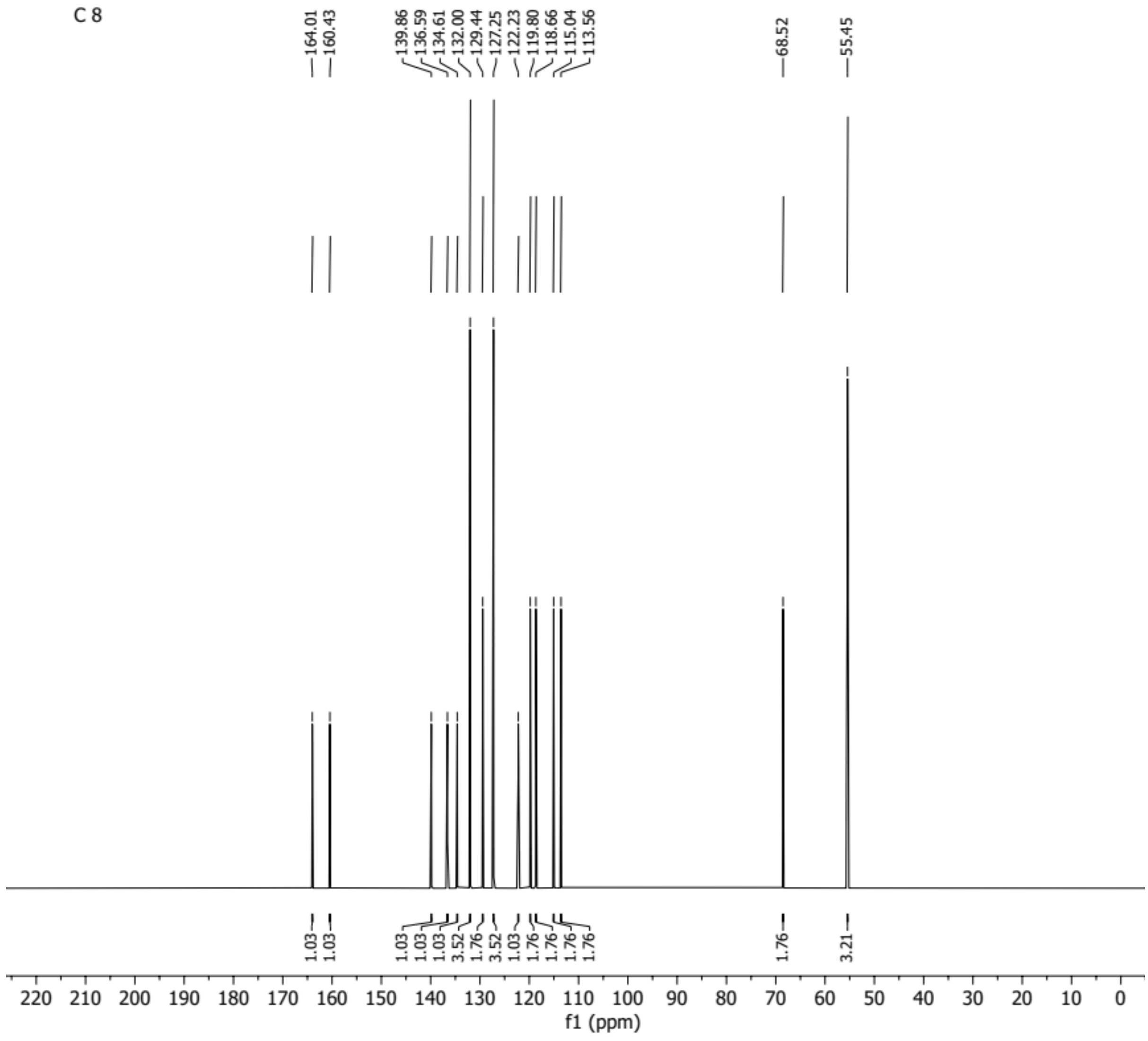

^{13}C NMR spectrum of synthesized compound C8

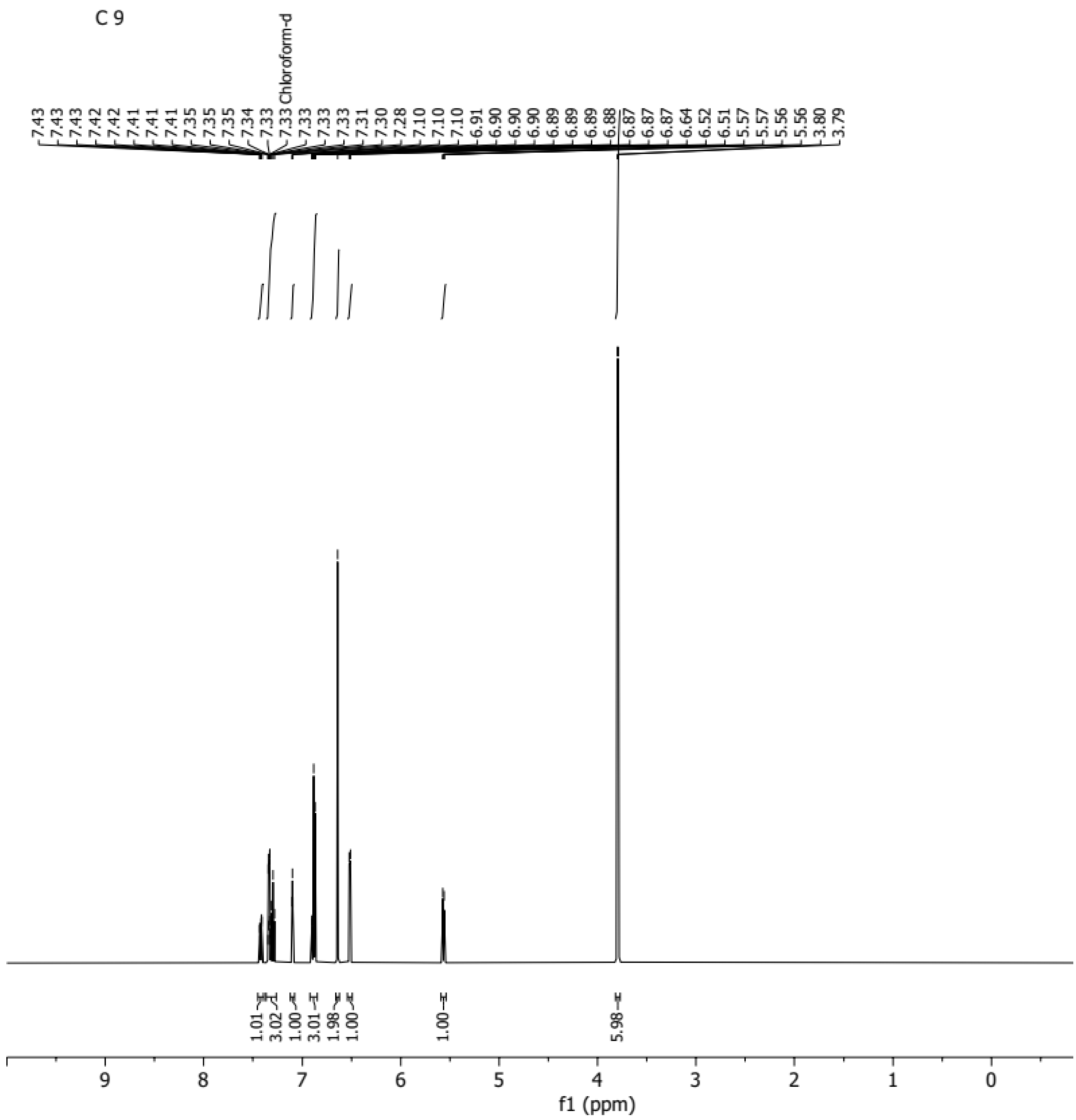
C 9
Chloroform-d
7.43
7.43
7.43
7.42
7.42
7.41
7.41
7.41
7.35
7.35
7.35
7.34
7.33
7.33
7.33
7.33
7.33
7.31
7.30
7.28
7.10
7.10
7.10
6.91
6.90
6.90
6.90
6.89
6.89
6.89
6.88
6.87
6.87
6.87
6.64
6.52
6.51
5.57
5.57
5.56
5.56
3.80
3.79
1.01
3.02
1.00
3.01
1.98
1.00
1.00
5.98
9
8
7
6
5
4
3
2
1
0
f1 (ppm)

C 9

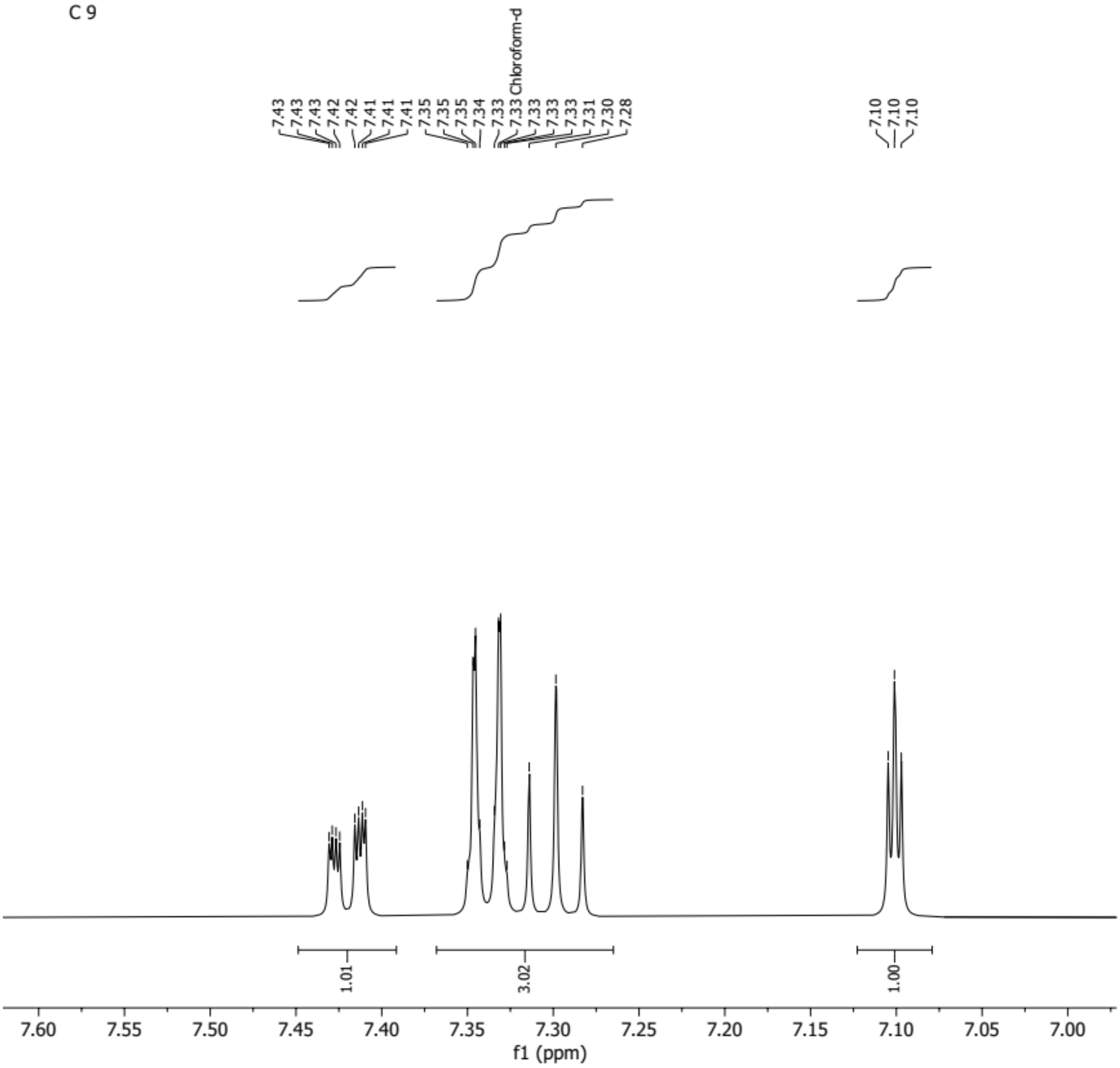
7.43
7.43
7.43
7.42
7.42
7.41
7.41
7.41
7.35
7.35
7.35
7.34
7.33
7.33 Chloroform-d
7.33
7.33
7.33
7.31
7.30
7.28
7.10
7.10
7.10
1.01
3.02
1.00
7.60
7.55
7.50
7.45
7.40
7.35
7.30
7.25
7.20
7.15
7.10
7.05
7.00
f1 (ppm)

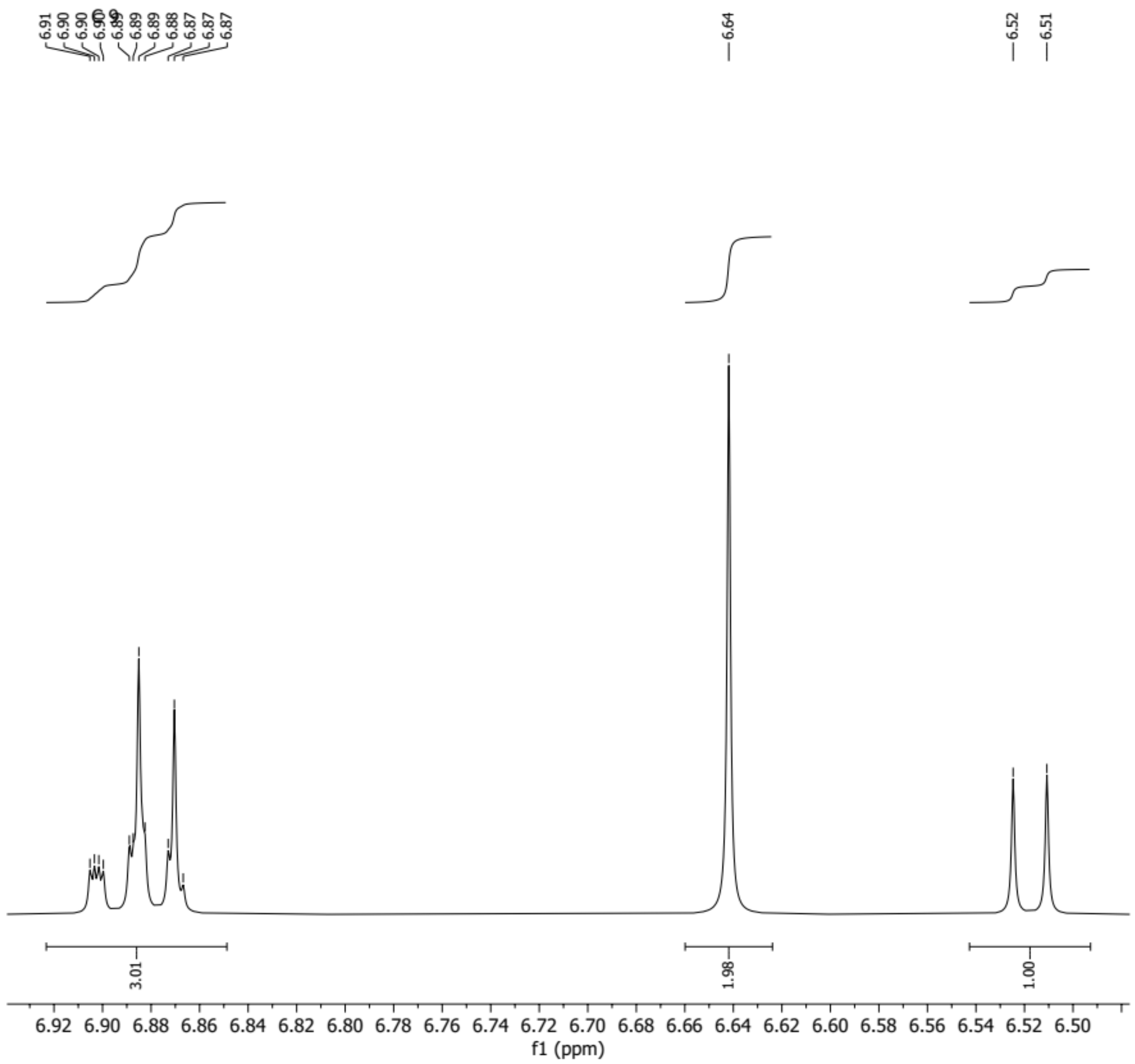

6.91
6.90
6.90
6.90
6.89
6.89
6.89
6.88
6.87
6.87
6.87
6.64
6.52
6.51
3.01
1.98
1.00
6.92 6.90 6.88 6.86 6.84 6.82 6.80 6.78 6.76 6.74 6.72 6.70 6.68 6.66 6.64 6.62 6.60 6.58 6.56 6.54 6.52 6.50
f1 (ppm)

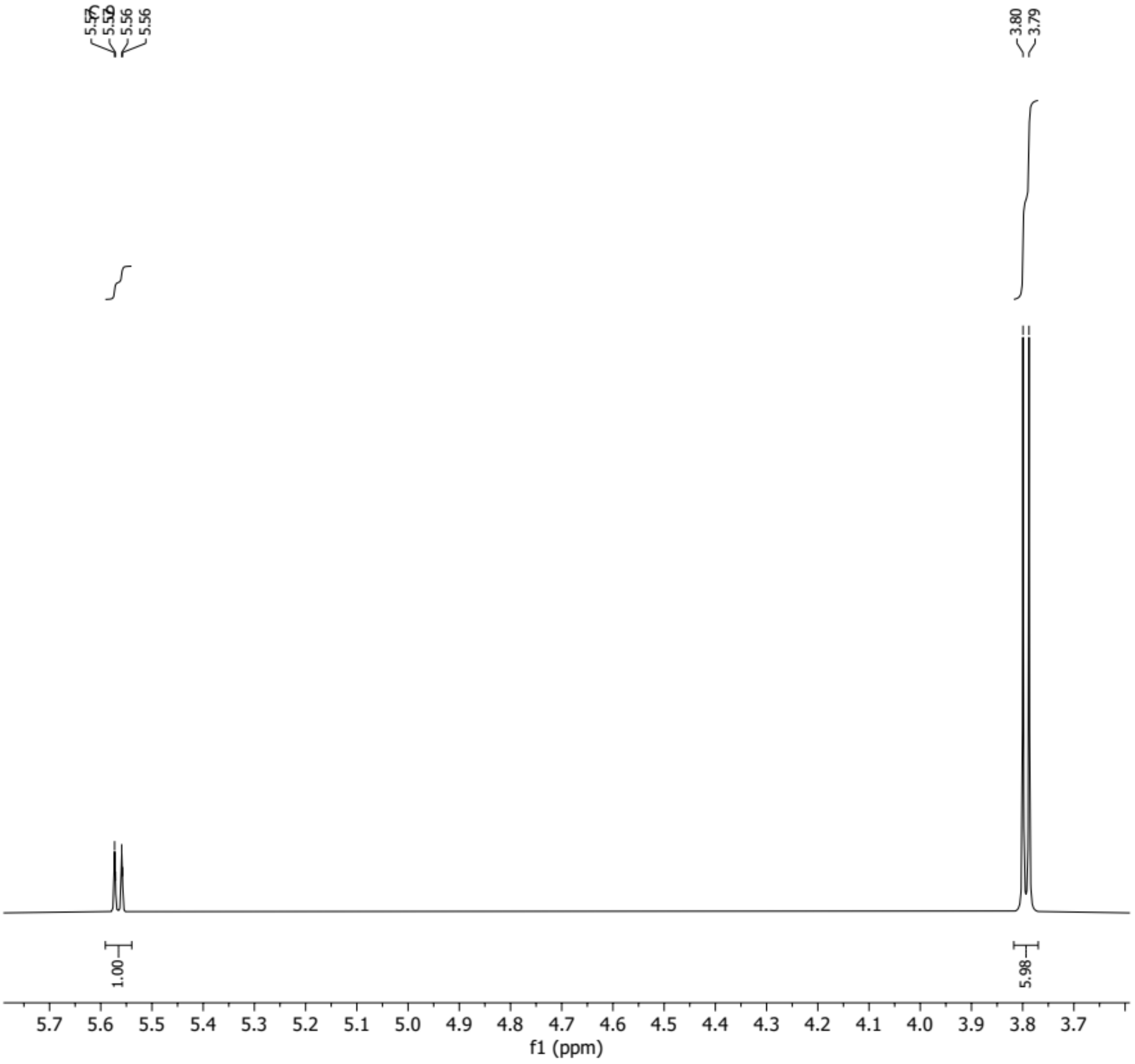

^{1}H NMR spectrum of synthesized compound C9

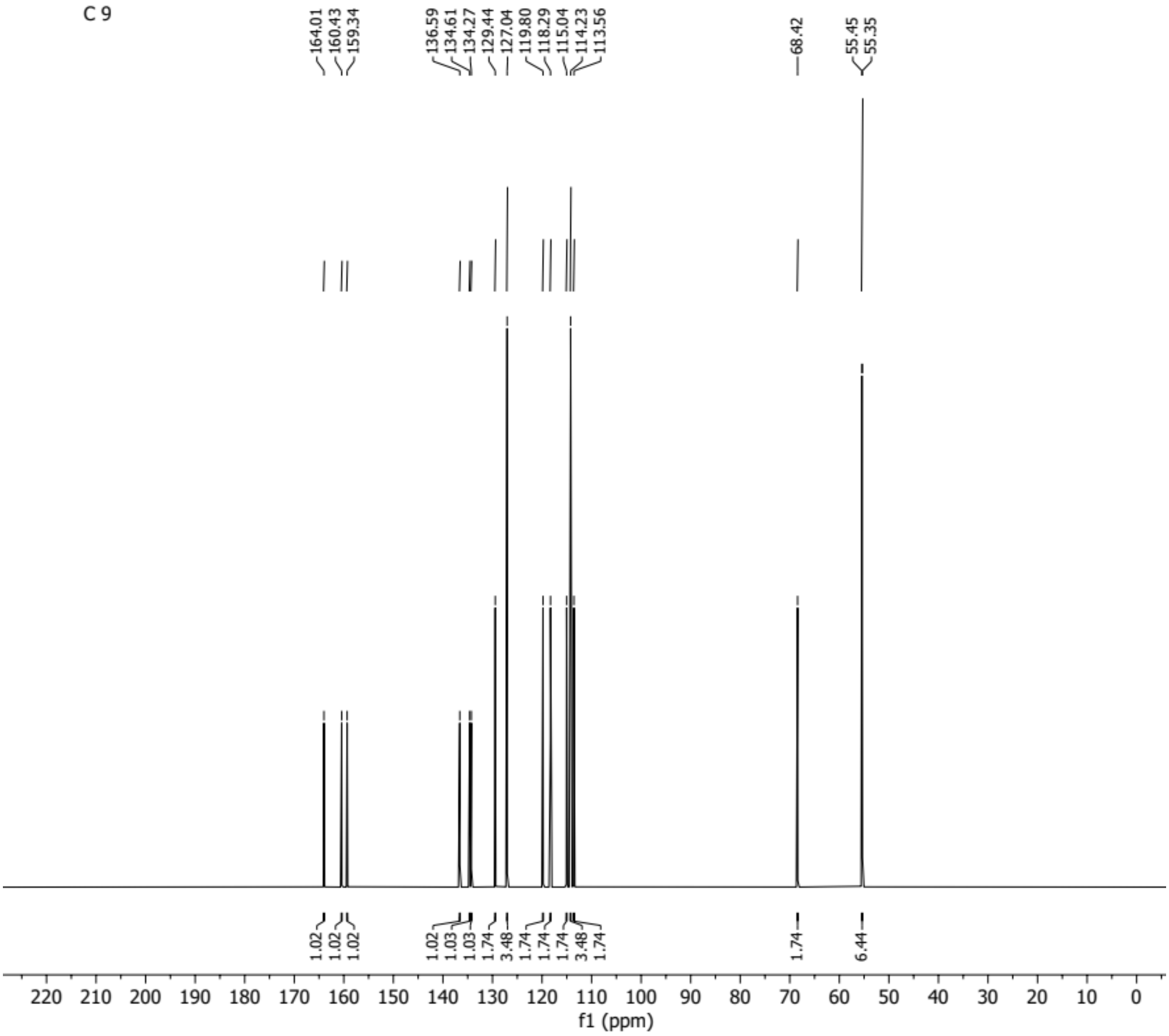

^{13}C NMR spectrum of synthesized compound C9

C 10

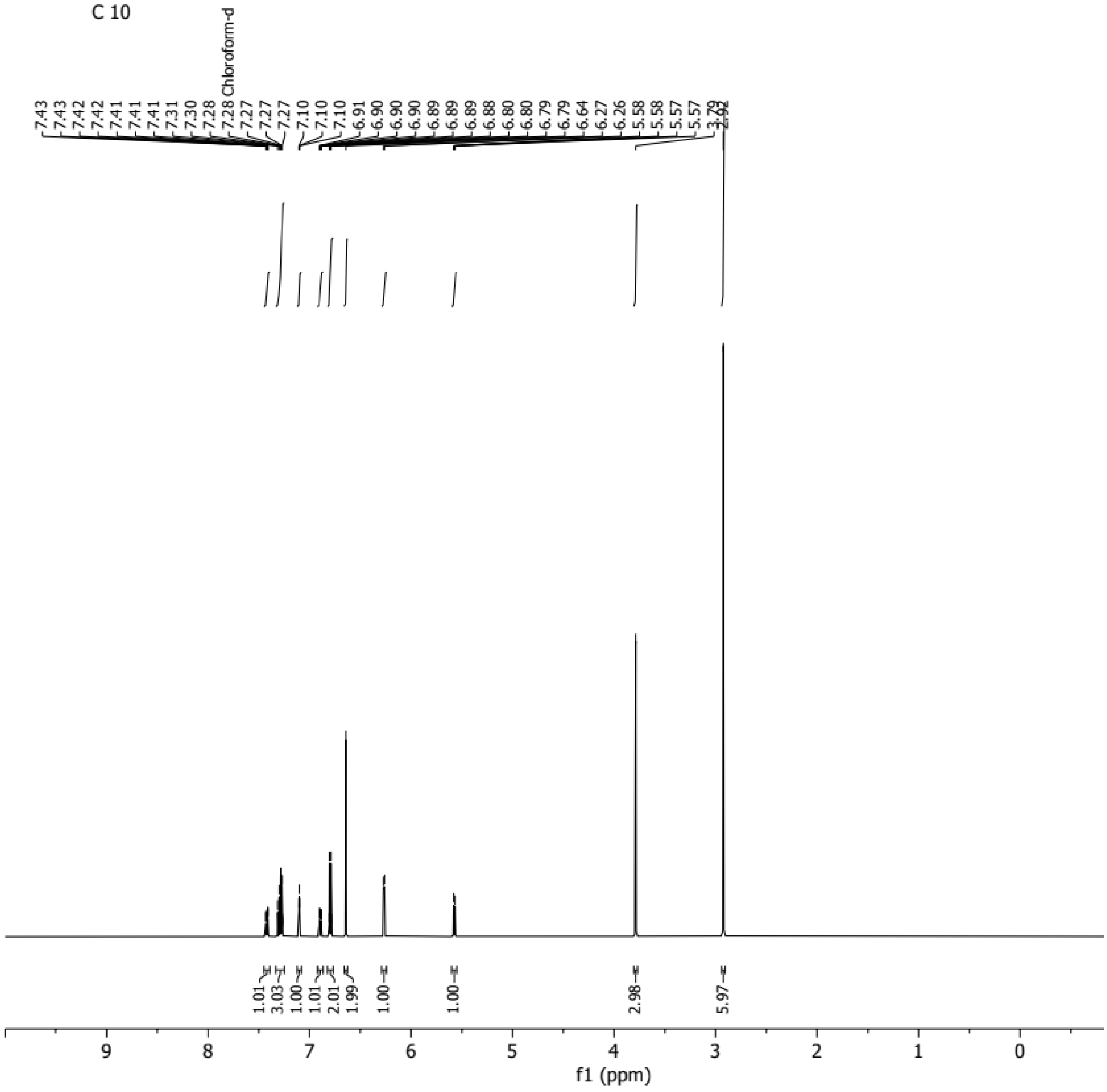

7.43
7.43
7.42
7.42
7.41
7.41
7.41
7.31
7.30
7.28
7.28 Chloroform-d
7.27
7.27
7.27
7.10
7.10
7.10
6.91
6.90
6.90
6.90
6.89
6.89
6.89
6.88
6.80
6.80
6.79
6.64
6.27
6.26
5.58
5.58
5.57
5.57
3.79
2.92
1.01
3.03
1.00
1.01
2.01
1.99
1.00
1.00
2.98
5.97
9
8
7
6
5
4
3
2
1
0
f1 (ppm)

C 10

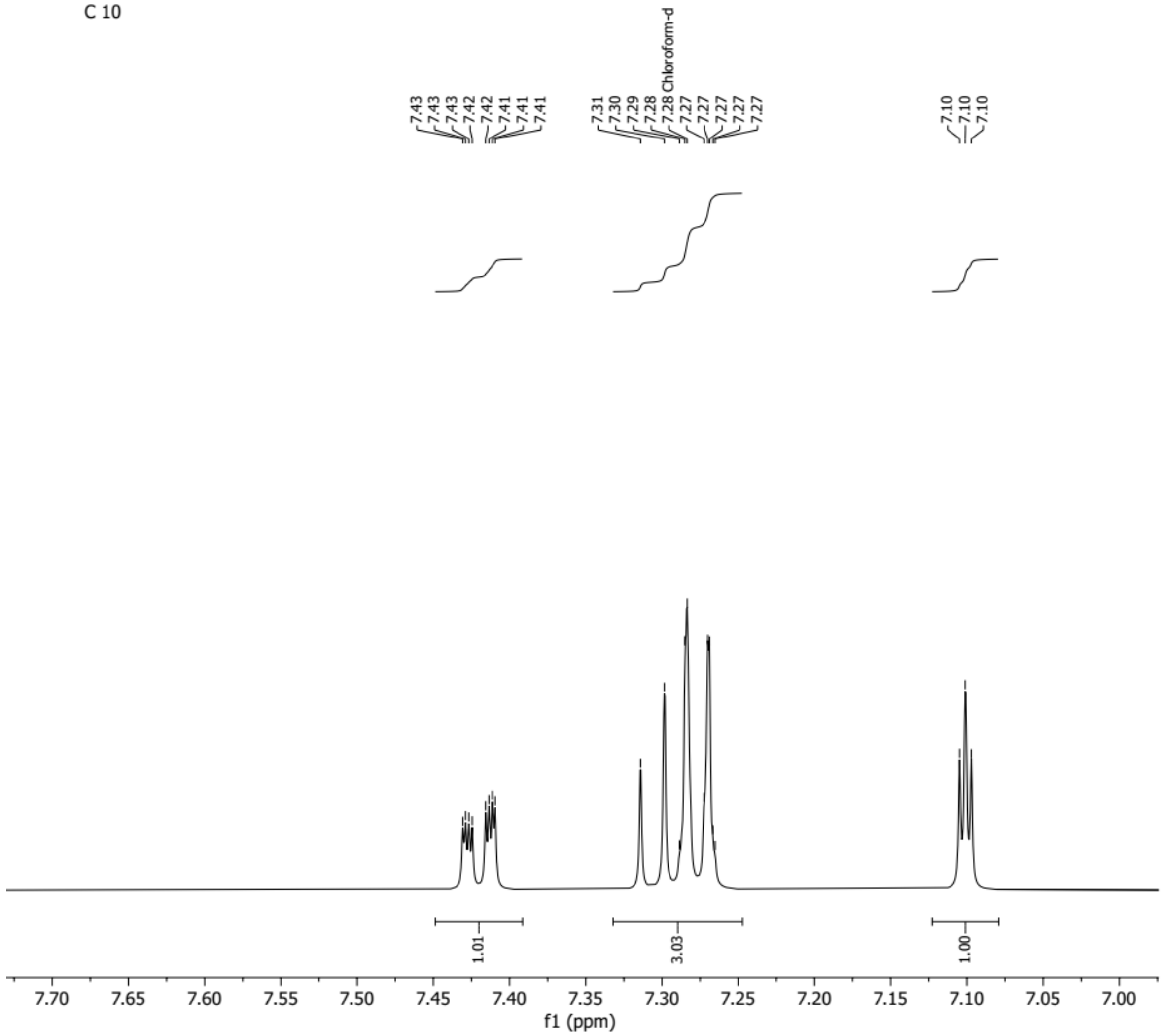

Chloroform-d
7.43
7.43
7.43
7.42
7.42
7.41
7.41
7.41
7.31
7.30
7.29
7.28
7.28
7.27
7.27
7.27
7.27
7.27
7.10
7.10
7.10
1.01
3.03
1.00
7.70
7.65
7.60
7.55
7.50
7.45
7.40
7.35
7.30
7.25
7.20
7.15
7.10
7.05
7.00
f1 (ppm)

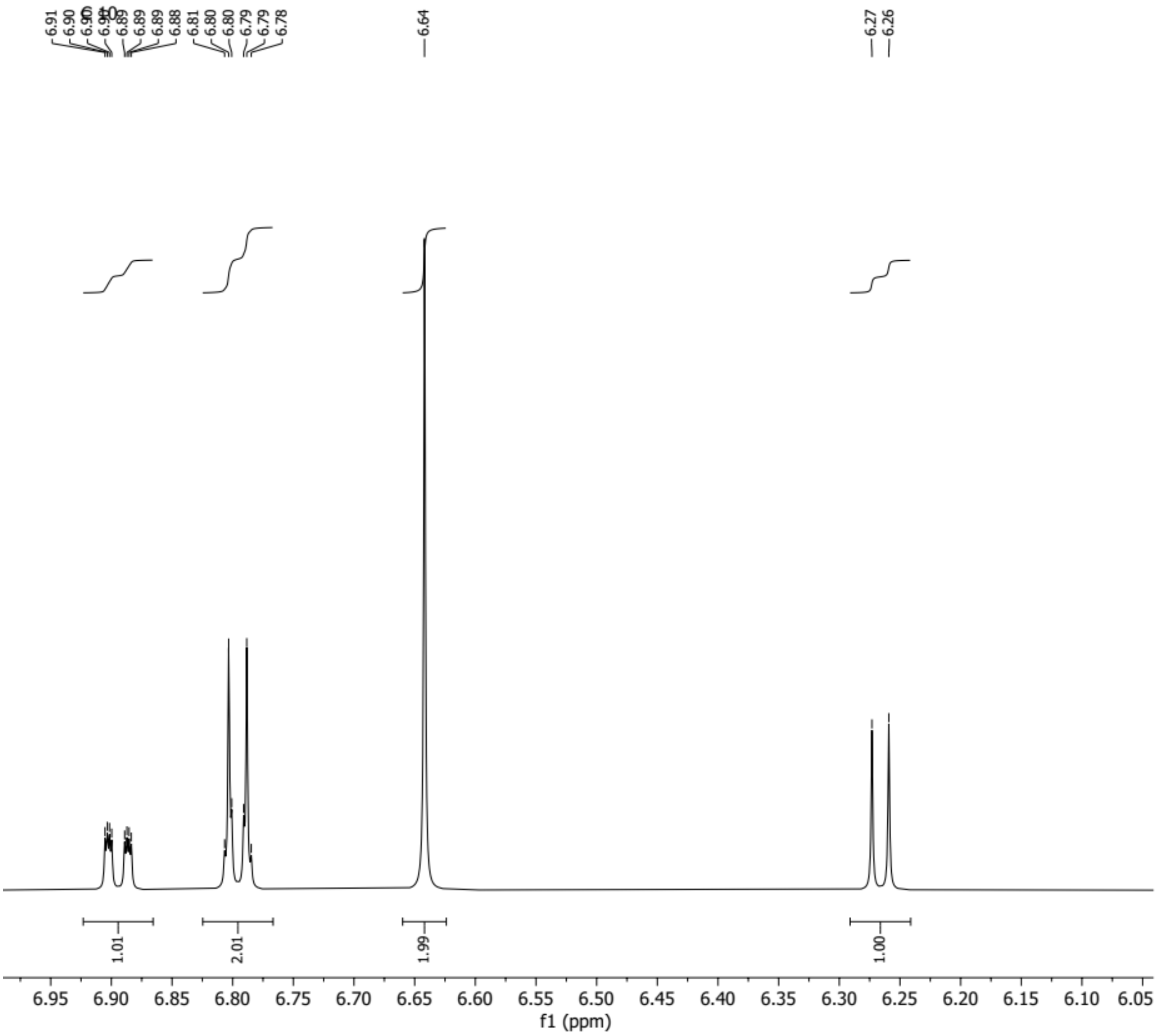
6.91
6.90
6.90
6.90
6.89
6.89
6.89
6.88
6.81
6.80
6.80
6.79
6.79
6.78
6.64
6.27
6.26
1.01
2.01
1.99
1.00
6.95 6.90 6.85 6.80 6.75 6.70 6.65 6.60 6.55 6.50 6.45 6.40 6.35 6.30 6.25 6.20 6.15 6.10 6.05
f1 (ppm)

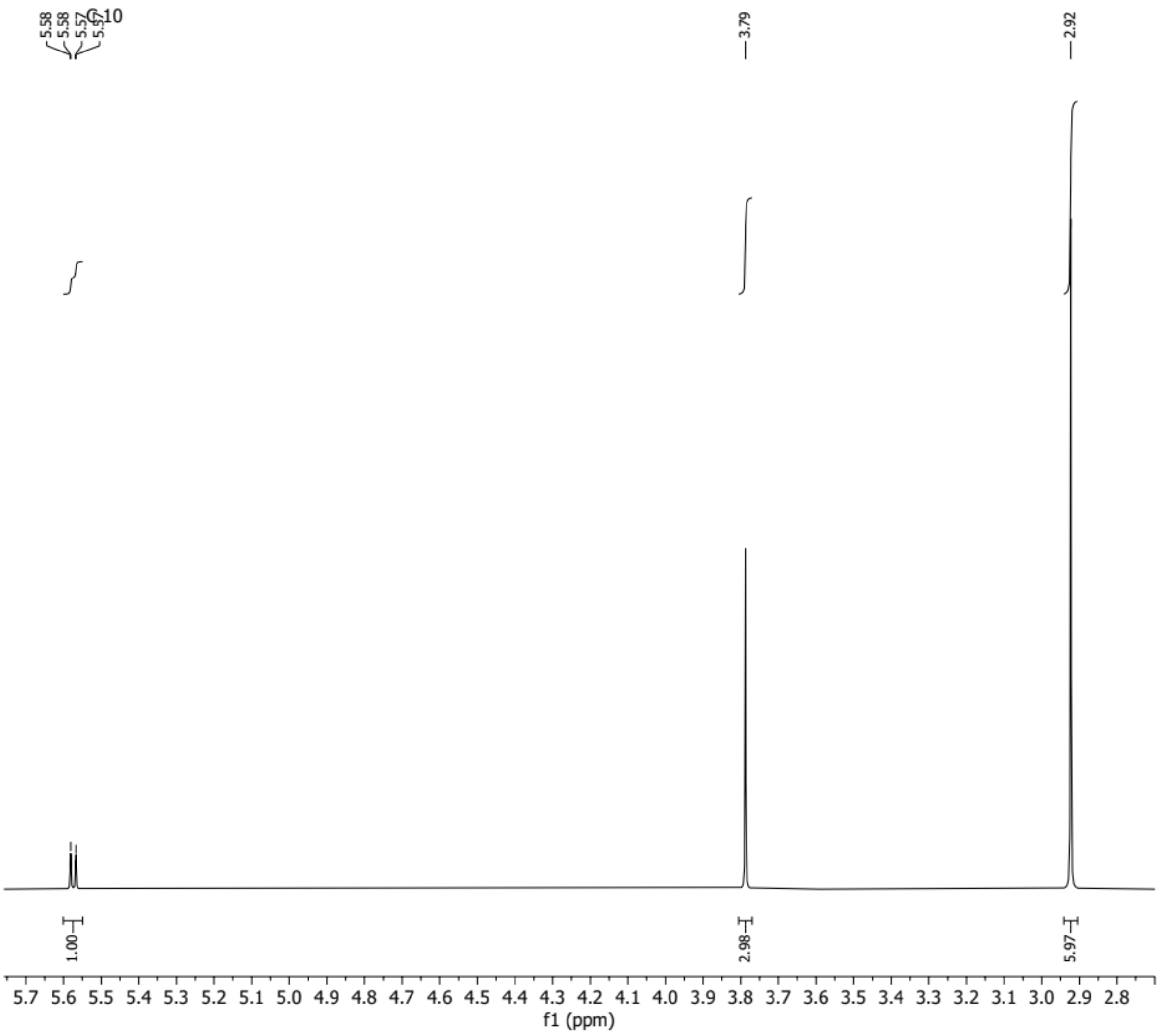

^{1}H NMR spectrum of synthesized compound C10

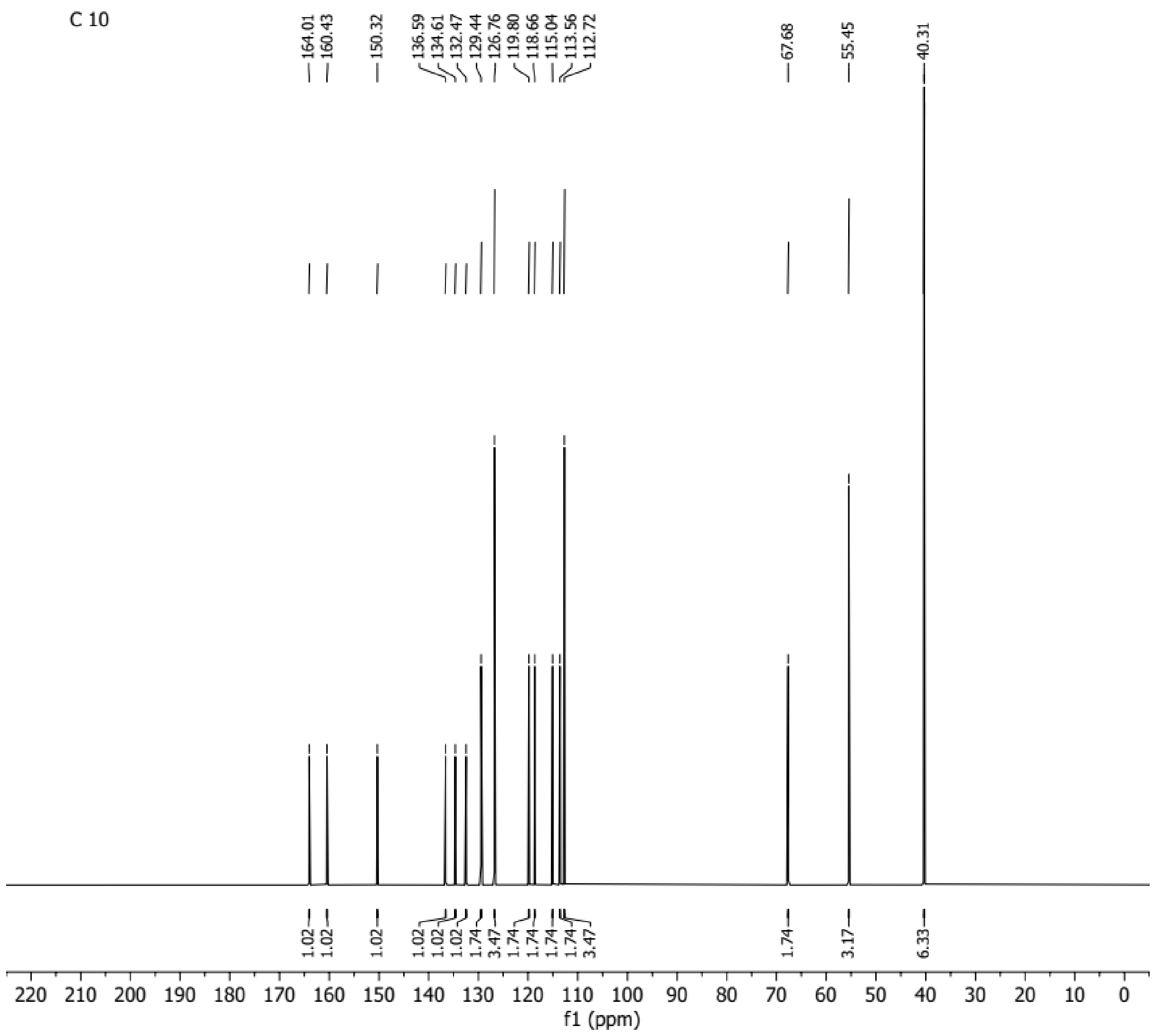

^{13}C NMR spectrum of synthesized compound C10
