# Supplementary material for: Synthesis, Spectroscopic Characterization, Molecular Docking Studies Toward the GABAA Receptor, ADMET Prediction, GABA Aminotransferase Inhibition, and Anxiolytic Evaluation of Novel Thiazine Derivatives
Source: Pharmaceuticals (Basel). 2026 Aug 28;19(9):1364. doi: 10.3390/ph19091364 (PMC13610616; doi:10.3390/ph19091364)
Supplement: Supplementary file 1 [file pharmaceuticals-19-01364-s001.zip › Table S1.pdf]

Table 3 in detailed

Table S1. Results of ADME and Toxicity evaluation of compounds (C1-C10).

**Parameter**	C1	C2	C3	C4	C5	C6	C7	C8	C9	C10
Molecular properties										
Molecular Weight	296.395	330.84	330.84	312.394	312.394	341.392	375.291	375.291	326.421	339.464
LogP	3.8389	4.4923	4.4923	3.5445	3.5445	3.7471	4.6014	4.6014	3.8475	3.9049
Rotatable Bonds	3	3	3	3	3	4	3	3	4	4
Acceptors	4	4	4	5	5	6	4	4	5	5
Donors	1	1	1	2	2	1	1	1	1	1
Surface Area	[illegible]28.09	138.393	138.393	132.884	132.884	142.743	141.957	141.957	139.568	146.579
**Absorption**										
Water solubility	-4.741	-5.539	-5.487	-4.346	-4.264	-5.132	-5.603	-5.603	-5.178	-5.144
Caco2 permeability	1.362	1.396	1.385	0.876	0.957	0.82	1.391	1.391	1.364	1.374
Intestinal absorption (human)	91.117	90.281	89.915	90.703	90.292	88.678	89.848	89.848	93.494	91.515
Skin Permeability	-2.659	-2.692	-2.667	-3.197	-3.132	-2.729	-2.667	-2.667	-2.828	-2.704
P-glycoprotein substrate	No	No	No	Yes	Yes	Yes	No	No	No	No
P-glycoprotein I inhibitor	Yes	Yes	Yes	No	No	Yes	Yes	Yes	Yes	Yes
P-glycoprotein II inhibitor	No	No	No	No	No	No	No	No	No	No
**Distribution**										
VDss (human)	0.307	0.382	0.35	0.26	0.194	0.16	0.366	0.366	0.308	0.357

Fraction unbound (human)	0.006	0	0	0.073	0.115	0	0	0	0.011	0
BBB permeability	0.19	0.141	0.148	-0.073	-0.081	-0.321	0.147	0.147	-0.054	0.143
CNS permeability	-1.32	-1.315	-1.322	-1.952	-1.961	-1.973	-1.322	-1.322	-1.947	-1.855
**Metabolism**										
CYP2D6 substrate	No	No	No	No	No	No	No	No	No	No
CYP3A4 substrate	Yes	Yes	Yes	Yes	No	Yes	Yes	Yes	Yes	Yes
CYP1A2 inhibitior	Yes	Yes	Yes	Yes	Yes	Yes	Yes	Yes	Yes	Yes
CYP2C19 inhibitior	Yes	Yes	Yes	Yes	Yes	Yes	Yes	Yes	Yes	Yes
CYP2C9 inhibitior	Yes	Yes	Yes	Yes	Yes	Yes	Yes	Yes	Yes	Yes
CYP2D6 inhibitior	Yes	No	No	No	No	No	No	No	No	No
CYP3A4 inhibitior	No	No	No	Yes	Yes	No	No	No	No	No
**Excretion**										
Total Clearance	0.103	-0.025	-0.03	-0.01	-0.014	0.126	-0.051	-0.051	0.133	0.075
Renal OCT2 substrate	Yes	Yes	Yes	No	No	No	Yes	Yes	Yes	Yes
**Toxicity**										
AMES toxicity	Yes	Yes	Yes	Yes	Yes	Yes	Yes	Yes	No	Yes
Max. tolerated dose (human)	0.288	0.245	0.237	0.268	0.185	0.014	0.234	0.234	0.222	0.202
hERG I inhibitor	No	No	No	No	No	No	No	No	No	No
hERG II inhibitor	Yes	Yes	Yes	Yes	Yes	No	Yes	Yes	No	Yes
Oral Rat Acute Toxicity (LD_{50})	2.395	2.619	2.577	2.166	2.102	2.345	1.142	2.584	2.607	2.522
Oral Rat Chronic Toxicity (LOAEL)	0.963	1.165	1.168	1.82	1.725	1.374	2.584	1.141	1.467	1.276

Hepatotoxicity	Yes	Yes	Yes	No	No	Yes	No	No	No	No
Skin Sensitisation	No	Yes	No	No	No	No	No	No	No	No
T.Pyriformis toxicity	1.842	2.013	1.983	1.437	1.317	1.14	1.989	1.989	1.931	1.838
Minnow toxicity	-0.029	-0.735	-0.162	-0.011	0.529	-0.702	-0.308	-0.308	-0.527	-0.132
